# Development of Isoniazid–Pyrazole Hybrids as Potential Antitubercular Agents

**DOI:** 10.3390/ijms27104385

**Published:** 2026-05-14

**Authors:** Mukanda Gedeon Kadima, Vinayak Singh, Gobind Kumar, Sahil Mishra, Pule Seboletswe, Ankit, Afsana Kajee, Françoise Roquet-Banères, Laurent Kremer, Rajshekhar Karpoormath, Parvesh Singh

**Affiliations:** 1 Discipline of Pharmaceutical Sciences, Westville Campus, University of KwaZulu-Natal, Durban 4000, South Africa; gedeonkadima@gmail.com; 2 Division of Medical Microbiology, Department of Pathology, Faculty of Health Sciences, Institute of Infectious Disease and Molecular Medicine (IDM), University of Cape Town, Rondebosch, Cape Town 7701, South Africa; vinayak.singh@uct.ac.za; 3 Discipline of Chemistry, Westville Campus, University of KwaZulu-Natal, P/Bag X54001, Durban 4000, South Africa; 223144174@stu.ukzn.ac.za (G.K.); sahil.mishra.2519@gmail.com (S.M.); seboletsweps@gmail.com (P.S.); 224196173@stu.ukzn.ac.za (A.); 4 National Health Laboratory Services (NHLS), Department of Microbiology, Inkosi Albert Luthuli Central Hospital, Durban 4000, South Africa; afsana.kajee@nhls.ac.za; 5 Centre National de la Recherche Scientifique UMR 9004, Institut de Recherche en Infectiologie de Montpellier (IRIM), Université de Montpellier, 1919 Route de Mende, 34293 Montpellier, France; francoise.roquet-baneres@irim.cnrs.fr (F.R.-B.); laurent.kremer@irim.cnrs.fr (L.K.); 6 INSERM, IRIM, 34293 Montpellier, France

**Keywords:** *Mycobacterium tuberculosis*, pyrazole, hydrazone, molecular hybrids, isoniazid, drug-likeness

## Abstract

A series of INH–pyrazole molecular hybrids (**6a**–**o**) was synthesized and evaluated for in vitro anti-tubercular activity against drug-susceptible, multidrug-resistant (MDR), and extensively drug-resistant (XDR) *Mycobacterium tuberculosis* strains, alongside their corresponding precursors (**4a**–**o**), using isoniazid (INH) as the reference drug. Overall, the hybrid compounds exhibited inhibitory activity comparable to or exceeding that of INH against the drug-susceptible strain. Among the series, compounds **6a**, **6d**–**6f**, and **6m** demonstrated the highest potency, with a minimum inhibitory concentration (MIC) of 0.9 µM, corresponding to an approximately 4.3-fold enhancement relative to INH. Compounds **6b**,**c**, **6g**–**i**, and **6l**,**m** also showed noticeable activity (MIC = 1.95 µM), representing an approximate twofold improvement over INH and significantly outperforming their respective precursors. Notably, compound **6o** exhibited enhanced activity against the XDR strain (MIC = 121 µM), reflecting an approximately 2.8-fold improvement compared to precursor **4o** (MIC > 341 µM), thereby highlighting the advantage of molecular hybridization. However, all compounds displayed diminished activity relative to INH against the resistant strains. Against the MDR strain, compounds **4h**, **6e**, and **6g** displayed measurable activity, with MIC values of 76, 125, and 112 µM, respectively. Cytotoxicity assessment using THP-1 human monocytic cells revealed low toxicity, with all tested compounds maintaining acceptable cell viability at 10 µg/mL. In addition, in silico ADME analysis indicated that the hybrid molecules comply with key drug-likeness criteria. Collectively, these findings suggest that INH–pyrazole hybrids represent promising lead scaffolds for the development of next-generation anti-tubercular agents.

## 1. Introduction

Tuberculosis (TB) remains a major global health threat, surpassing many other infectious diseases and continuing to be a leading cause of mortality among both immunocompetent individuals and those living with Human Immunodeficiency Virus infection [1]. The primary causative agent of TB is *Mycobacterium tuberculosis* (*Mtb*), an acid-fast bacterium. Although it primarily affects the lungs, resulting in pulmonary TB, the infection can disseminate to other organs, leading to extrapulmonary manifestations involving the spine, kidneys, brain, and other tissues [2,3]. Currently, approximately one-third of the global population suffers from latent TB infections (LTBI), a condition in which the bacterium remains dormant without causing clinical symptoms. However, if left untreated, LTBI can progress to an active disease, making treatment significantly more challenging [4]. *Mtb* can survive within host cells by transitioning between latent and active states and undergoing genetic mutations, thereby evading host immune defenses. This adaptability has contributed to the emergence of drug-resistant forms of TB, including multidrug-resistant TB (MDR-TB), extensively drug-resistant TB (XDR-TB), and totally drug-resistant TB (TDR-TB) [5].

Approximately 70,000 people are infected annually by drug-resistant strains of *Mtb* [6]. MDR-TB is defined as in vitro resistance to at least isoniazid (INH) and rifampicin (RIF), the two most potent first-line anti-TB drugs. Conversely, XDR-TB is characterized by additional resistance to at least one fluoroquinolone and one injectable second-line anti-TB drug in addition to INH and RIF [7,8]. Clinical outcomes for MDR- and XDR-TB are largely suboptimal with treatment regimens that are prolonged, toxic, and expensive. These difficult-to-treat cases pose a serious threat to global TB control and elimination efforts [9,10]. This growing resistance has created an urgent need for the development of new and effective antitubercular agents.

**Figure 1:**
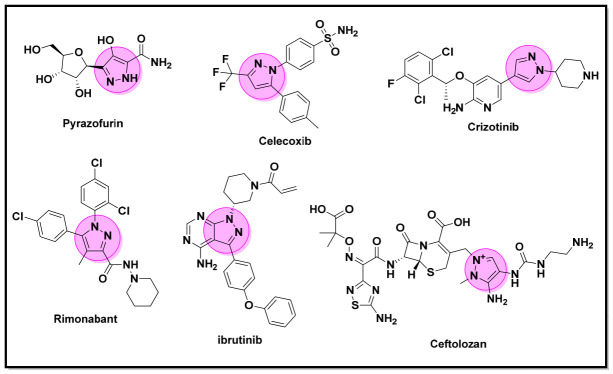
Pyrazole-based marketed drugs.

Among various chemical scaffolds, pyrazoles have gained significant attention due to their broad spectrum of promising biological activities that include anti-tubercular [11], antiviral [12], anti-fungal [13], antibacterial [14], antidiabetic [15], antihyperglycaemic [16], antiandrogenic [17], anti-inflammatory [18,19] and anticonvulsant [20] activities. Figure 1 presents several well-known, best-selling drugs on the market that incorporate the pyrazole scaffold as a key structural motif.

INH, along with RIF, Pyrazinamide, and Ethambutol, is classified as a first-line agent for the treatment of TB [21]. Among these, INH is the most widely used due to its potent activity against *Mtb* [22]. Its primary target is enoyl-acyl carrier protein (ACP) reductase (InhA), a key enzyme involved in mycolic acid synthesis, which is essential for the integrity of the bacterial cell wall [23]. INH is a prodrug that requires activation by the catalase-peroxidase enzyme *KatG*, which facilitates the formation of an INH-NAD^+^ adduct [24]. This adduct binds covalently to the active site of InhA, thereby inhibiting its enzymatic activity, disrupting cell wall biosynthesis, and ultimately causing bacterial cell death [25]. However, prolonged use of INH can lead to the emergence of drug-resistant *Mtb* strains, particularly those with mutations in either the *KatG* gene (impairing INH activation) or the InhA gene (reducing binding affinity of the adduct). These mutations significantly reduce the effectiveness of INH, posing a major challenge in TB treatment [26].

According to the World Health Organization (WHO) reports, approximately 8% of TB patients have INH-resistant TB. Given the rapid emergence of drug-resistant *Mtb* variants, the development of novel anti-TB agents has become imperative [27]. A promising approach in this regard involves the chemical modification of existing anti-TB scaffolds aimed at improving their efficacy, particularly against these strains [28].

**Figure 2:**
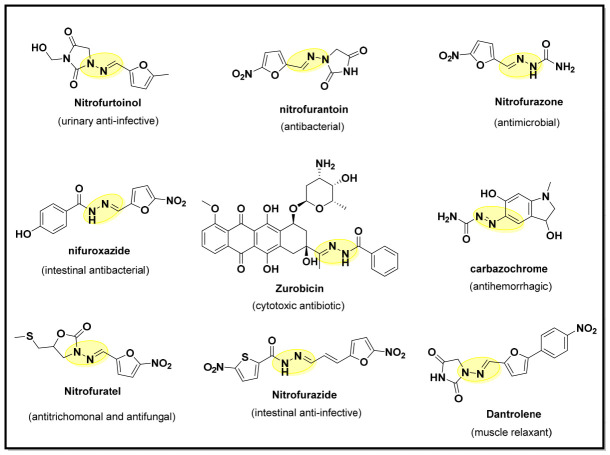
The bioactive compounds containing the hydrazone moiety.

Hydrazone is a highly versatile pharmacophoric unit with well-documented antimicrobial, anticonvulsant, anti-inflammatory, antitubercular, and antitumor properties [29,30]. This functional group is a key component of several FDA-approved drugs, including nitrofurtoinol (a urinary anti-infective), nitrofurantoin (a broad-spectrum antibacterial), nitrofurazone (an antimicrobial agent), nifuroxazide (an intestinal antibacterial), zurobicin (cytotoxic antibiotic), carbazochrome (antihemorrhagic), nitrofuratel (an anti-trichomonal and antifungal agent), nifurzide (an intestinal anti-infective), carbazochrome (an anti-hemorrhagic drug), and dantrolene (a muscle relaxant) (Figure 2) [31,32].

Molecular hybridization (MH) has emerged as a powerful strategy in medicinal chemistry for drug design and discovery [33,34,35]. This approach involves the integration of two or more biologically active pharmacophores into a single molecular framework, with or without the use of spacers [15,36]. In many cases, this technique has facilitated the creation of molecular hybrids that demonstrate superior pharmacological and pharmacodynamic properties compared to their parent compounds.

INH-derived compounds incorporating nitrogen-containing heterocyclic fragments have demonstrated promising anti-TB activity [37,38,39,40,41,42]. For instance, Nayak et al. [37] developed a series of twenty INH–pyrazole derivatives, identifying *N*-(2-chlorobenzoyl)-*N′*-{(1*E*)-[3-(3-chlorophenyl)-1*H*-pyrazol-4-yl]methylene}isonicotinohydrazide as a particularly effective candidate. This compound showed notable anti-TB activity against the *Mtb* H37Rv strain, with an MIC of 1.7 mM compared to 0.7 mM for standard INH. Similarly, Badar et al. [38] reported the synthesis and characterization of new INH–1,2,3-triazole derivatives, among which the compound (E)-N′-(3,5-dichloro-2-((1-(2-chlorophenyl)-1H-1,2,3-triazol-4-yl)methoxy)benzylidene)isonicotinohydrazide exhibited notable potency, with a minimum inhibitory concentration (MIC) of 1.56 μg/mL against the *Mtb* H37Rv strain. In another study, Oliveira et al. [42] synthesized hydrazone derivatives of INH containing phenolic and heteroaromatic moieties. Two compounds, (E)-N′-((4-hydroxyphenyl)methylene)isonicotinohydrazide and (E)-N′-((1H-pyrrolo[2,3-b]pyridin-3-yl)methylene)isonicotinohydrazide, demonstrated superior activity, with MIC values of 0.05 μM and 0.056 μM, respectively, outperforming standard INH (MIC = 0.18 μM) against the H37Rv strain. 

In recent years, our group has focused on developing novel INH derivatives incorporating pyrimidinone moieties via a hydrazone linker. These compounds were evaluated against the drug-sensitive *Mtb* H37Rv strain, where derivative ethyl (E)-4-(2,4-difluorophenyl)-6-((4-((2-isonicotinoylhydrazono)methyl)phenoxy)methyl)-2-oxo-1,2,3,4-tetrahydropyrimidine-5-carboxylate (MIC = 0.39 μg/mL) exhibited comparable potency with INH (MIC = 0.039 μg/mL) [41]. In another study, flexible benzyl-linked analogs and rigid sulfonate ester derivatives of INH displayed MIC values in the range of 0.078–0.156 μg/mL [40]. In addition, a series of aryl sulfonamide-linked INH hydrazone hybrids, designed as InhA inhibitors, showed significantly enhanced antimycobacterial activity, with representative compounds exhibiting MIC values as low as 0.156 μg/mL [39].

**Figure 3:**
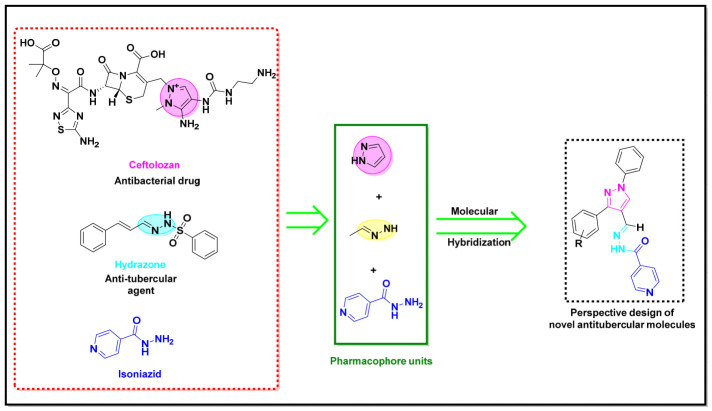
The rationalization of INH–pyrazole-based molecular hybrids.

Building on the aforementioned considerations, we designed and synthesized a series of INH–pyrazole-based molecular hybrids (Figure 3) to enhance their anti-TB efficacy. To systematically investigate structure–activity relationships, and also based on our previous studies, a range of electron-donating and electron-withdrawing substituents were introduced at the phenyl ring in both *meta* and *para* positions. This design aimed to evaluate the influence of electronic and steric factors on antimycobacterial activity of INH-based hydrazones. The synthesized compounds were structurally characterized using a combination of spectroscopic techniques, including Fourier-transform infrared spectroscopy (FTIR), nuclear magnetic resonance (NMR), and high-resolution mass spectrometry (HRMS), to confirm their purity and molecular structures. Following characterization, all compounds were evaluated for their activity against INH-sensitive *Mtb* as well as XDR and MDR strains. In addition, cytotoxicity was assessed using the THP-1 human monocytic cell line. The drug-likeness of the synthesized hybrids was further predicted through in silico analyses.

## 2. Results and Discussion

### 2.1. Chemistry

**Scheme 1:**
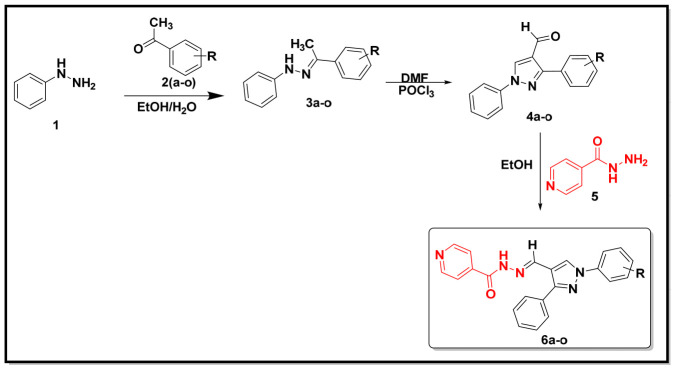
Synthetic route for compound **6a**–**o**.

The molecular hybrids (**6a**–**o**) were synthesized through a multistep reaction protocol, as illustrated in Scheme 1. In the first step, phenylhydrazine imines (**3a**–**o**) were prepared via a condensation reaction between phenylhydrazone **1** and substituted acetophenones **2**. Subsequently, the Vilsmeier–Haack reaction was employed to afford the corresponding pyrazole aldehydes (**4a**–**o**). Notably, the amine functionalities in precursors **4b** and **4c** underwent a side reaction with dimethylformamide (DMF), leading to the formation of the corresponding imines, as confirmed by their NMR spectroscopic analysis (Supplementary Materials). This observation suggests that DMF acted as a transient protecting agent for the amine groups, temporarily attenuating their reactivity during the synthetic sequence. This unexpected yet useful interaction highlights the dual role of DMF, functioning not only as a solvent but also as a reversible protecting group under the applied reaction conditions used. Finally, the target isonicotinohydrazide–pyrazole hybrids (**6a**–**o**) were obtained via the condensation of pyrazole aldehydes with INH in ethanol, affording good to excellent yields (74–95%). Importantly, for compounds **6b** and **6c**, the results confirmed that the amine groups were deprotected in the final step, indicating that the imine protection observed in intermediates (**4b** and **4c**) was reversible under the reaction conditions, thereby restoring the free amine functionality in the final hybrid structures.

The structures of the molecular hybrids were characterized using different spectroscopic techniques such as FTIR, NMR (^1^H and ^13^C), and HRMS. For example, the FT-IR spectrum of compound **6g** displayed three characteristic absorption peaks: a broad N-H stretch at 3046 cm^−1^, a carbonyl (NHC=O) stretch at 1659 cm^−1^, and a C=N stretch at 1546 cm^−1^. The proton NMR spectrum of compound **6g** exhibited key signals consistent with its proposed structure. A characteristic downfield signal at δ 12.04 ppm was observed, corresponding to the NH proton, indicative of hydrogen bonding or deshielding effects. The iminic proton (N=CH) appeared at δ 8.67 ppm, confirming the formation of the hydrazone linkage. Additionally, a signal at δ 2.48 ppm was assigned to the methyl (-CH_3_) group, consistent with its presence in an electron-rich environment. Furthermore, the ^13^C NMR spectral data of compound **6g** supported its proposed structure, displaying a characteristic peak for the carbonyl carbon at δ 161.52 ppm, indicative of the isonicotinoyl moiety. The methyl carbon appeared at δ 21.33 ppm, consistent with its aliphatic nature. In addition, the spectrum exhibited the expected number of carbon signals corresponding to aromatic, pyrazole, and aliphatic carbons, further confirming the successful synthesis and structural assignment of compound **6g**. Finally, the HRMS of compound **6g** (calculated 404.1486 and found 404.1493) also supported the assigned structure. Notably, the synthesized isonicotinohydrazide–pyrazole molecular hybrids (**6a**–**o**) exhibited a mixture of *E* and *Z* isomers, consistent with trends reported in the literature for similar N-acyl hydrazone frameworks [31]. The appearance of a singlet corresponding to the =CH proton in the range of 8.41–8.67 ppm for compounds **6a**–**o** suggests the possible formation of the *E*-isomer as the major isomer, likely due to its greater thermodynamic stability relative to the corresponding Z-isomer [39,40,41]. However, the configuration cannot be assigned with certainty based solely on the proton NMR data. Accordingly, the *E*/*Z* (or major/minor) isomer ratio for compound **6a** was determined to be 93:7 based on the proton NMR spectrum. Similarly, for the remaining compounds (**6b**–**o**), the major isomer constituted 92–94%, while the minor isomer accounted for 6–8%. Efforts to separate the individual isomers using conventional chromatographic techniques, including column chromatography and preparative thin-layer chromatography, were unsuccessful. This is likely due to the very similar polarity and physicochemical properties of the isomers, as well as their propensity for rapid interconversion in solution. Similar observations have been reported in earlier studies, where hydrazone derivatives exist in dynamic equilibrium, making isolation of pure isomers challenging without advanced techniques such as low-temperature NMR or crystallization-induced separation [43,44].

### 2.2. Biological Studies

The biological evaluation was therefore performed on the equilibrated *E*/*Z* mixtures, which are expected to represent the biologically relevant form under physiological conditions. Literature evidence also suggests that the *E*-isomer is generally thermodynamically more stable due to reduced steric hindrance and may predominate in solution [39,40,41]. However, due to the inability to isolate individual isomers in the present work, the specific contribution of each isomer to antitubercular activity could not be determined.

#### 2.2.1. In Vitro Activity Against INH-Sensitive, XDR and MDR *M. tuberculosis* Strains

The synthesized novel series molecular hybrids (**6a**–**o**), along with their pyrazole aldehyde precursors (**4a**–**o**), were evaluated for their in vitro activity against *Mtb* H37RvMA (ATCC 27294). The assay was conducted in Middlebrook 7H9 medium [45], using INH as a reference drug [46]. The Minimum inhibitory concentration (MIC) values for all tested compounds are summarized in Table 1.

The results presented in Table 1 revealed that the pyrazole aldehyde precursors (**4a**–**o**) exhibited moderate to low anti-TB activity, with MIC values ranging from 31.25 to >62.5 µM. Notably, compounds **4a** and **4e** demonstrated the highest activity among the precursors, each with an MIC of 31.25 µM. In contrast, the majority of the compounds (**4b**–**j**, **4l**, and **4n**,**o**) showed weaker activity, with MIC values ≥ 62.5 µM, indicating that the precursor aldehydes alone are significantly less potent. In addition, the compounds were evaluated against drug-resistant strains (MDR and XDR) of *Mtb*. The MDR strain possesses a mutation in *KatG* (conferring resistance to INH) and in *rpoB* (conferring resistance to rifampicin), whereas the XDR strain exhibited mutations in *KatG*, gyrA (conferring resistance to fluoroquinolones), and mutations in *atpE* (conferring resistance to bedaquiline). The results summarized in Table 1 indicated that the two tested compounds, **4a** (MIC > 403 μM) and **4o** (MIC > 341 μM**)**, exhibited weak activity against the XDR strain. Against the MDR strain, only compound **4h** demonstrated moderate activity, with an MIC of 76 µM, making it the most active among the series 4 compounds against resistant strains. These findings highlight the limited efficacy of the pyrazole aldehyde precursors against the resistant TB strains.

The INH–pyrazole hydrazone molecular hybrids (**6a**–**o**) exhibited markedly enhanced anti-TB activity, with MIC values ranging from 0.9 µM to 3.9 µM. Notably, five compounds (**6a**, **6d**, **6e**, **6f**, and **6m**) emerged as the most potent, each displaying an MIC of 0.9 µM, representing approximately a 69-fold increase in potency compared to their corresponding precursors (**4a**, **4d**–**f**, and **4l**), which exhibited MIC values in the range of 31.25–62.5 µM. These results strongly underscore the impact of molecular hybridization, where conjugating the INH pharmacophore with the pyrazole ring leads to a synergistic enhancement in anti-TB activity. Remarkably, these compounds showed ~4.3-fold greater inhibitory potency compared to the standard drug INH (MIC = 3.9 µM). Most compounds from this series (**6b**,**c**, **6g**–**i**, and **6k**,**l**) displayed an MIC of 1.95 µM, approximately ~32-fold and ~2-fold more potent than their respective precursors and the INH. Three compounds (**6j**, **6n**,**o**) with an MIC of 3.9 µM showed anti-TB activity equivalent to the INH yet exhibited multiple-fold superior activity to their precursors.

Testing of the INH–pyrazole hybrids against XDR and MDR strains revealed a reduction in activity against XDR strains following the inclusion of INH (Table 1). Nevertheless, compound **6o** showed a slight improvement, with an MIC of 121µM, representing a ~2.8-fold increase in potency compared to its precursor **4o** (MIC > 341 µM), indicating that molecular hybridization still offered some advantage in this case.

Against the MDR strain, five compounds, **6d**, **6e**, **6h 6j** and **6k**, demonstrated notable antitubercular activity, with MIC values of >262, 125, 112, >249, and >259 µM, respectively. Among these, **6e** and **6h** were the most effective, suggesting that certain hybrid structures retain or even enhance efficacy against drug-resistant *Mtb* strains. Overall, the INH–pyrazole molecular hybrids exhibited significantly superior anti-TB activity compared to their corresponding pyrazole aldehyde precursors, clearly demonstrating the crucial role of the INH pharmacophore in enhancing biological efficacy.

#### 2.2.2. Structure–Activity Relationship (SAR)

**Figure 4:**
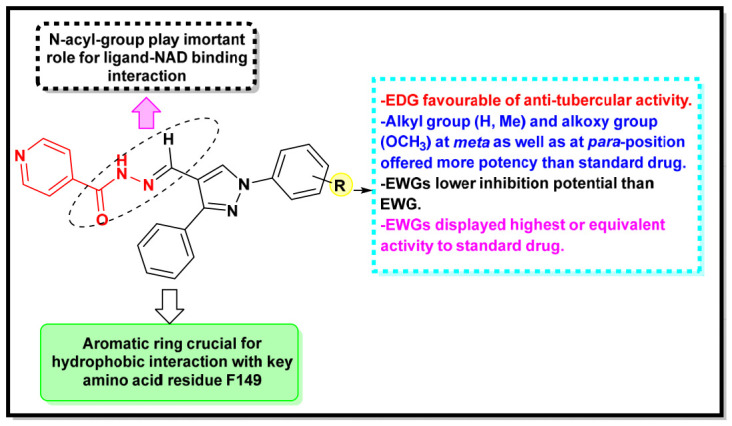
A brief SAR of the INH–pyrazole hybrids.

A structure–activity relationship (SAR) analysis was conducted to identify trends in the observed biological activity in relation to the structural features of the tested compounds (Figure 4). To critically analyze these findings, the results were compared with previously reported INH-based N-acyl hydrazone derivatives, which are well-established antitubercular scaffolds with well-documented SAR dependence on aromatic substitution patterns and electronic effects on biological activity.

In the 4-series, most compounds exhibited weak antimycobacterial activity, with MIC values generally exceeding 62.5 µM. Compound **4a**, which lacks any substituent on the phenyl ring, showed the highest activity within the series (MIC = 31.25 µM). The introduction of electron-donating groups (EDGs) on the phenyl ring, such as in **4b** (*p*-amino, MIC > 62.5 µM), **4c** (*m*-amino, MIC > 62.5 µM), and **4d** (*p*-methyl, MIC > 62.5 µM), generally resulted in diminished activity. An exception was compound **4e** (*p*-methoxy, MIC = 62.5 µM), which demonstrated comparable inhibitory activity to **4a**, suggesting that the *para*-methoxy substitution may modestly preserve activity. The placement of the same group (**4f**) at the *meta* position, however, decreased the activity. The introduction of electron-withdrawing groups (EWGs) in **4g (***p*-Br), **4i** (*p*-Cl), **4k** (*p*-F), **4n** (*p*-CF_3_)_,_ **4o** (*p*-NO_2_), regardless of their position, also did not improve the activity (MIC = >62.5 µM). However, compounds bearing disubstituted EWGs, **4m** (2,4-difluoro), with MIC = 62.5 µM demonstrated enhanced activity compared to their mono-substituted counterparts.

In the 6-series, introducing the INH moiety demonstrated significantly enhanced antimycobacterial activity, confirming the essential role of INH in mediating interaction with InhA. Compound **6a** (MIC = 0.9 µM), which bears no substitution on the phenyl ring, exhibited the highest potency among all the compounds tested. This compound was approximately 4.3-fold more potent than INH (MIC = 3.9 µM), representing a significant improvement in activity. This is strongly consistent with the findings of Vicini et al. (2006), wherein unsubstituted INH-derived hydrazones showed the most balanced antimycobacterial activity due to optimal steric and electronic compatibility with the target binding pocket, likely involving InhA-associated interactions, supporting the role of the unsubstituted phenyl core as an optimal pharmacophore [47]. The anti-tubercular activity remained largely unaffected upon the introduction of weak electron-donating groups (EDGs) at both the *meta*- and *para*-positions, as observed for compounds **6f** (*m*-methoxy), **6d** (*p*-methyl), and **6e** (*p*-methoxy), indicating that such substitutions exert minimal influence on antimycobacterial potency. This observation is consistent with previous SAR reports, which suggest that weak EDGs are generally tolerated in INH-based hydrazone frameworks [48]. However, incorporation of stronger electron-donating substituents, such as amino groups, at the *meta* (**6b**) and *para* (**6c**) positions resulted in a reduction in activity (approximately 2.1-fold). This decline may be attributed to excessive electron donation, which could perturb binding interactions with the biological target. Despite this decrease, these compounds still exhibited superior activity compared to the standard drug INH. The introduction of EWGs generally resulted in lower antimycobacterial activity compared to EDGs, except compound **6m** (3,4-difluoro), which demonstrated potency comparable to the unsubstituted compound **6a**. This observation reinforces previously reported advantages of multi-halogen substitution, likely due to synergistic electronic and lipophilic effects [41]. As shown in Table 1, mono-substituted halogens at the *para*- (**6g** = *p*-Br, **6i** = *p*-Cl, **6k** = *p*-F) and *meta*-positions (**6h** = *m*-Br, **6j** = *m*-Cl, **6l** = *m*-F) exhibited comparable activity profiles, each displaying an MIC value of 1.95 µM. This trend suggests that halogen substitution is well tolerated irrespective of positional variation, likely due to a balanced contribution of electronic and lipophilic effects. In particular, halogens may enhance lipophilicity and membrane permeability without significantly perturbing key binding interactions, consistent with a previous report [41]. Furthermore, compounds bearing strong EWGs at the *para*-position, such as **6n** (*p*-CF_3_) and **6o** (*p*-NO_2_), demonstrated activity comparable to the disubstituted halogen derivatives but were less potent than the unsubstituted analog **6a**.

Overall, the SAR analysis indicates that the EDGs generally enhance antimycobacterial activity, particularly when integrated with the INH moiety. In contrast, bulky or strongly electron-withdrawing substituents tend to diminish biological potency.

#### 2.2.3. In Vitro Activity Against INH-Resistant *M. tuberculosis* Strains

A comparative analysis of the MIC values for the tested compounds and INH against the *Mtb* H37RvMA (ATCC 27294) strain reveals promising baseline antimycobacterial activity. While INH exhibits a lower MIC of 1.95 µM, indicating a slightly higher in vitro potency, all tested compounds (**6a**, **6d**, **6e**, **6f**, and **6n**, Table 2) demonstrate comparable activity with a consistent MIC of 3.9 µM. This suggests that these compounds possess inherent anti-mycobacterial activity against the susceptible strain. However, these compounds, including INH, demonstrate significantly elevated MICs across all three tested mutant strains (Table 2). Specifically, INH shows an MIC of >62.5 µM of the strain carrying a W198 point mutation in *KatG*_W198 mutant, 7.8–3.9 µM against the strain with a t198a mutation in the *KatG* promoter, and 7.8 µM against the c15t_InhA mutant. These elevated MICs confirm the established resistance phenotype of these strains to INH. It is noteworthy that the MICs for the mutants carrying the *KatG* (t198a) and c15t_InhA mutations are numerically lower than for the mutant with the *KatG*_W198, suggesting varying degrees or mechanisms of INH resistance, where *KatG*_W198 confers the highest level of resistance. All tested compounds exhibit MICs > 62.5 µM against this specific *KatG*_W198 mutant, mirroring the complete loss of activity observed for INH against this strain. Against the *KatG* (t198a) mutant, compounds **6a**, **6d**, and **6f** exhibit MICs of 15.6 µM, while compound **6e** shows an MIC of 31.3 µM. In contrast, compound **6n** remained inactive (MIC > 62.5 µM). While compounds **6a**, **6d**, **6e**, and **6f** demonstrate some retained activity against this specific *KatG* mutant compared to the *KatG*_W198 variant, their MICs (15.6–31.3 µM) are higher than INH’s (7.8–3.9 µM) against the same strain. Against the InhA mutant (c15t_fabG1InhA), compounds **6a** and **6f** show MICs of 31.3 µM, whereas compounds **6d** and **6e** demonstrated comparatively improved activity with MIC values of 15.6 µM. Compound **6n** continues to show broad ineffectiveness with an MIC >62.5 µM. Relative to INH (MIC = 7.8 µM), these compounds displayed reduced potency, further indicating limited ability to overcome InhA-associated resistance mechanisms.

Based on the fold-change analysis, compounds **6a**, **6d**, **6e**, and **6f** emerged as the most promising candidates within the tested series. These compounds exhibited retained, albeit reduced, activity against the *KatG* (t198a) mutant (4.0–8.0-fold-change) and the c15t_InhA mutant (4.0–8.0-fold-change), in contrast to their complete loss of activity against the *KatG*_W198 mutant (>16.0 fold-change). Specifically, compounds **6d** and **6e** showed marginally improved potency against the InhA mutant (MIC 15.6 µM, 4.0-fold-change) compared to compounds **6a** and **6f** (MIC 31.3 µM, 8.0-fold-change). However, their MIC values remain higher than that of INH (7.8 µM), indicating that none of the analogs surpass INH in efficacy against this resistance mechanism. In contrast, compound **6n** consistently exhibited a pronounced loss of activity across all tested mutant strains (>16.0-fold change), thereby identifying it as a non-promising candidate within the series.

The observed resistance profile, characterized by pronounced susceptibility loss against the *KatG*_W198 mutant but retained, albeit reduced, activity against the *KatG* (t198a) and InhA mutants, suggests a nuanced interaction of compounds **6a**, **6d**, **6e**, and **6f** with established INH resistance pathways. The complete loss of activity against *KatG*_W198 indicates a strong dependence on functional *KatG*-mediated activation or a highly sensitive downstream target. The retained but reduced activity against *KatG* (t198a) suggests that this specific mutation has a less severe impact on the compounds’ efficacy compared to W198, or that the compounds can partially bypass the t198a defect. For future drug design, understanding these specific interactions with different resistance mutations is paramount.

### 2.3. In Vitro Cytotoxicity Studies

**Figure 5:**
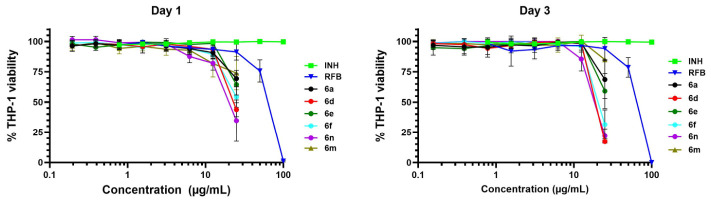
Cytotoxicity assay of compounds **6a**, **6d**–**f**, **6m**, and **6n** in THP-1 cells. The cells were differentiated into macrophages with PMA for 48 h and then exposed to increasing concentrations of each compound (starting from 0.195 μg/mL to 25µg/mL) for an additional (Left side) 24 h (Day 1) or (Right side) 72 h (Day 3) at 37 °C with 5% CO_2_. DMSO served as the negative control, while 20% SDS was the positive control for each plate. INH and Rifabutin (RFB) were used as internal controls. The results represent the mean +/− SD of three independent experiments conducted in duplicate.

The cytotoxicity of the five most active compounds, **6a**, **6d**–**f** and **6n**, was evaluated in THP-1 cells using the resazurin assay (Figure 5). Untreated cells and cells exposed to the compound solvent, dimethyl sulfoxide (DMSO), served as viability controls (i.e., non-cytotoxic conditions), while 20% sodium dodecyl sulfate (SDS) was used as a cytotoxic control. INH was included as an internal reference compound, and rifabutin (RFB) was employed as an additional control in cytotoxicity assays. All experiments were performed in duplicate and independently repeated three times. Dose–response curves were generated, where applicable, and analyzed by nonlinear regression using GraphPad Prism 10. The tested compounds exhibited strong anti-*Mtb* activity, with relatively high selectivity indices (SI > 15) on day 1. An exception was compound **6n**, which showed a lower SI value (SI = 12.5). By day 3, SI values decreased slightly (SI < 15), indicating a modest increase in cytotoxicity over time. These findings suggest that, although the compounds initially display favorable efficacy relative to host cell toxicity, their selectivity diminishes with prolonged exposure. However, the calculated CC_50_ values should be interpreted with caution, as the experimental data do not consistently cover the full sigmoidal dose–response range. This limitation compromises the accuracy of nonlinear regression modeling and may explain why some SI values could not be reliably determined.

### 2.4. In Silico Prediction of Drug-Likeness

The ADME Swiss online web server was used to predict the drug-likeness properties of the synthesized INH–pyrazole hybrids. In view of this, the most active compounds **6a**, **6d**–**6f**, and **6n** were selected for their drug-likeness and ADME profiling and the results are presented in Table 3. As can be seen in Table 3, all these compounds complied with the standard range, including INH, suggesting their good oral bioavailability. These compounds may make good drug candidates based on their in silico drug-likeness and ADME properties.

## 3. Materials and Methods

### 3.1. Chemicals and Reagents

All the chemicals utilized to synthesize the precursors and molecular hybrids were purchased from Merck (Merck KGaA, Durban, South Africa), Sigma-Aldrich (Sigma-Aldrich, Durban, South Africa), and CRD, and were further used without any purification. TLC 60 F254 silica gel plates (Merck KGaA, Durban, South Africa) were used to monitor the reaction progress. A Bruker Avance III NMR spectrometer operated using TopSpin software (v4.1, Bruker Corporation, Durban, South Africa) spectrometer was used to record ^1^H (600 MHz) and ^13^C (150 MHz) NMR spectra of the compounds. The multiplicities are reported as follows: singlet (s), doublet (d), doublet of doublets (dd), multiplet (m), triplet (t), and broad singlet (brs) and coupling constant (*J*) in Hz. Infrared (IR) spectra were obtained on Perkin Elmer Spectrum BX FT-IR (Beaconsfield, UK) and reported in the range of 4000–400 cm^−1^ using an ATR accessory (Pike Technologies) with a diamond crystal. The High-resolution mass analysis spectra were obtained using Waters Micromax LCT Premier TOF-MS instrument (PMB, Durban, South Africa) using a 1–2 ppm concentration of the sample. Column chromatography was performed on silica gel (60–120 mesh).

### 3.2. General Procedure for Synthesis of Precursors ***3a**–**o***

The starting materials **3a**–**o** were prepared by stirring a mixture of substituted acetophenones 1 (1.0 eq.) and dry phenyl hydrazine (1.5 eq.) in water and ethanol in a round-bottom flask at room temperature for 30 min. A catalytic amount of concentrated H_2_SO_4_ was used to promote the progress of the reaction. After the reaction was complete, the resulting solution was filtered and washed with water.

### 3.3. General Procedure for the Synthesis of Precursors ***4a**–**o***

A combination of substituted phenylhydrazones (1.0 eq.) and phosphoryl chloride (3.0 eq.) was combined in a round-bottom flask, stirred in cold DMF, and then refluxed at 60 °C for 6 h. After the reaction was finished, the solution was poured over crushed ice and neutralized with 10% NaHCO_3_. The resulting solid was filtered and washed with water to obtain compound **7**.

### 3.4. General Procedure for the Synthesis of Molecular Hybrids ***6a**–**o***

A 50 mL single-neck round-bottom flask was filled with INH (1.0 eq.) and pyrazoles **4a**–**o** (1.0 eq.). The mixture was then heated under reflux in ethanol at 78 °C for 2 h and 30 min. A catalytic amount of glacial acetic acid was added to promote the reaction progress. After the reaction was complete, the solvent was removed using a rotary evaporator. The resulting solid was filtered and rinsed with water and then dried.

### 3.5. Analytical Data

The analytical data of the synthesized hybrids (**6a**–**o**) and precursors (**4a**–**o**) have been provided in the below section and all the spectral data provided in the Supporting Information.

#### 3.5.1. Analycal Data of Series 4

##### 1,3-Diphenyl-1H-pyrazole-4-carbaldehyde (**4a**)

Appearance: White solid. Yield: 88%. ^1^H-NMR (600 MHz, DMSO-d6) δ 7.42 (t, *J* = 7.38 Hz, 1H), 7.48–7.53 (m, 3H), 7.56 (t, *J* = 8.12 Hz, 2H), 7.92 (d, *J* = 6.71 Hz, 2H), 7.99 (d, *J* = 7.74, 2H), 9.32 (s, 1H), 9.98 (s, 1H). ^13^C-NMR (150 MHz, DMSO-d6) δ 119.71, 122.61, 128.20, 129.02, 129.16, 129.66, 130.18, 131.71, 135.29, 139.05, 153.15, 185.11.

##### 3-(4-Aminophenyl)-1-phenyl-1H-pyrazole-4-carbaldehyde (**4b**)

Appearance: White solid. Yield: 71%. ^1^H-NMR (600 MHz, DMSO-d6) δ 2.94 (s, 3H), 3.04 (s, 3H), 7.03 (d, *J* = 7.77 Hz, 2H), 7.40 (t, *J* = 7.40 Hz, 1H), 7.55 (t, *J* = 7.49 Hz, 2H), 7.81 (t, *J* = 10.01 Hz, 3H), 7.98 (d, *J* = 7.97 Hz, 2H), 9.28 (s, 1H), 9.98 (s, 1H). ^13^C-NMR (150 MHz, DMSO-d6) δ 34.50, 119.60, 121.26, 122.44, 124.89, 128.03, 129.83, 130.16, 135.22, 139.10, 153.41, 153.44, 154.36, 185.14.

##### 3-(3-Aminophenyl)-1-phenyl-1H-pyrazole-4-carbaldehyde (**4c**)

Appearance: White solid. Yield: 75%. ^1^H-NMR (600 MHz, DMSO-d6) δ 3.11 (s, 3H), 3.20 (s, 3H), 7.32 (s, 1H), 7.42–7.48 (m, 2H), 7.56 (t, *J* = 7.47 Hz, 1H), 7.64 (d, *J* = 6.92 Hz, 1H), 7.69 (t, *J* = 9.71 Hz, 1H), 7.99 (d, *J* = 8.07 Hz, 2H), 8.29 (d, *J* = 8.97 Hz, 2H), 9.32 (s, 1H), 10.00 (s, 1H). ^13^C-NMR (150 MHz, DMSO-d6) δ 35.73, 36.16, 119.75, 121.41, 122.65, 124.63, 128.26, 129.94, 130.20, 132.64, 134.90, 139.02, 153.08, 154.36, 160.34, 185.31.

##### 3-(4-Aminophenyl)-1-phenyl-1H-pyrazole-4-carbaldehyde (**4d**)

Appearance: White solid. Yield: 99%. ^1^H-NMR (600 MHz, DMSO-d6) δ 2.37 (s, 3H), 7.31 (d, *J* = 7.86 Hz, 2H), 7.41 (t, *J* = 7.30 Hz, 1H), 7.55 (t, *J* = 7.80 Hz, 2H), 7.81 (d, *J* = 7.83 Hz, 2H), 7.98 (d, *J* = 8.32 Hz, 2H), 9.30 (s, 1H), 9.97 (s, 1H). ^13^C-NMR (150 MHz, DMSO-d6) δ 21.37, 119.67, 122.55, 128.13, 128.88, 129.04, 129.60, 130.16, 135.16, 139.06, 139.21, 153.20, 185.12.

##### 3-(4-Methoxyphenyl)-1-phenyl-1H-pyrazole-4-carbaldehyde (**4e**)

Appearance: White solid. Yield: 89%. ^1^H-NMR (600 MHz, DMSO-d6) δ 3.79 (s, 3H), 7.03 (d, *J* = 8.60 Hz, 2H), 7.39 (t, *J* = 7.43 Hz, 1H), 7.53 (t, *J* = 7.93 Hz, 2H), 7.86 (d, *J* = 8.56 Hz, 2H), 7.93 (d, *J* = 7.88 Hz, 2H), 9.21 (s, 1H), 9.93 (s, 1H). ^13^C-NMR (150 MHz, DMSO-d6) δ 55.65, 114.37, 119.59, 122.34, 124.03, 128.10, 130.16, 130.50, 135.51, 138.99, 152.82, 160.49, 185.12.

##### 3-(3-Methoxyphenyl)-1-phenyl-1H-pyrazole-4-carbaldehyde (**4f**)

Appearance: White solid. Yield: 82%. ^1^H-NMR (600 MHz, DMSO-d6) δ 3.82 (s, 3H), 7.04 (d, *J* = 8.30 Hz, 1H), 7.40 (t, *J* = 7.85 Hz, 2H), 7.49 (t, *J* = 7.65 Hz, 2H), 7.54 (t, *J* = 8.19 Hz, 2H), 7.98 (d, *J* = 8.36 Hz, 2H), 9.30 (s, 1H), 9.97 (s, 1H). ^13^C-NMR (150 MHz, DMSO-d6) δ 55.63, 114.46, 115.35, 119.70, 121.44, 122.68, 128.17, 130.11, 130.14, 132.97, 135.33, 139.02, 159.75, 185.08.

##### 3-(4-Bromophenyl)-1-phenyl-1H-pyrazole-4-carbaldehyde (**4g**)

Appearance: White solid. Yield: 76%. ^1^H-NMR (600 MHz, DMSO-d6) δ 7.43 (t, *J* = 7.35 Hz, 1H), 7.56 (t, *J* = 7.89 Hz, 1H), 7.70 (d, *J* = 8.28 Hz, 2H), 7.92 (d, *J* = 8.28 Hz, 2H), 7.98 (d, *J* = 8.22 Hz, 2H), 9.36 (s, 1H), 9.97 (s, 1H). ^13^C-NMR (150 MHz, DMSO-d6) δ 119.72, 122.63, 123.16, 128.31, 1230.22, 130.94, 131.09, 131.98, 136.21, 138.96, 151.65, 184.97.

##### 3-(3-Bromophenyl)-1-phenyl-1H-pyrazole-4-carbaldehyde (**4h**)

Appearance: White solid. Yield: 89%. ^1^H-NMR (600 MHz, DMSO-d6) δ 7.43–7.49 (m, 2H), 7.57 (t, *J* = 7.70 Hz, 2H), 7.67 (d, *J* = 8.17 HZ, 1H), 7.98 (d, *J* = 8.15 Hz, 3H), 8.18 (s, 1H), 9.37 (s, 1H), 9.97 (s, 1H). ^13^C-NMR (150 MHz, DMSO-d6) 119.76, 122.22, 122.68, 128.12, 128.35, 130.22, 131.14, 131.41, 132.41, 134.01, 136.50, 138.93, 151.07, 184.89.

##### 3-(4-Chlorophenyl)-1-phenyl-1H-pyrazole-4-carbaldehyde (**4i**)

Appearance: White solid. Yield: 82%. ^1^H-NMR (600 MHz, DMSO-d6) δ 7.42 (t, *J* = 7.40 Hz, 1H), 7.57 (t, *J* = 8.11 Hz, 4H), 7.98 (t, *J* = 7.67 Hz, 4H), 9.36 (s, 1H), 9.97 (s,1H). ^13^C-NMR (150 MHz, DMSO-d6) δ 119.72, 122.63, 128.30, 129.05, 130.21, 130.59, 130.83, 134.44, 136.21, 138.96, 151.59, 184.96.

##### 3-(3-Chlorophenyl)-1-phenyl-1H-pyrazole-4-carbaldehyde (**4j**)

Appearance: White solid. Yield: 89%. ^1^H-NMR (600 MHz, DMSO-d6) δ 7.43 (t, *J* = 7.47, 1H), 7.54 (t, *J* = 5.47, 2H), 7.57 (t, *J* = 8.10, 2H), 7.94–7.76 (m, 1H), 7.99 (d, *J* = 8.27, 2H), 8.05 (s, 1H), 9.38 (s, 1H), 9.98 (s, 1H). ^13^C-NMR (150 MHz, DMSO-d6) 119.76, 122.69, 127.74, 128.36, 128.60, 129.53, 130.23, 130.90, 133.71, 133.76, 136.49, 138.93, 151.18, 184.92.

##### 3-(4-Fluorophenyl)-1-phenyl-1H-pyrazole-4-carbaldehyde (**4k**)

Appearance: White solid. Yield: 84%. ^1^H-NMR (600 MHz, DMSO-d6) δ 7.32 (t, *J* = 8.94 Hz, 2H), 7.41 (t, *J* = 7.41 Hz, 1H), 7.55 (t, *J* = 8.40 Hz, 2H), 7.97 (d, *J* = 8.47 Hz, 2H), 8.00–8.02 (m, 2H), 9.33 (s, 1H), 9.96 (s, 1H). ^13^C-NMR (150 MHz, DMSO-d6) δ 115.84 (d, *J*_C-F_ = 22.45 Hz), 119.66, 122.50, 128.20, 130.17, 131.29 (d, *J*_C-F_ = 8.73 Hz), 135.98, 138.98, 151.86, 162.34, 163.97, 184.95.

##### 3-(3-Fluorophenyl)-1-phenyl-1H-pyrazole-4-carbaldehyde (**4l**)

Appearance: White solid. Yield: 85%. ^1^H-NMR (600 MHz, DMSO-d6) δ 7.31 (t, *J* = 8.30 Hz, 1H), 7.42 (t, *J* = 7.46 Hz, 1H), 7.53–7.58 (m, *J* = 7.67 Hz, 3H), 7.82 (d, *J* = 7.91 Hz, 2H), 7.98 (d, *J* = 7.91 Hz, 2H), 9.36 (s, 1H), 9.98 (s, 1H). ^13^C-NMR (150 MHz, DMSO-d6) 115.66 (d, *J*_C-F_ = 23.17 Hz), 116.40 (d, *J*_C-F_ = 21.81 Hz), 119.72, 122.69, 125.16 (d, *J*_C-F_ = 2.16 Hz), 128.31, 130.20, 130.99 (d, *J*_C-F_ = 8.64 Hz), 133.93 (d, *J*_C-F_ = 8.33 Hz), 136.30, 138.92, 151.10 (d, *J*_C-F_ = 2.10 Hz), 161.76, 163.37, 184.94.

##### 3-(2,4-Difluorophenyl)-1-phenyl-1H-pyrazole-4-carbaldehyde (**4m**)

Appearance: White solid. Yield: 96%. ^1^H-NMR (600 MHz, DMSO-d6) δ 7.42 (t, *J* = 7.41 Hz, 1H), 7.56 (t, *J* = 7.79 Hz, 3H), 7.87 (d, *J* = 2.05 Hz, 1H), 7.97 (d, *J* = 7.93 Hz, 2H), 8.06 (t, *J* = 8.52 Hz, 1H), 9.36 (s, 1H), 9.96 (s, 1H). ^13^C-NMR (150 MHz, DMSO-d6) 118.02, 118.10, 118.15, 118.22, 119.70, 122.57, 126.08, 126.10, 126.12, 128.34, 129.23, 130.20, 136.80, 138.87, 148.90, 148.98, 149.61, 149.69, 150.43, 150.52, 151.26, 151.34, 184.88. (multiple peaks observed due to fluorine).

##### 3-(4-Trifluoromethylphenyl)-1-phenyl-1H-pyrazole-4-carbaldehyde (**4n**)

Appearance: White solid. Yield: 84%. ^1^H-NMR (600 MHz, DMSO-d6) δ 7.43 (t, *J* = 7.50 Hz, 1H), 7.56 (t, *J* = 8.27 Hz, 2H), 7.85 (d, *J* = 8.30 Hz, 2H), 7.98 (d, *J* = 7.81 Hz, 2H), 8.17 (d, *J* = 8.17 Hz, 2H), 9.39 (s, 1H), 10.00 (s, 1H). ^13^C-NMR (150 MHz, DMSO-d6) δ 119.77, 122.86, 123.74, 125.55, 125.84, 125.87, 128.40, 129.65, 129.82, 129.86, 130.23, 135.74, 136.45, 138.93, 151.19, 184.92.

##### 3-(4-Nitrophenyl)-1-phenyl-1H-pyrazole-4-carbaldehyde (**4o**)

Appearance: White solid. Yield: 82%. ^1^H-NMR (600 MHz, DMSO-d6) δ 7.43 (t, *J* = 7.07 Hz, 1H), 7.56 (t, *J* = 7.66 Hz, 2H), 7.97 (d, *J* = 7.77 Hz, 2H), 8.23 (d, *J* = 8.55 Hz, 2H), 8.31 (d, *J* = 8.46 Hz, 2H), 9.40 (s, 1H), 9.99 (s, 1H). ^13^C-NMR (150 MHz, DMSO-d6) δ 119.75, 123.06, 124.08, 128.48, 130.12, 130.21, 137.00, 138.03, 138.82, 148.02, 150.25, 184.78.

#### 3.5.2. Analytical Data of Series 6

##### (*E*/Z)-N′-((1,3-Diphenyl-1H-pyrazol-4-yl)methylene)isonicotinohydrazide (**6a**)

Appearance: White solid. Yield: 91%. IR: 3051, 1652, 1507 cm^−1^. ^1^H-NMR (600 MHz, DMSO-d6) δ 7.36 (t, *J* = 7.15 Hz, 1H), 7.48–7.55 (m, 5H), 7.73 (d, *J* = 7.34 Hz, 2H), 7.83 (d, *J* = 4.53 Hz, 2H), 8.02 (d, *J* = 7.85 Hz, 2H), 8.59 (s, 1H), 8.78 (d, *J* = 4.23 Hz, 2H), 9.03 (s, 1H), 11.97 (s, 1H). ^13^C-NMR (*E* or *Z* configuration, 150 MHz, DMSO-d6) δ 117.09, 119.33, 121.96, 127.54, 127.72, 128.94, 129.14, 129.25, 130.09, 132.34, 139.45, 140.96, 142.75, 150.78, 152.57, 161.66. HRMS: (ESI^+^-MS, *m*/*z*) (M + Na) calcd for C_22_H_17_N_5_O 390.1331; found 390.1334.

##### (*E*/Z)-N′-((3-(4-Aminophenyl)-1-phenyl-1H-pyrazol-4-yl)methylene)isonicotinohydrazide (**6b**)

Appearance: White solid. Yield: 74%. IR: 3341, 3030, 1672 cm^−1^. ^1^H-NMR (600 MHz, DMSO-d6) δ 5.43 (s, 2H), 6.70 (d, *J* = 8.03 Hz, 2H), 7.34 (t, *J* = 7.15 Hz, 1H), 7.40 (d, *J* = 8.07 Hz, 2H), 7.51 (t, *J* = 7.54 Hz, 2H), 7.83 (d, *J* = 4.44 Hz, 2H), 7.99 (d, *J* = 7.82 Hz, 2H), 8.57 (s, 1H), 8.78 (d, *J* = 4.28 Hz, 2H), 8.94 (s, 1H), 11.92 (s, 1H). ^13^C-NMR (150 MHz, DMSO-d6) δ 114.21, 116.42, 119.10, 119.45, 121.97, 127.15, 127.21, 129.73, 130.02, 139.60, 141.08, 143.43, 149.86, 150.77, 153.46, 161.57. HRMS: (ESI^+^-MS, *m*/*z*) (M + Na) calcd for C_22_H_18_N_6_O (M): 405.1436; found 405.1440.

##### (*E*/Z)-N′-((3-(3-Aminophenyl)-1-phenyl-1H-pyrazol-4-yl)methylene)isonicotinohydrazide (**6c**)

Appearance: White solid. Yield: 74%. IR: 3046, 1650, 1596 cm^−1^. ^1^H-NMR (600 MHz, DMSO-d6) δ 6.69 (d, *J* = 7.54 Hz, 1H), 6.80 (d, *J* = 6.80 Hz, 1H), 6.96 (d, *J* = 7.02 Hz, 1H), 7.17 (t, *J* = 7.00 Hz, 1H), 7.36 (t, *J* = 8.50 Hz, 1H), 7.53 (t, *J* = 8.10 Hz, 2H), 7.84 (d, *J* = 5.45 Hz, 2H), 8.00 (d, *J* = 7.99 Hz, 2H), 8.58 (s, 1H), 8.79 (s, *J* = 6.54 Hz, 2H), 8.98 (s, 1H), 12.00 (s, 1H). ^13^C-NMR (150 MHz, DMSO-d6) δ 114.32, 114.80, 116.61, 117.02, 119.26, 122.01, 127.27, 127.40, 129.69, 130.06, 132.84, 139.55, 141.03, 143.13, 149.35, 150.74, 153.51, 161.66. HRMS: (ESI^+^-MS, *m*/*z*) (M + Na) calcd for C_22_H_18_N_6_O (M): 405.1444; found 405.1444.

##### (*E*/Z)-N′-((3-(4-Methylphenyl)-1-phenyl-1H-pyrazol-4-yl)methylene)isonicotinohydrazide (**6d**)

Appearance: White solid. Yield: 93%. IR: 3046, 1659, 1546 cm^−1^. ^1^H-NMR (600 MHz, DMSO-d6) δ 2.39 (s, 3H), 7.34 (d, *J* = 7.15 Hz, 1H), 7.38 (d, *J* = 7.31 Hz, 1H), 7.53 (t, *J* = 8.22 Hz, 2H), 7.62 (d, *J* = 8.14 Hz, 2H), 7.82 (d, *J* = 5.99 Hz, 2H), 8.01 (d, *J* = 8.17 Hz, 2H), 8.57 (s, 1H), 8.78 (d, *J* = 5.60 Hz, 2H), 9.01 (s, 1H), 11.94 (s, 1H). ^13^C-NMR (150 MHz, DMSO-d6) δ 21.33, 116.99, 119.30, 121.95, 127.48, 127.65, 128.83, 129.49, 129.80, 130.08, 138.64, 139.48, 140.97, 124.88, 150.78, 152.61, 161.62. HRMS: (ESI^+^-MS, *m*/*z*) (M + Na) calcd for C_23_H_19_N_5_O 404.1487; found 404.1493.

##### (*E*/Z)-N′-((3-(4-Methoxyphenyl)-1-phenyl-1H-pyrazol-4-yl)methylene)isonicotinohydrazide (**6e**)

Appearance: White solid. Yield: 95%. IR: 2935, 1649, 1506 cm^−1^. ^1^H-NMR (600 MHz, DMSO-d6) δ 3.82 (s, 3H), 7.09 (d, *J* = 8.42 Hz, 2H), 7.36 (t, *J* = 7.33 Hz, 1H), 7.52 (t, *J* = 7.81 Hz, 2H), 7.67 (d, *J* = 8.35 Hz, 2H), 7.82 (d, *J* = 4.82 Hz, 2H), 8.00 (d, *J* = 8.14 Hz, 2H), 8.56 (s, 1H), 8.78 (d, *J* = 4.78 Hz, 2H), 9.00 (s, 1H), 11.95 (s, 1H). ^13^C-NMR (150 MHz, DMSO-d6) δ 55.73, 114.65, 116.82, 119.25, 121.95, 124.71, 127.42, 127.65, 130.07, 130.26, 139.48, 140.98, 142.95, 150.78, 152.42, 160.11, 161.63. HRMS: (ESI^+^-MS, *m*/*z*) (M + Na) calcd for C_23_H_19_N_5_O_2_ 420.1436; found 420.1442.

##### (*E*/Z)-N′-((3-(3-Methoxyphenyl)-1-phenyl-1H-pyrazol-4-yl)methylene)isonicotinohydrazide (**6f**)

Appearance: White solid. Yield: 84%. IR: 3221, 1676, 1598 cm^−1^. ^1^H-NMR (600 MHz, DMSO-d6) δ 3.85 (s, 3H), 7.06 (d, *J* = 8.10 Hz, 1H), 7.27 (t, *J* = 6.74 Hz, 2H), 7.37 (t, *J* = 7.31 Hz, 1H), 7.44 (t, *J* = 7.97 Hz, 1H), 7.53 (t, *J* = 7.51 Hz, 2H), 7.82 (d, *J* = 4.50 Hz, 2H), 8.02 (d, *J* = 8.02 Hz, 2H), 8.60 (s, 1H), 8.78 (s, 2H), 9.02 (s, 1H), 11.98 (s, 1H). ^13^C-NMR (150 MHz, DMSO-d6) δ 55.65, 114.27, 114.77, 117.16, 119.38, 121.23, 121.98, 127.57, 127.77, 130.09, 130.39, 133.63, 139.43, 140.98, 142.71, 150.77, 152.39, 159.88, 161.71. HRMS: (ESI^+^-MS, *m*/*z*) (M + Na) calcd for C_23_H_19_N_5_O_2_ 420.1436; found 420.1441.

##### (*E*/Z)-N′-((3-(4-Bromophenyl)-1-phenyl-1H-pyrazol-4-yl)methylene)isonicotinohydrazide (**6g**)

Appearance: White solid. Yield: 82%. IR: 3022, 1656, 1498 cm^−1^. ^1^H-NMR (600 MHz, DMSO-d6) δ 3.85 (s, 3H), 7.06 (d, *J* = 8.10 Hz, 1H), 7.2 7.37 (t, *J* = 7.28 Hz, 1H), 7.53 (t, *J* = 7.65 Hz, 2H), 7.72 (t, *J* = 8.51 Hz, 4H), 7.82 (d, *J* = 4.55 Hz, 2H), 8.01 (d, *J* = 8.12 Hz, 2H), 8.55 (s, 1H), 8.78 (d, *J* = 4.90 Hz, 2H), 9.04 (s, 1H), 11.95 (s, 1H). ^13^C-NMR (150 MHz, DMSO-d6) δ 117.17, 119.37, 121.93, 122.60, 127.65, 128.38, 130.10, 130.98, 131.60, 132.13, 139.36, 140.92, 142.44, 150.80, 151.21, 161.64. HRMS: (ESI^+^-MS, *m*/*z*) (M + Na) calcd for C_22_H_16_BrN_5_O 469.0436; found 468.0444.

##### (*E*/Z)-N′-((3-(3-Bromophenyl)-1-phenyl-1H-pyrazol-4-yl)methylene)isonicotinohydrazide (**6h**)

Appearance: White solid. Yield: 81%. IR: 3052, 1649, 1536 cm^−1^. ^1^H-NMR (600 MHz, DMSO-d6) δ 7.36 (t, *J* = 7.35 Hz, 1H), 7.48 (t, *J* = 7.87 Hz, 1H), 7.52 (t, *J* = 7.86 Hz, 2H), 7.67 (d, *J* = 7.93 Hz, 1H), 7.76 (d, *J* = 7.68 Hz, 1H), 7.82 (d, *J* = 5.42 Hz, 2H), 7.95 (s, 1H), 8.00 (d, *J* = 8.12 Hz, 2H), 8.56 (s, 1H), 8.78 (d, *J* = 4.94 Hz, 2H), 9.02 (s, 1H), 12.00 (s, 1H). ^13^C-NMR (150 MHz, DMSO-d6) δ 17.30, 119.41, 121.98, 122.53, 127.68, 128.01, 128.30, 130.08, 131.17, 131.30, 131.91, 134.69, 139.32, 140.95, 142.21, 150.71, 150.77, 161.72. HRMS: (ESI^+^-MS, *m*/*z*) (M + Na) calcd for C_22_H_16_BrN_5_O 468.0436; found 468.0440.

##### (*E*/Z)-N′-((3-(4-Chlorophenyl)-1-phenyl-1H-pyrazol-4-yl)methylene)isonicotinohydrazide (**6i**)

Appearance: White solid. Yield: 80%. IR: 3023, 1656, 1499 cm^−1^. ^1^H-NMR (600 MHz, DMSO-d6) δ 7.37 (t, *J* = 7.47 Hz, 1H), 7.53 (t, *J* = 7.81 Hz, 2H), 7.58 (d, *J* = 8.26 Hz, 2H), 7.80–7.83 (m, 4H), 8.01 (d, 7.95, 2H), 8.56 (s, 1H), 8.78 (d, *J* = 5.04 Hz, 2H), 9.04 (s, 1H), 11.96 (s, 1H). ^13^C-NMR (150 MHz, DMSO-d6) δ 117.19, 119.36, 121.94, 127.63, 128.35, 129.20, 130.09, 130.70, 131.25, 133.94, 139.36, 140.93, 142.45, 150.79, 151.15, 161.65. HRMS: (ESI^+^-MS, *m*/*z*) (M + Na) calcd for C_22_H_16_ClN_5_O 424.0941; found 424.0956.

##### (*E*/Z)-N′-((3-(3-Chlorophenyl)-1-phenyl-1H-pyrazol-4-yl)methylene)isonicotinohydrazide (**6j**)

Appearance: White solid. Yield: 80%. IR: 3052, 1649, 1551 cm^−1^. ^1^H-NMR (600 MHz, DMSO-d6) δ 7.37 (t, *J* = 7.38 Hz, 1H), 7.53–7.56 (m, 4H), 7.73 (d, *J* = 6.73 Hz, 1H), 7.82–7.83 (m, 3H), 8.01 (d, *J* = 8.01 Hz, 2H), 8.57 (s, 1H), 8.78 (d, *J* = 5.46 Hz, 2H), 9.04 (s, 1H), 11.99 (s, 1H). ^13^C-NMR (150 MHz, DMSO-d6) δ 117.30, 119.41, 121.97, 127.63, 127.70, 128.34, 128.38, 129.02, 130.09, 131.08, 133.98, 134.45, 139.32, 140.96, 142.23, 149.95, 150.78, 161.72. HRMS: (ESI^+^-MS, *m*/*z*) (M + Na) calcd for C_22_H_16_ClN_5_O; found 424.0941; found 424.0948.

##### (*E*/Z)-N′-((3-(4-Fluorophenyl)-1-phenyl-1H-pyrazol-4-yl)methylene)isonicotinohydrazide (**6k**)

Appearance: White solid. Yield: 95%. IR: 3024, 1658, 1507 cm^−1^. ^1^H-NMR (600 MHz, DMSO-d6) δ 7.36 (t, *J* = 8.72 Hz, 3H), 7.53 (t, *J* = 7.82 Hz, 2H), 7.80–7.83 (m, 4H), 8.01 (d, *J* = 8.11 Hz, 2H), 8.55 (s, 1H), 8.78 (d, *J* = 5.31 Hz, 2H), 9.03 (s, 1H), 11.96 (s, 1H). ^13^C-NMR (150 MHz, DMSO-d6) δ 116.02, 116.24, 117.05, 119.33, 121.94, 127.57, 128.09, 128.85 (d, *J*_C-F_ = 3.01 Hz), 130.08, 131.08 (d, *J*_C-F_ = 8.31 Hz), 139.40, 140.95, 142.55, 150.79, 151.49, 161.64. HRMS: (ESI^+^-MS, *m*/*z*) (M + Na) calcd for C_22_H_16_FN_5_O 408.1237 found 408.1241.

##### (*E*/Z)-N′-((3-(3-Fluorophenyl)-1-phenyl-1H-pyrazol-4-yl)methylene)isonicotinohydrazide (**6l**)

Appearance: White solid. Yield: 95%. IR: 3064, 1647, 1538 cm^−1^. ^1^H-NMR (600 MHz, DMSO-d6) δ 7.31 (t, *J* = 7.55 Hz, 1H), 7.37 (t, *J* = 7.64 Hz, 1H), 7.53 (t, *J* = 7.99 Hz, 2H), 7.57–7.63 (m, 3H), 7.82 (t, *J* = 7.78 Hz, 2H), 7.80–7.83 (m, 4H), 8.02 (d, *J* = 8.10 Hz, 2H), 8.59 (s, 1H), 8.78 (d, *J* = 5.35 Hz, 2H), 9.04 (s, 1H), 11.99 (s, 1H). ^13^C-NMR (150 MHz, DMSO-d6) δ 115.46, 115.68, 115.84, 116.05, 117.27, 119.40, 121.96, 125.04, 127.68, 128.30, 130.09, 131.22, 131.31, 134.61, 134.70, 139.34, 140.96, 142.36, 150.96, 150.99, 161.50, 161.70, 163.92 (The additional carbon peaks observed due to carbon-fluorine coupling). HRMS: (ESI^+^-MS, *m*/*z*) (M + Na) calcd for C_22_H_16_FN_5_O 408.1237 found 408.1242.

##### (*E*/Z)-N′-((3-(2,4-Difluorophenyl)-1-phenyl-1H-pyrazol-4-yl)methylene)isonicotinohydrazide (**6m**)

Appearance: White solid. Yield: 85%. IR: 3024, 1661, 1508 cm^−1^. ^1^H-NMR (600 MHz, DMSO-d6) δ 7.36 (t, *J* = 7.28 Hz, 1H), 7.52 (t, *J* = 7.88 Hz, 2H), 7.56 (t, *J* = 9.86 Hz, 1H), 7.67 (s, 1H), 7.82 (d, *J* = 5.38 HZ, 2H), 7.91 (t, *J* = 9.73, 1H), 7.99 (d, *J* = 7.99 Hz, 2H), 8.55 (s, 1H), 8.78 (d, *J* = 5.39 Hz, 2H), 9.02 (s, 1H), 11.98 (s, 1H). ^13^C-NMR (150 MHz, DMSO-d6) δ 117.17, 117.94, 118.13, 118.33, 119.33, 121.95, 123.47, 125.87, 125.90, 125.94, 125.97, 127.67, 128.78, 130.06, 139.25, 140.89, 142.21, 150.01, 150.77, 161.73. (multiple peaks observed due fluorine) HRMS: (ESI^+^-MS, *m*/*z*) (M + Na) calcd for C_22_H_15_F_2_N_5_O 426.1142; found 426.1147.

##### (*E*/Z)-N′-((3-(4-Trifluoromethylphenyl)-1-phenyl-1H-pyrazol-4-yl)methylene)isonicotinohydrazide (**6n**)

Appearance: White solid. Yield: 95%. IR: 3423, 1648, 1596 cm^−1^. ^1^H-NMR (600 MHz, DMSO-d6) δ 7.39 (t, *J* = 7.36 Hz, 1H), 7.55 (t, *J* = 7.64 Hz, 2H), 7.82 (d, *J* = 7.99 Hz, 2H), 7.88 (d, *J* = 7.91 Hz, 2H), 8.02–8.04 (m, 4H), 8.58 (s, 1H), 8.78 (d, *J* = 5.20 Hz, 2H), 9.09 (s, 1H), 11.97 (s, 1H). ^13^C-NMR (*E* or *Z* configuration, 150 MHz, DMSO-d6) δ 117.50, 119.45, 121.93, 123.34, 125.97, 126.01, 126.05, 127.80, 128.68, 129.75, 130.13, 136.42, 139.32, 140.89, 142.26, 150.81, 161.68. HRMS: (ESI^+^-MS, *m*/*z*) (M + Na) calcd for C_23_H_16_F_3_N_5_O 458.1205; found 458.1212.

##### (*E*/Z)-N′-((3-(4-Nitrophenyl)-1-phenyl-1H-pyrazol-4-yl)methylene)isonicotinohydrazide (**6o**)

Appearance: White solid. Yield: 80%. IR: 3057, 1654, 1505 cm^−1^. ^1^H-NMR (600 MHz, DMSO-d6) δ 7.39 (t, *J* = 7.30, 1H), 7.53 (t, *J* = 7.91, 2H), 7.82 (d, *J* = 5.64, 2H), 8.01 (d, *J* = 8.00, 2H), 8.12 (d, *J* = 8.72, 2H), 8.33 (d, *J* = 8.75, 2H), 8.59 (s, 1H), 8.78 (d, *J* = 5.46, 2H), 9.07(s, 1H), 12.01 (s, 1H). ^13^C-NMR (150 MHz, DMSO-d6) δ 117.84, 119.45, 121.94, 124.21, 127.89, 129.34, 129.99, 130.11, 138.88, 139.20, 140.84, 141.99, 147.62, 149.76, 150.81, 161.74. HRMS: (ESI^+^-MS, *m*/*z*) (M + Na) calcd for C_22_H_16_N_6_O_3_ 435.1192; found 435.1188.

### 3.6. Biological Methods

#### 3.6.1. In Vitro Activity Against Mtb H37Rv

To evaluate the structure–activity relationship, a minimum inhibitory concentration (MIC) assay was conducted following established protocols [49]. Briefly, a 10 mL culture of *Mtb* H37Rv (ATCC 27294) was grown to an OD_600_ of 0.6–0.7 in standard Middlebrook 7H9 medium supplemented with glycerol, albumin-dextrose-catalase (ADC), and tween-80. The culture was then diluted in standard Middlebrook 7H9 medium to get an inoculum of ~5 × 10^6^ CFU/mL. A two-fold serial dilution of test compounds was prepared in a 96-well microtiter plate containing 50 μL of the growth medium. Finally, 50 μL of the diluted *Mtb* culture was added. Controls included media only, 5% DMSO, and INH. The microtiter plate was stored in a secondary container and incubated at 37 °C for 7 days with a humidifier to prevent evaporation of liquid. The Alamar Blue was added on 7th day, and the growth was recorded on the 8th day using both visual inspection and a fluorometer. The MIC was recorded as the compound concentration that inhibited the 90% bacterial growth. All experiments were conducted in duplicates with reproducible results.

#### 3.6.2. MDR-TB and XDR-TB Test

The effectiveness of the new compounds against TB was evaluated through experiments conducted on multidrug-resistant (MDR) and extensively drug-resistant (XDR) strains. The microplate alamar blue assay (MABA) was utilized for the testing [50,51] at Inkosi Albert Luthuli Hospital in Durban, South Africa, in the Department of Microbiology. To minimize evaporation of the medium, 100 µL of sterile deionized water was added to the outer wells of each 96-well plate. Subsequently, 50 μL of Middlebrook 7H9 broth was added to each well, and various diluted test compounds were prepared on the plate. The tested compounds had final concentrations ranging from 100 to 0.36 µg/mL. The experiment involved the use of drugs such as INH, RIF, and Moxifloxacin. Each well received a 50 μL inoculum supplement. The plates were sealed with parafilm and incubated at 37 °C for seven days. After the initial incubation period, 20 μL of resazurin dye solution was added to each well, followed by another overnight incubation. A blue color indicated no bacterial growth, while a pink color indicated bacterial growth. The minimum inhibitory concentration (MIC) was determined as the transition from blue to pink for the test candidates or compounds.

#### 3.6.3. Cytotoxicity Assay

Human THP-1 monocytes were grown in RPMI medium supplemented with 10% Fetal Bovine Serum (Sigma-Aldrich) and incubated at 37 °C with 5% CO_2_. Cells were differentiated with 20 ng/mL Phorbol Myristate Acetate (PMA) in a 96-well plate (2 × 10^4^ cells/well) for 48 h and exposed to decreasing concentrations (ranging from 25 μg/mL to 0.195 μg/mL) of the tested compounds. Following incubation (24 or 72 h), 10% *v*/*v* resazurin was added to each well and left to incubate for a few hours at 37 °C with 5% CO_2_. Data were measured using a fluorescent plate reader (excitation 540 nm, emission 590 nm). Results are the mean +/− SD of three independent experiments done in duplicate.

### 3.7. Physicochemical Studies

The in silico physicochemical properties were predicted using the online SwissADME server (http://www.swissadme.ch/index.php) (Accessed on 20 September 2025).

## 4. Conclusions

The anti-tubercular evaluation of INH–pyrazole molecular hybrids (**6a**–**o**) against *Mtb* H37RvMA (ATCC 27294) identified five potent compounds, namely **6a**, **6d**–**6f**, and **6n**, exhibiting an MIC of 0.9 µM. These compounds demonstrated approximately 4.3-fold higher potency compared to the standard drug, INH. However, a marked reduction in activity was observed against mutant strains of *Mtb*, including MDR and XDR strains, suggesting potential limitations in overcoming resistance mechanisms.

Furthermore, selected compounds (**6a**, **6d**–**f**, **6m**, and **6n**) exhibited greater than 80% cell viability, indicating low cytotoxicity at the tested concentrations. In silico ADME analysis revealed favorable pharmacokinetic profiles, with the compounds largely complying with established drug-likeness criteria.

Overall, these findings suggest that INH–pyrazole molecular hybrids represent promising scaffolds for antimycobacterial drug development. Nonetheless, further structural optimization is required to enhance their efficacy against resistant strains and to achieve improved pharmacological performance.

## Figures and Tables

**Table 1:** Antimycobacterial activity of the synthesized compounds and INH against *Mtb* H37RvMA (ATCC 27294), XDR and MDR.

S. No.	Product Code	Structure (a–o)	^a^ MIC (µM) (Visible)	Resazurin ^a^ MIC (µM) 7 Days	Resazurin ^a^ MIC (µM) 14 Days	MIC XDR (µM)	MIC MDR (µM)
1	**4a**	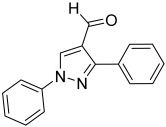	31.25	31.25	62.5	>403	^b^ ND
2	**4b**	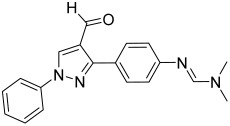	>62.5	>62.5	>62.5	ND	ND
3	**4c**	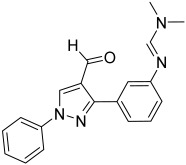	>62.5	>62.5	>62.5	ND	ND
4	**4d**	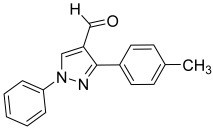	>62.5	>62.5	>62.5	ND	ND
5	**4e**	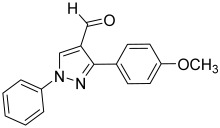	31.25	31.25	62.5	ND	ND
6	**4f**	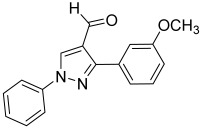	62.5	62.5	62.5	ND	ND
7	**4g**	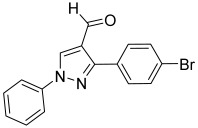	>62.5	>62.5	>62.5	ND	ND
8	**4h**	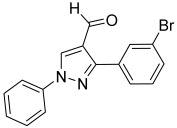	>62.5	>62.5	>62.5	ND	76
9	**4i**	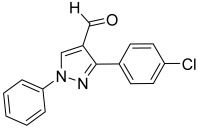	>62.5	>62.5	>62.5	ND	ND
10	**4j**	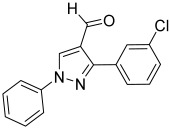	>62.5	>62.5	>62.5	ND	ND
11	**4k**	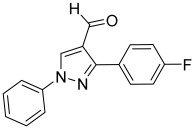	>62.5	>62.5	>62.5	ND	ND
12	**4l**	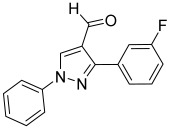	>62.5	>62.5	>62.5	ND	ND
13	**4m**	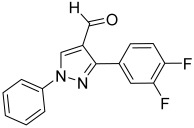	62.5	62.5	62.5	ND	ND
14	**4n**	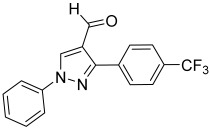	>62.5	62.5	>62.5	ND	ND
15	**4o**	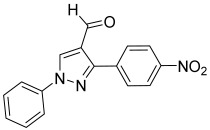	ND	ND	ND	>341	ND
16	**6a**	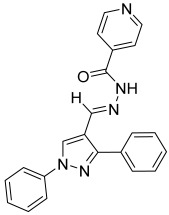	0.9	0.9	0.9	ND	ND
17	**6b**	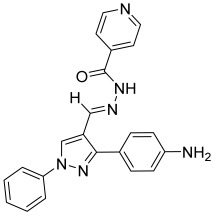	1.95	1.95	1.95	ND	ND
18	**6c**	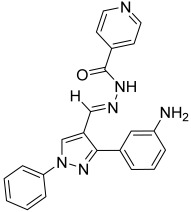	1.95	1.95	1.95	ND	ND
19	**6d**	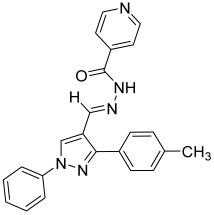	0.9	0.9	0.9	ND	>262
20	**6e**	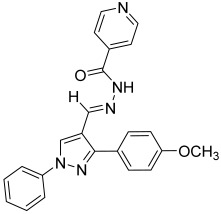	0.9	0.9	0.9	ND	125
21	**6f**	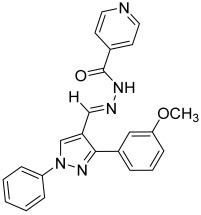	0.9	0.9	0.9	ND	ND
22	**6g**	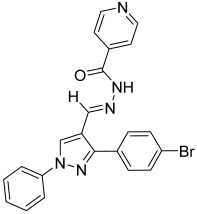	1.95	1.95	1.95	ND	ND
23	**6h**	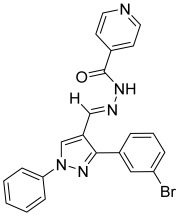	1.95	1.95	1.95	ND	112
24	**6i**	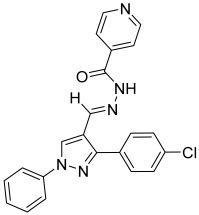	1.95	1.95	1.95	ND	ND
25	**6j**	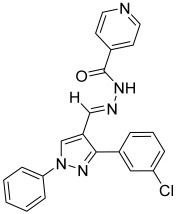	3.9	3.9	3.9	ND	>249
26	**6k**	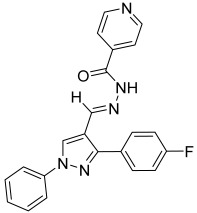	1.95	1.95	1.95	ND	>259
27	**6l**	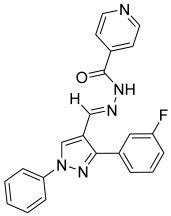	1.95	1.95	1.95	ND	ND
28	**6m**	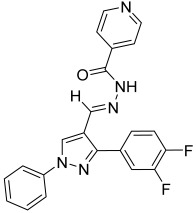	0.9	0.9	0.9	ND	ND
29	**6n**	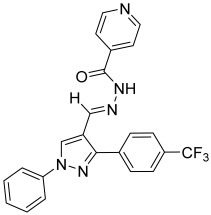	3.9	3.9	3.9	ND	ND
30	**6o**	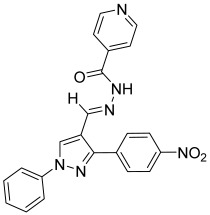	3.9	3.9	3.9	121	ND
**INH**			3.9	3.9	3.9	91.0	91.0
Moxifloxacin						31	7.78
Rifampicin						15.2	15.2

^a^ MIC refers to MIC_90_, defined as the minimum concentration of a compound required to inhibit 90% of the growth of *Mtb*. ^b^ ND: not determined.

**Table 2:** Minimal Inhibitory Concentration (MIC) of the selected most potent compounds against three INH-resistant strains compared to the WT strain and their Selectivity Index (SI).

Code	MIC (µM) *Mtb* H37RvMA (ATCC 27294)	MIC (µg/mL)	CC_50_ ^a^ (Day 1) (µg/mL)	SI ^b^ (Day 1)	CC_50_ ^a^ (Day 3) (µg/mL)	SI ^b^ (Day 3)	Mutant Strains		
							H37Rv—INH-R (Mutation: *KatG*_W198) MIC (µM)	H37Rv—INH-R-RaI (Mutation: *KatG*(t198a) MIC (µM)	H37Rv—INH-R-RM30 (Mutation: c15t_InhA) MIC (µM)
**6a**	3.9	1.4	ND	ND	ND	ND	>62.5	15.6	31.3
**6d**	3.9	1.5	25.6	17.0	20.9	13.9	>62.5	15.6	15.6
**6e**	3.9	1.5	ND	ND	ND	ND	>62.5	31.3	15.6
**6f**	3.9	1.5	27.8	18.5	22.1	14.7	>62.5	15.6	31.3
**6n**	3.9	1.7	21.2	12.5	19.0	11.2	>62.5	>62.5	>62.5
**INH**	1.95	0.3	ND	ND	ND	ND	>62.5	7.8–3.9	7.8

^a^ CC_50_ (µg/mL) = represents the concentration that kills 50% of the cells, i.e., the concentration that is toxic to the cell. ^b^ SI = CC_50_ in THP-1 cells (µg/mL)/MIC (µg/mL). Selectivity index is defined as the ratio of cytotoxicity on cells to inhibitory activities on bacteria. ND not determined.

**Table 3:** The physicochemical properties of compounds **6a**, **6d**–**6f**, and **6n**.

Compound	Physicochemical Properties					
	M.F	M.W	HBA	HBD	^rot^ BOND	^t^ PSA (A^2^)
**6a**	C_22_H_17_N_5_O	367.40	4	1	6	72.17
**6d**	C_23_H_19_N_5_O	381.43	4	1	6	72.17
**6e**	C_23_H_19_N_5_O_2_	397.43	5	1	7	81.40
**6f**	C_23_H_19_N_5_O_2_	397.43	5	1	7	81.40
**6n**	C_22_H_15_F_2_N_5_O	403.38	6	1	6	72.17
Standard range	-	<500	0–20	0–6	0–11	0–140

M.F = Molecular formula; M.W = molecular weight; HBD = no. of hydrogen bond donor; HBA = no. of hydrogen bond acceptor; ^rot^ BOND = no. of rotatable bond; ^t^ PSA = total polar surface area.

## Data Availability

The original contributions presented in this study are included in the article/Supplementary Materials. Further inquiries can be directed to the corresponding authors.

## Supplementary Materials

The following supporting information can be downloaded at: https://www.mdpi.com/article/10.3390/ijms27104385/s1.
