# Supplementary material for: Development of Isoniazid–Pyrazole Hybrids as Potential Antitubercular Agents
Source: Int J Mol Sci. 2026 May 14;27(10):4385. doi: 10.3390/ijms27104385 (PMC13207674; doi:10.3390/ijms27104385)
Supplement: Supplementary file 1 [file ijms-27-04385-s001.zip › ijms-4291321-supplementary.pdf]

Supporting Information

Development of Isoniazid-Pyrazole Hybrids as Potential Antitubercular Agents

Mukanda Gedeon Kadima[a], Vinayak Singh[b], Gobind Kumar,[c] Sahil Mishra,[c] Pule Seboletswe,[c] Ankit[c], Afsana Kajee,[d], Françoise Roquet-Banères, [e] Laurent Kremer, [e,f] Rajshekhar Karpoormath,[a*] Parvesh Singh[c*]

[a]*Discipline of Pharmaceutical Sciences, Westville Campus, University of KwaZulu-Natal, Durban 4000, South Africa.*
[b]*Institute of Infectious Disease and Molecular Medicine (IDM), Division of Medical Microbiology, Department of Pathology, Faculty of Health Sciences, University of Cape Town, Rondebosch 7701, Cape Town, South Africa.*
[c]*Discipline of Chemistry, University of KwaZulu-Natal, P/Bag X54001, Westville, Durban 4000, South Africa*
[d]*Department of Microbiology, National Health Laboratory Services (NHLS), Inkosi Albert Luthuli Central Hospital, Durban, South Africa*
[e]*Centre National de la Recherche Scientifique UMR 9004, Institut de Recherche en Infectiologie de Montpellier (IRIM), Université de Montpellier, 1919 route de Mende, 34293, Montpellier, France.*
[f]*INSERM, IRIM, 34293 Montpellier, France.*

*Correspondence: singhp4@ukzn.ac.za, karpoormath@ukzn.ac.za

1. **Spectral data of series 4**

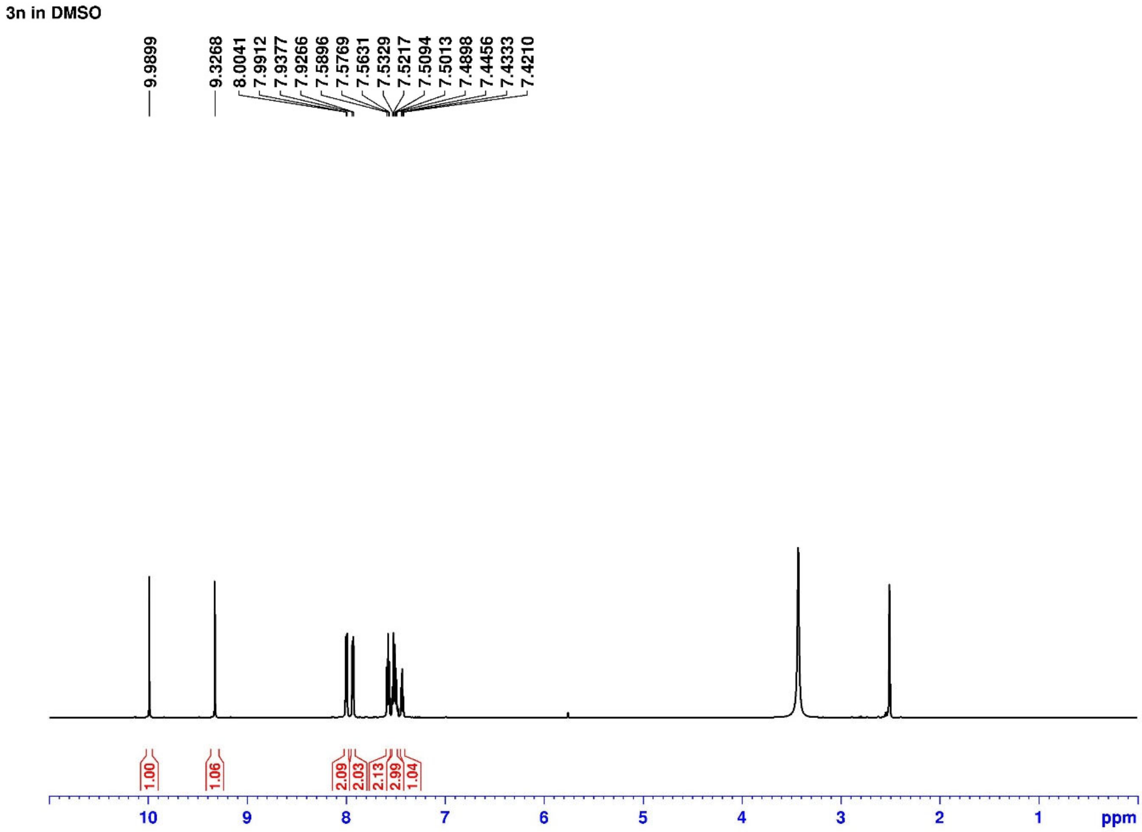

^{1}HNMR spectra of **4a**.

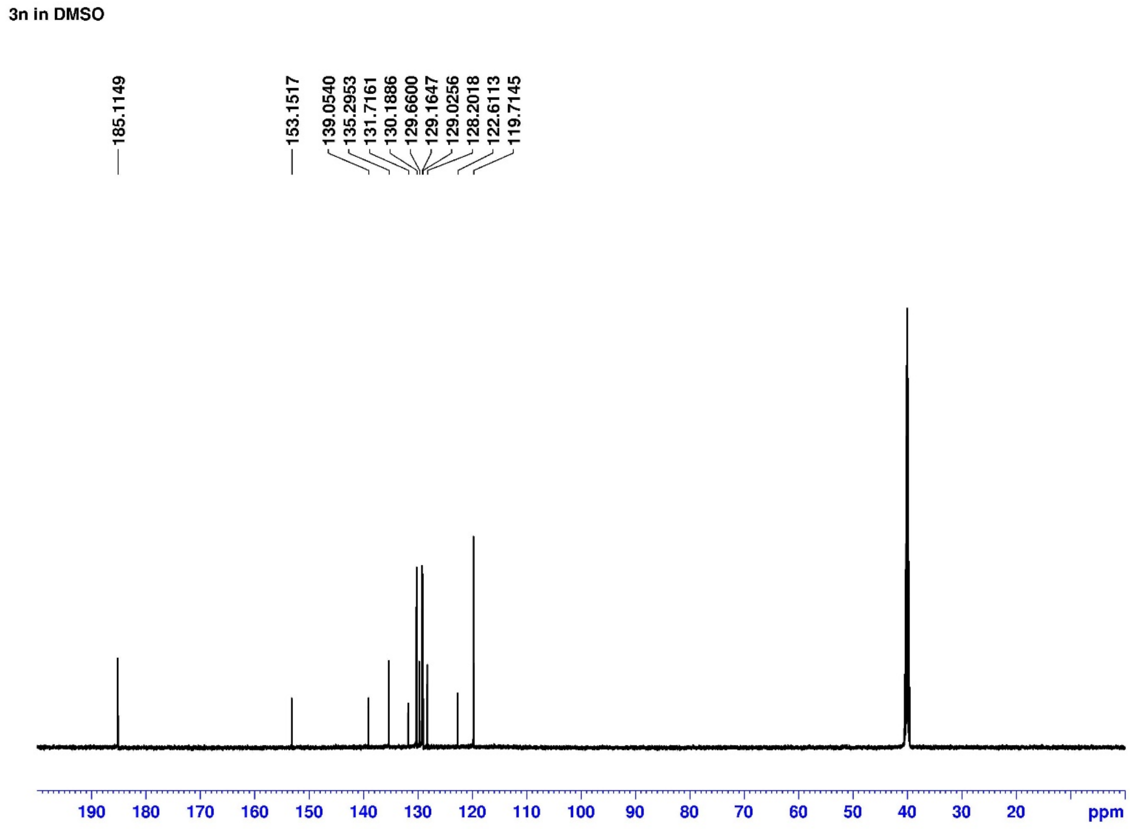

^{13}CNMR spectra of **4a**.

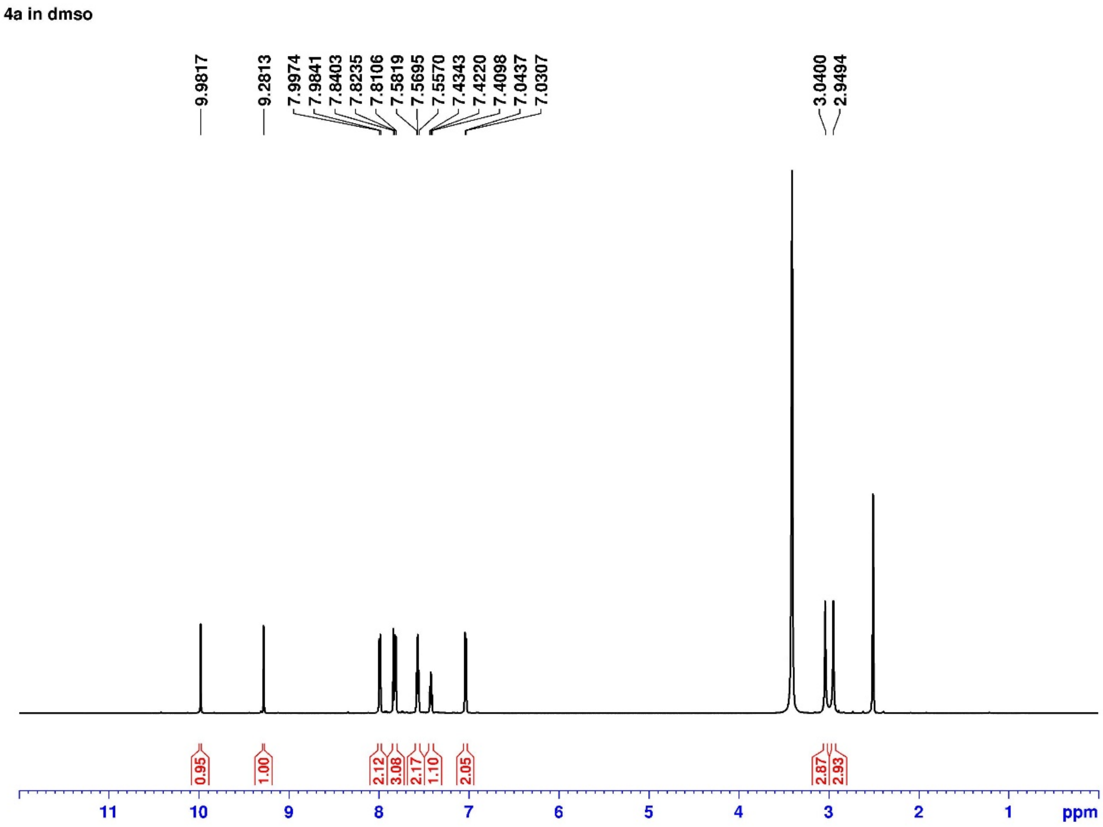

^{1}HNMR spectra of **4b**.

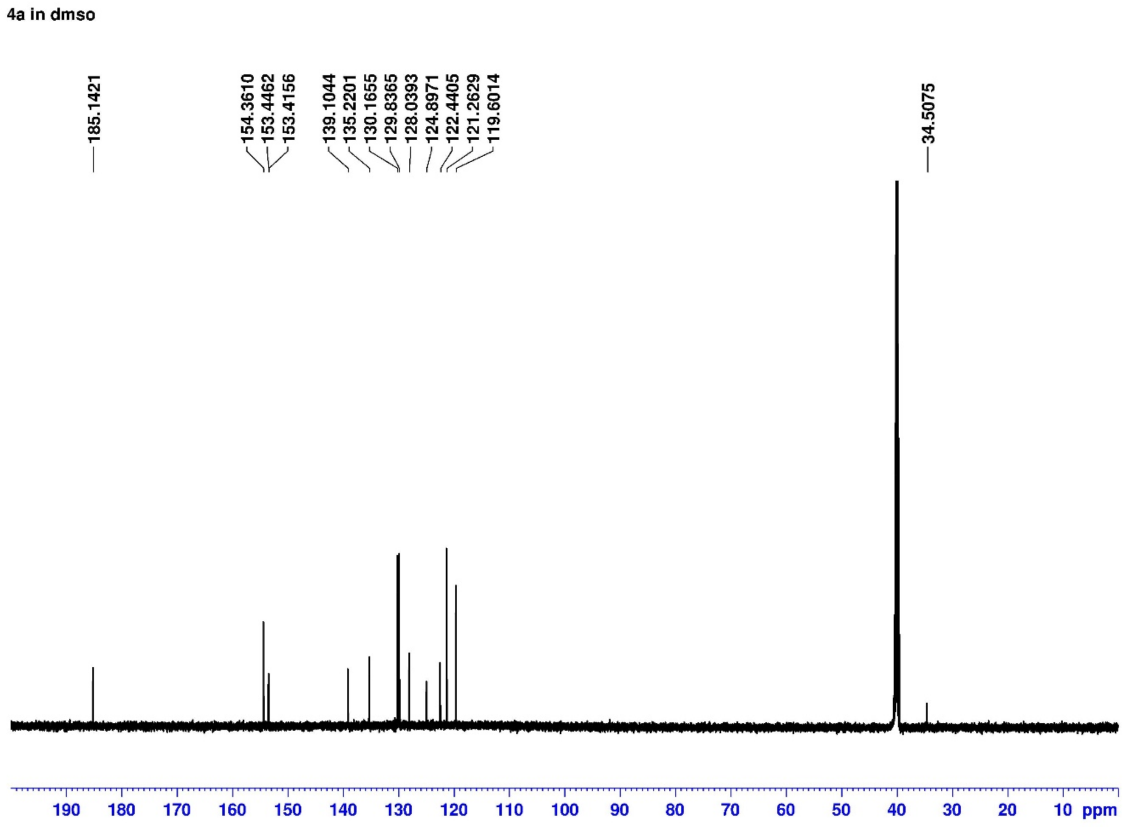

^{13}CNMR spectra of **4b**.

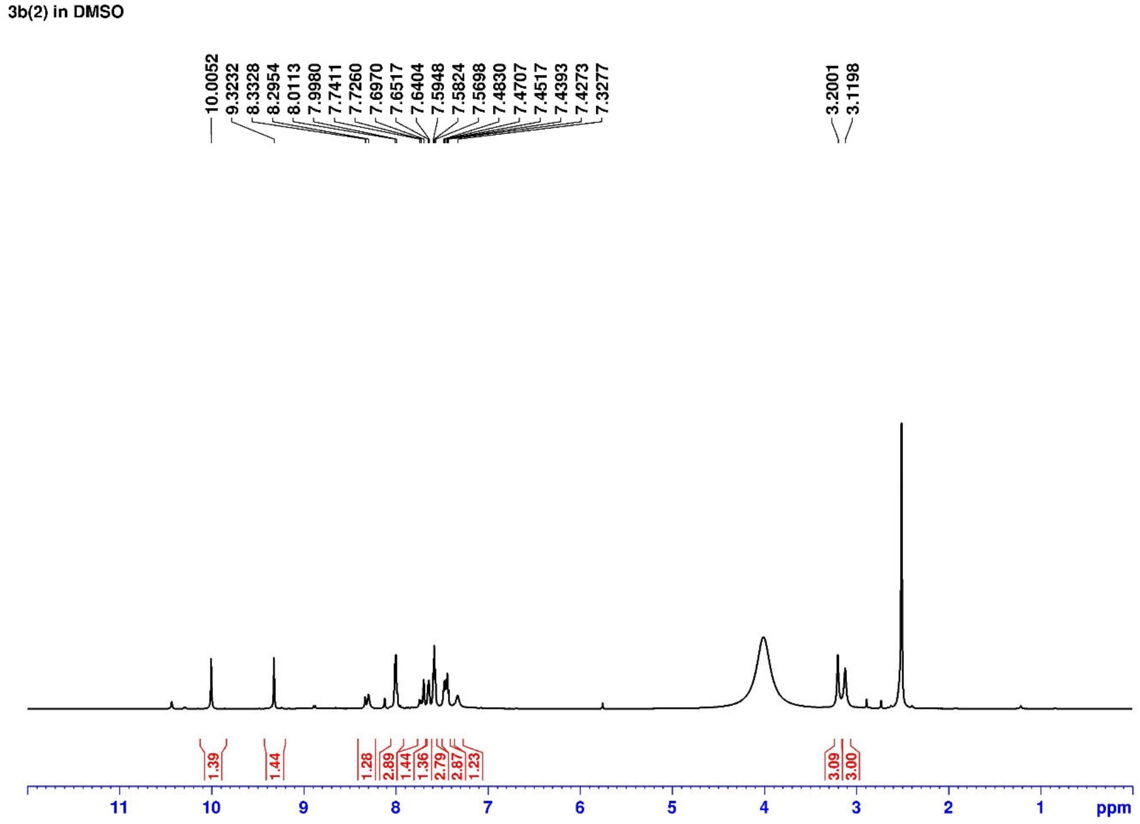

^{1}HNMR spectra of **4c**.

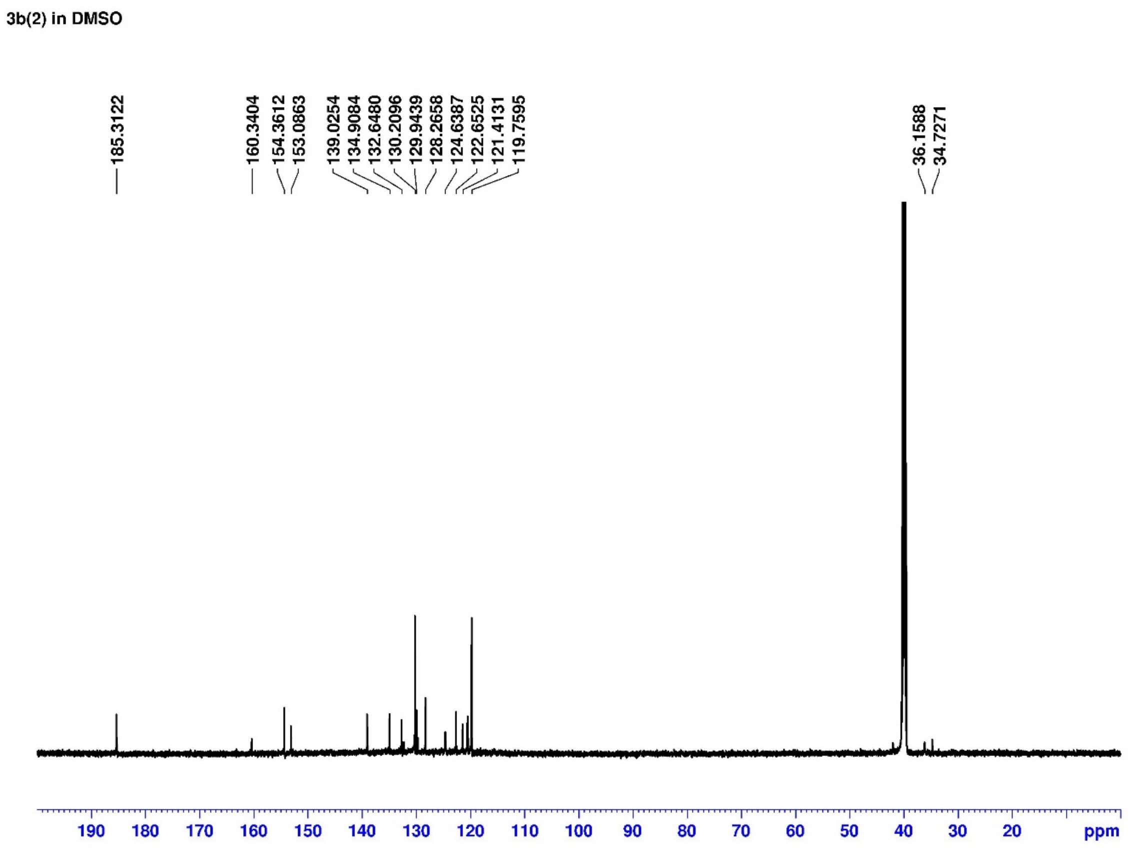

^{13}CNMR spectra of **4c**.

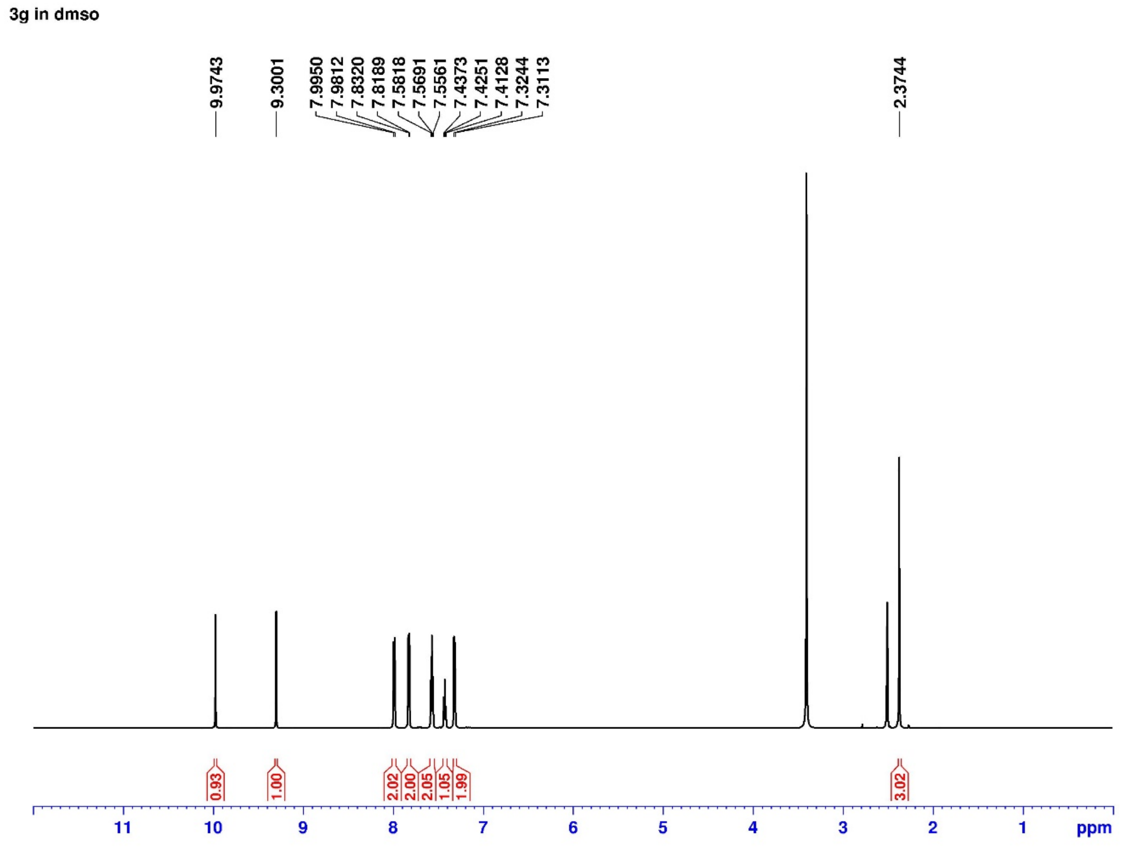

^{1}HNMR spectra of **4d**.

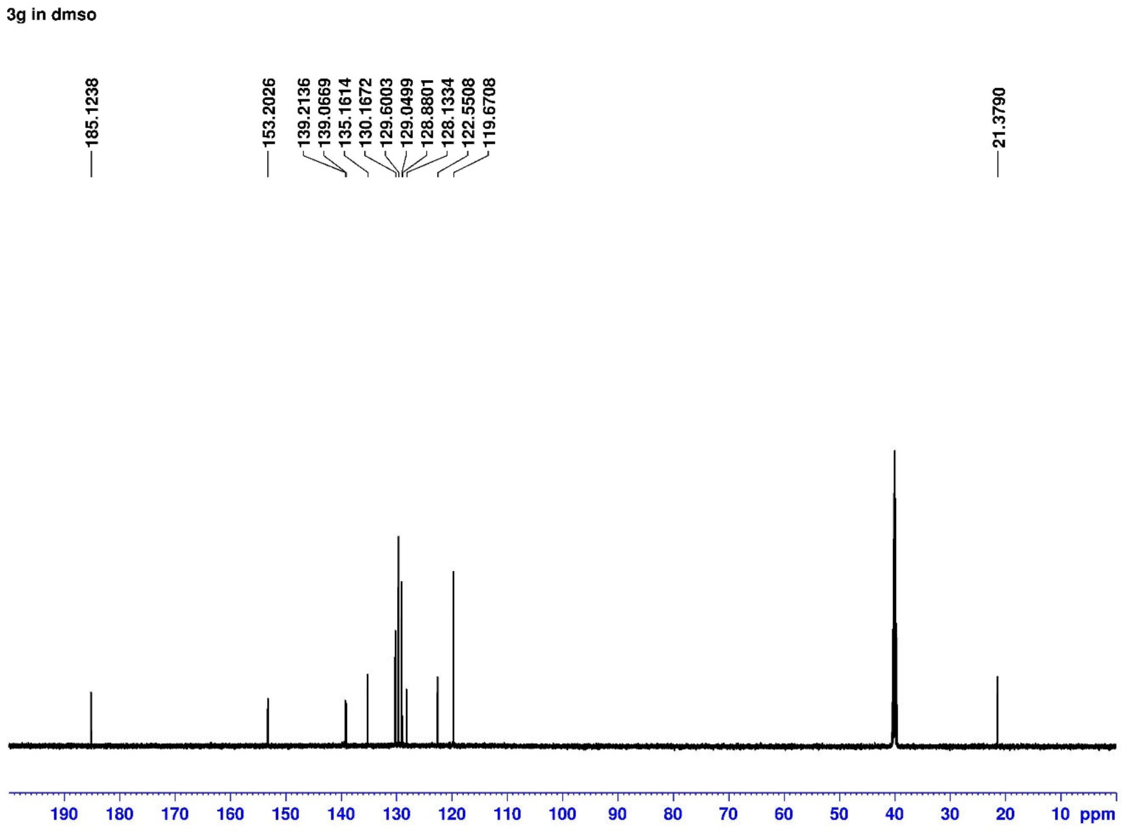

^{13}CNMR spectra of **4d**.

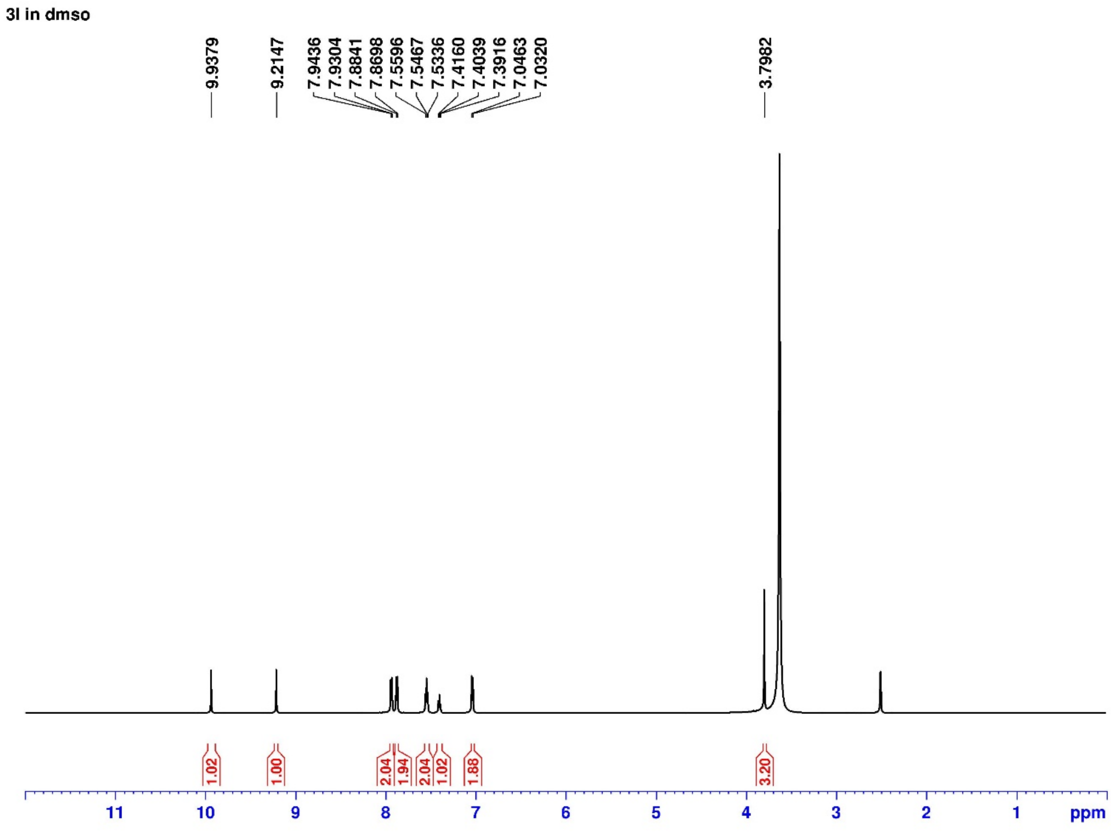

^{1}HNMR spectra of **4e**.

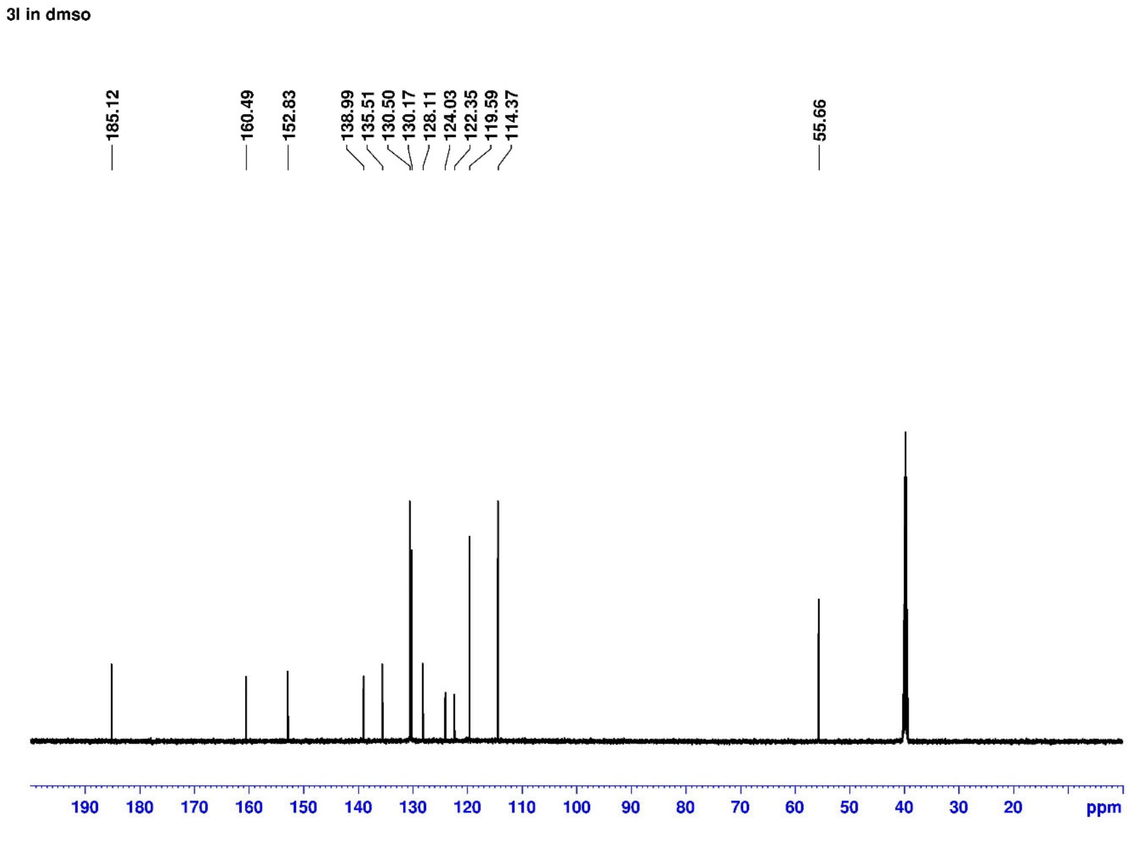

^{13}CNMR spectra of **4e**.

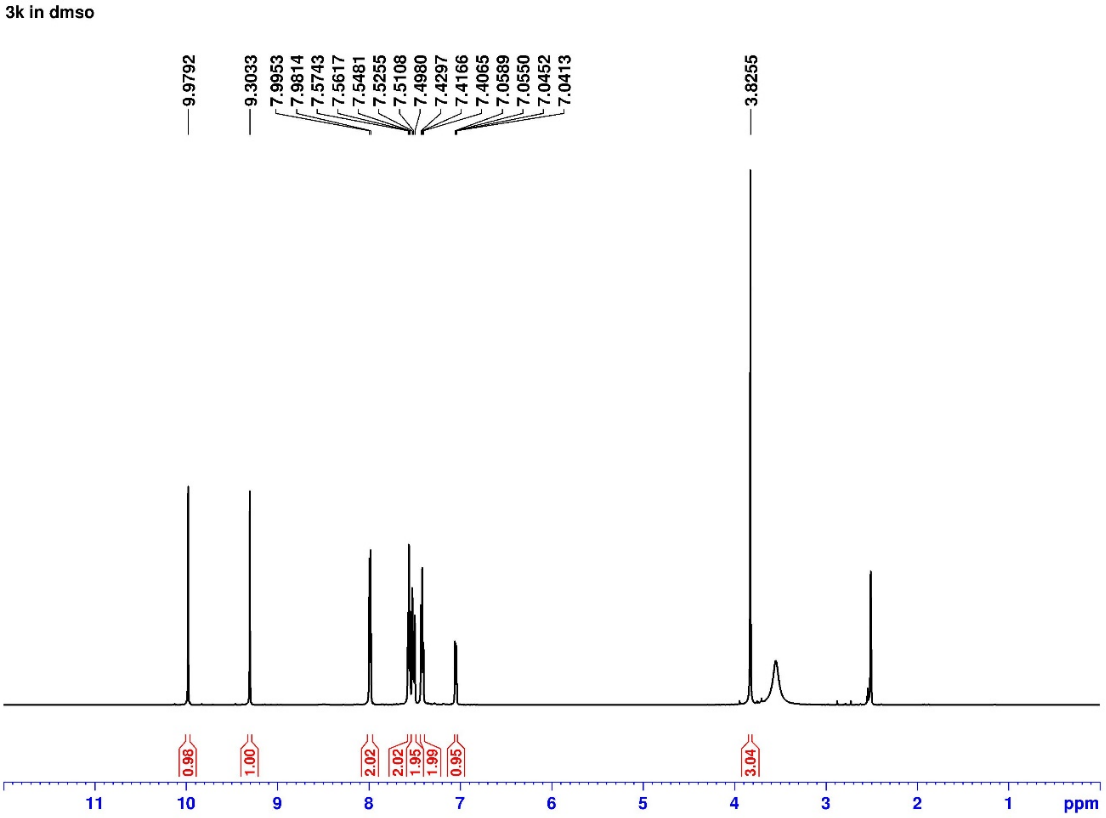

^{1}HNMR spectra of **4f**.

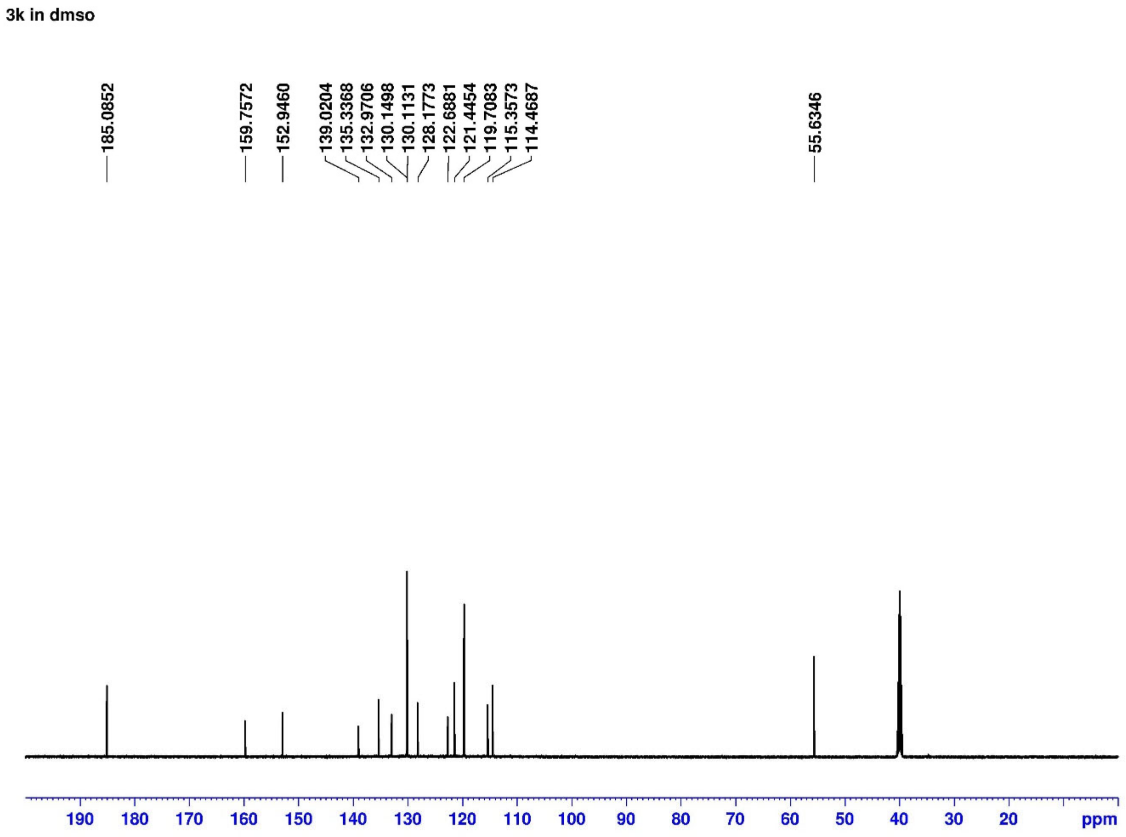

^{13}CNMR spectra of **4f**.

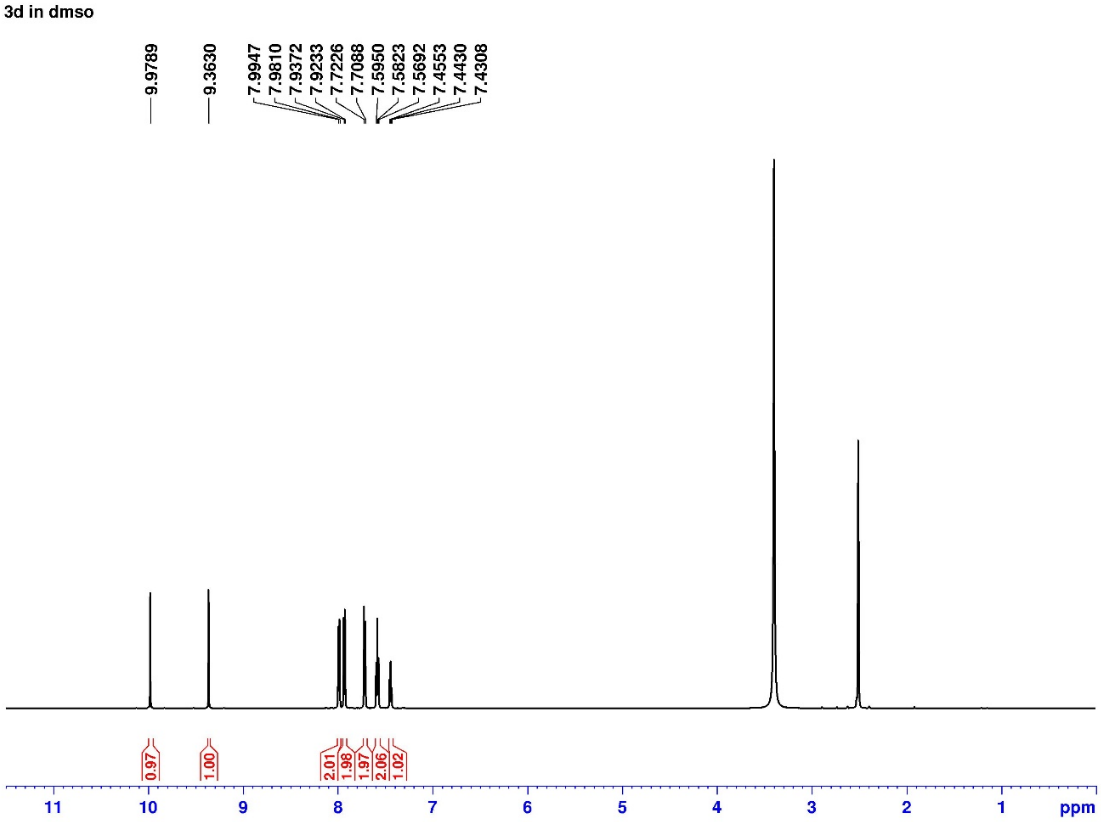

^{1}HNMR spectra of **4g**.

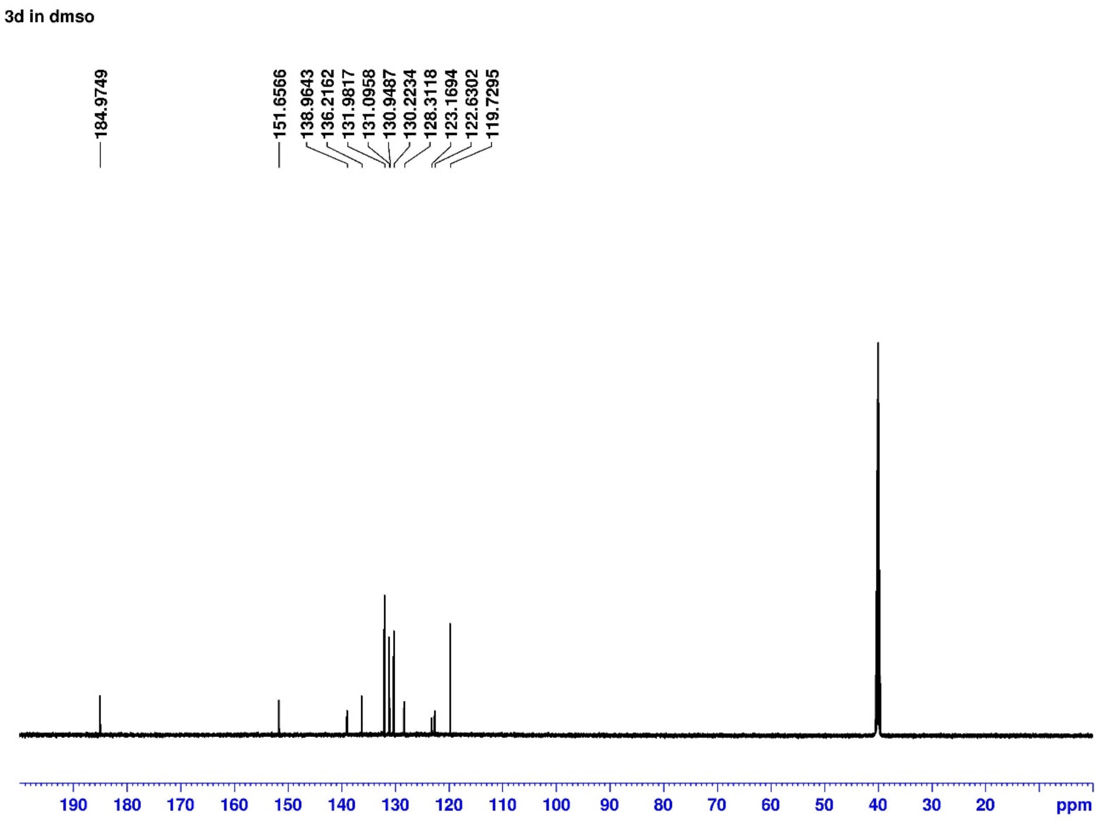

^{13}CNMR spectra of **4g**.

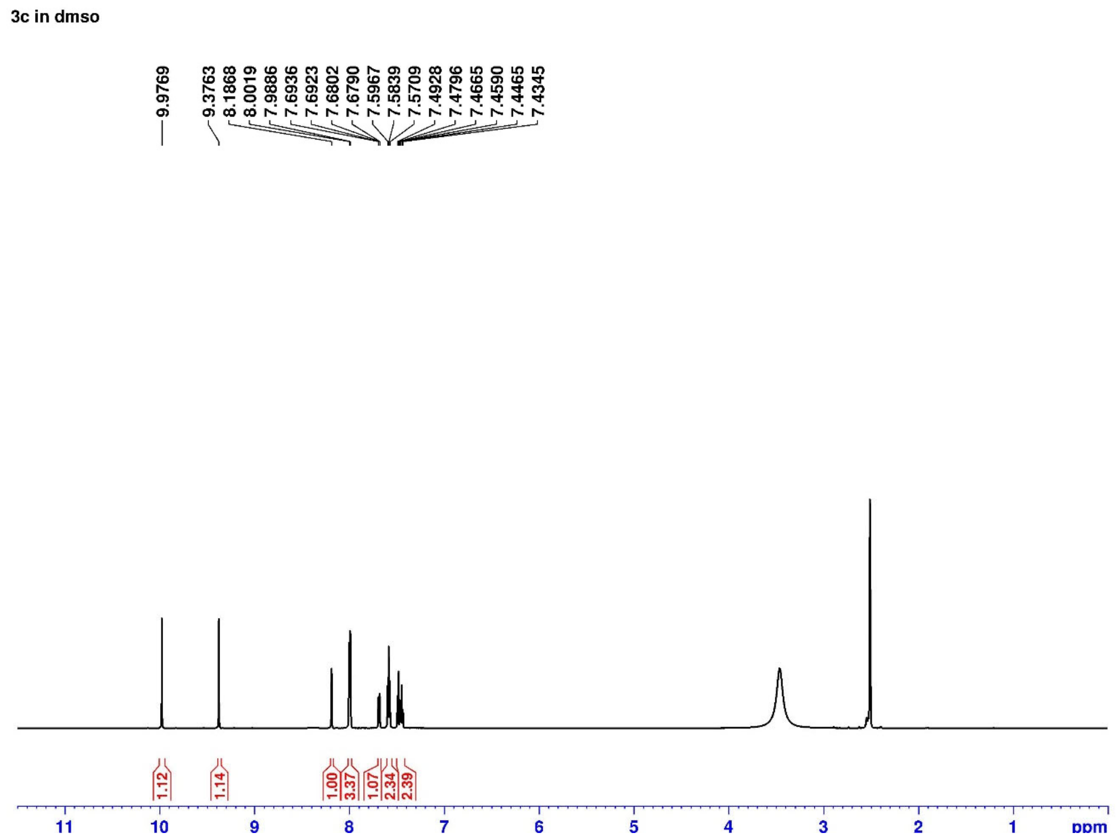

^{1}HNMR spectra of **4h**.

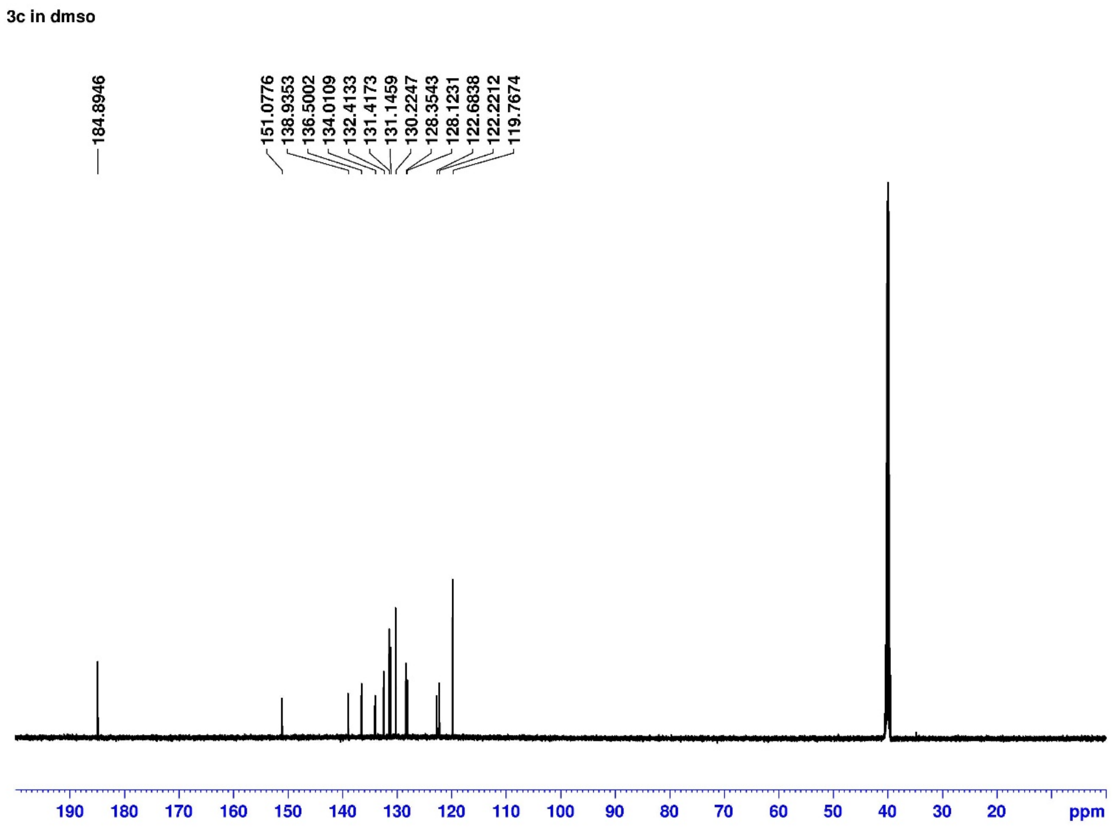

^{13}CNMR spectra of **4h**.

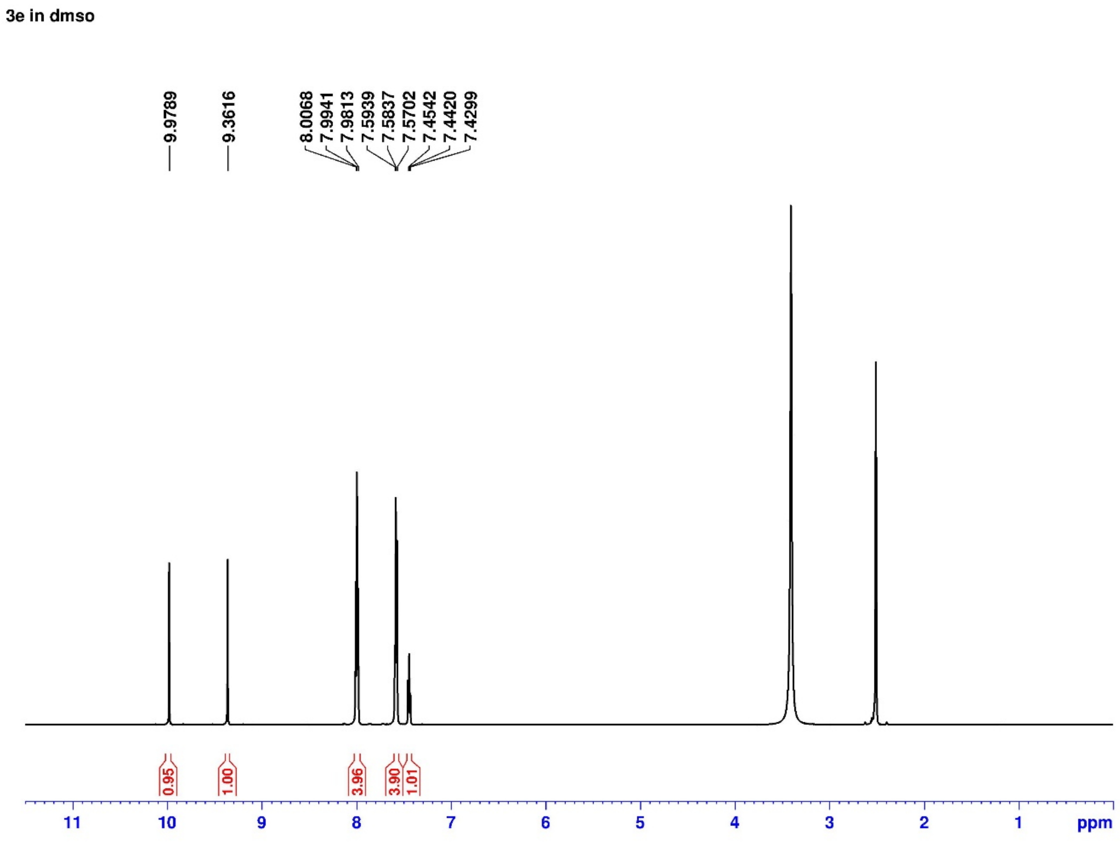

^{1}HNMR spectra of **4i**.

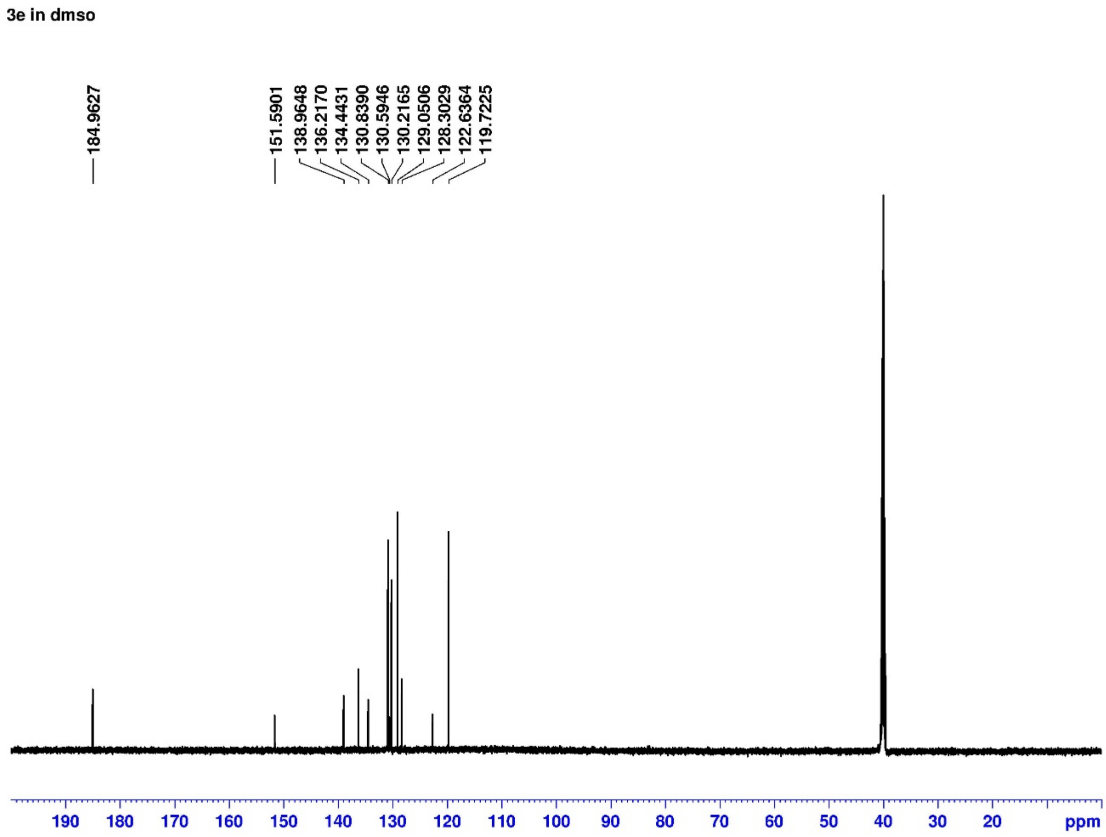

^{13}CNMR spectra of **4i**.

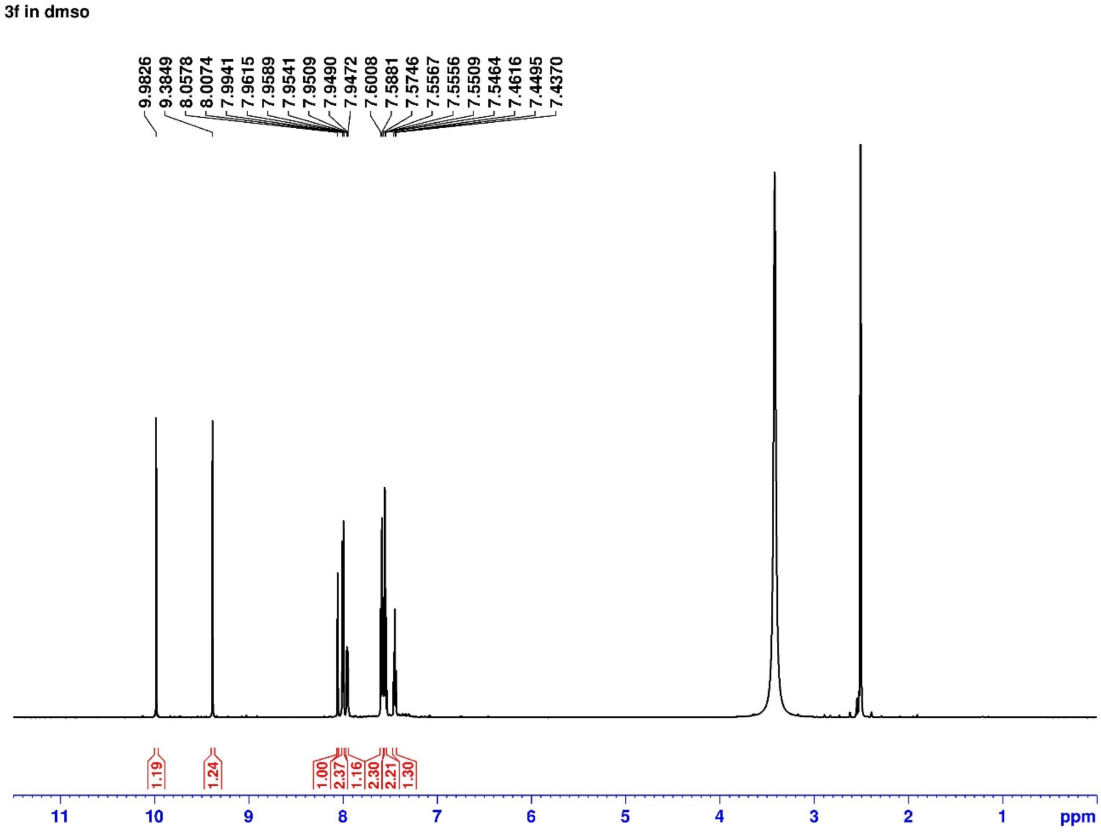

^{1}HNMR spectra of **4j**.

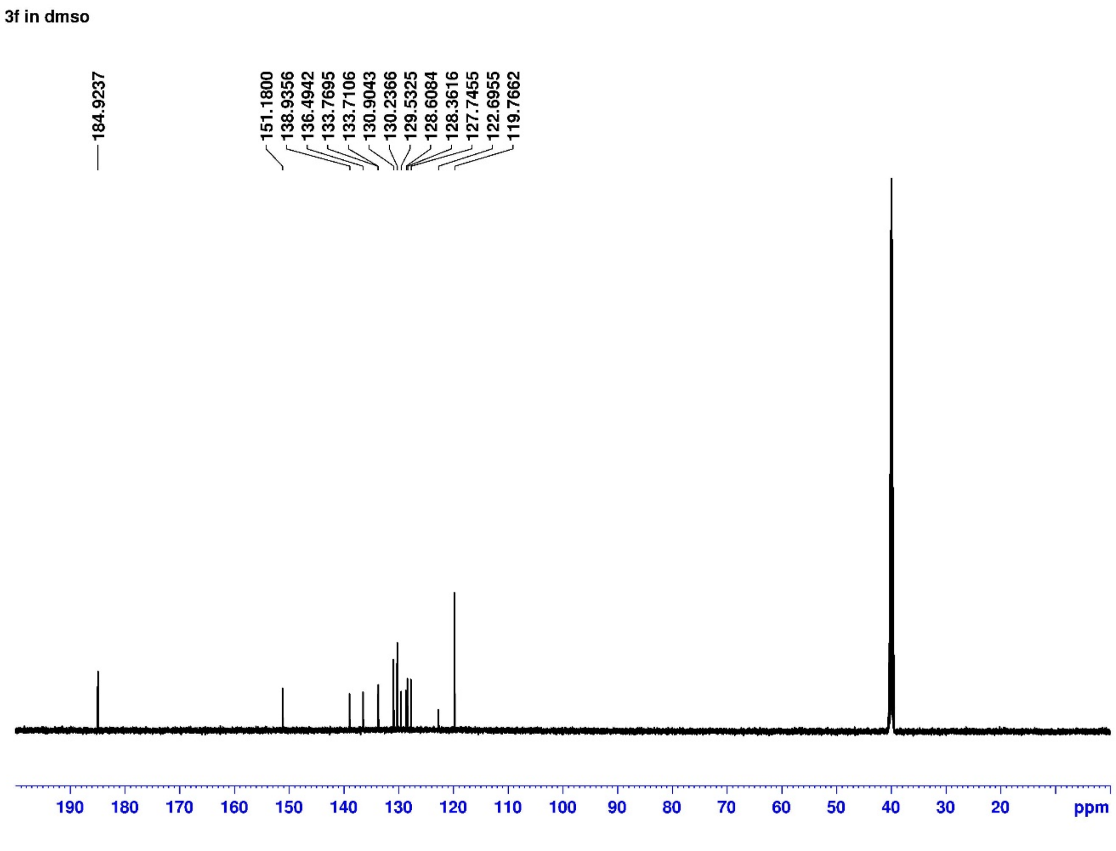

^{13}CNMR spectra of **4j**.

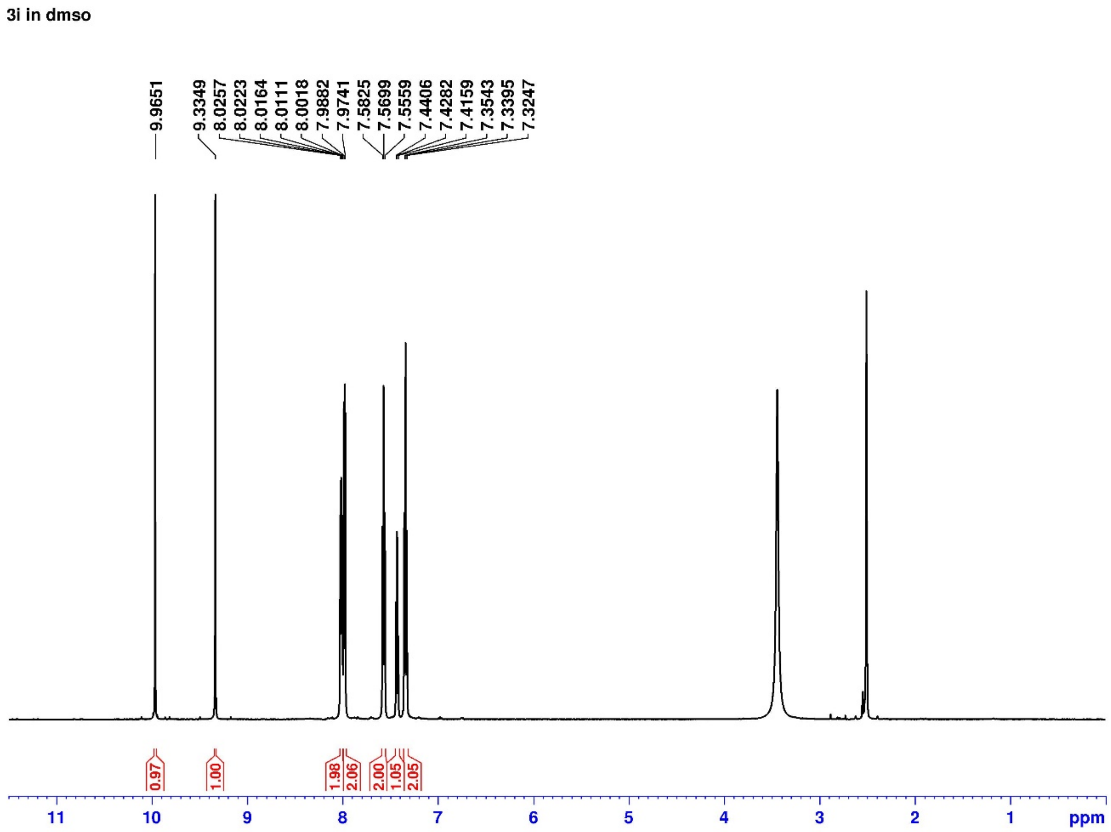

^{1}HNMR spectra of **4k**.

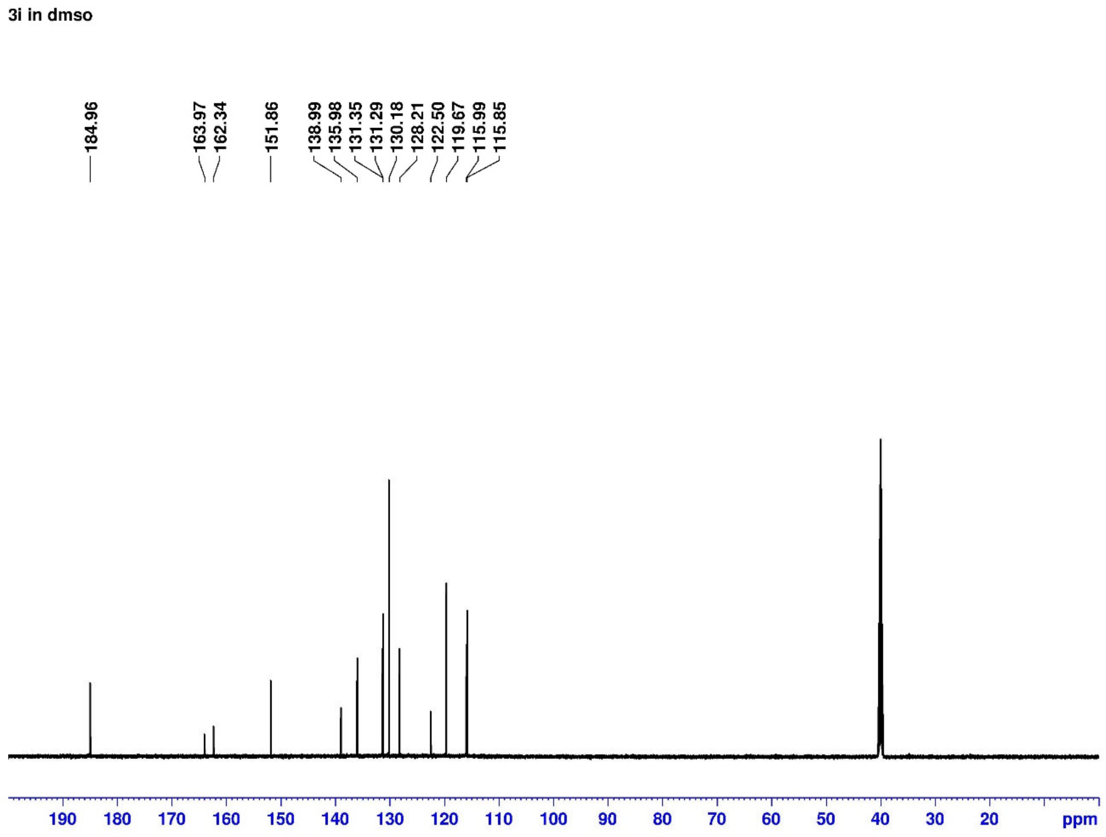

^{13}CNMR spectra of **4k**.

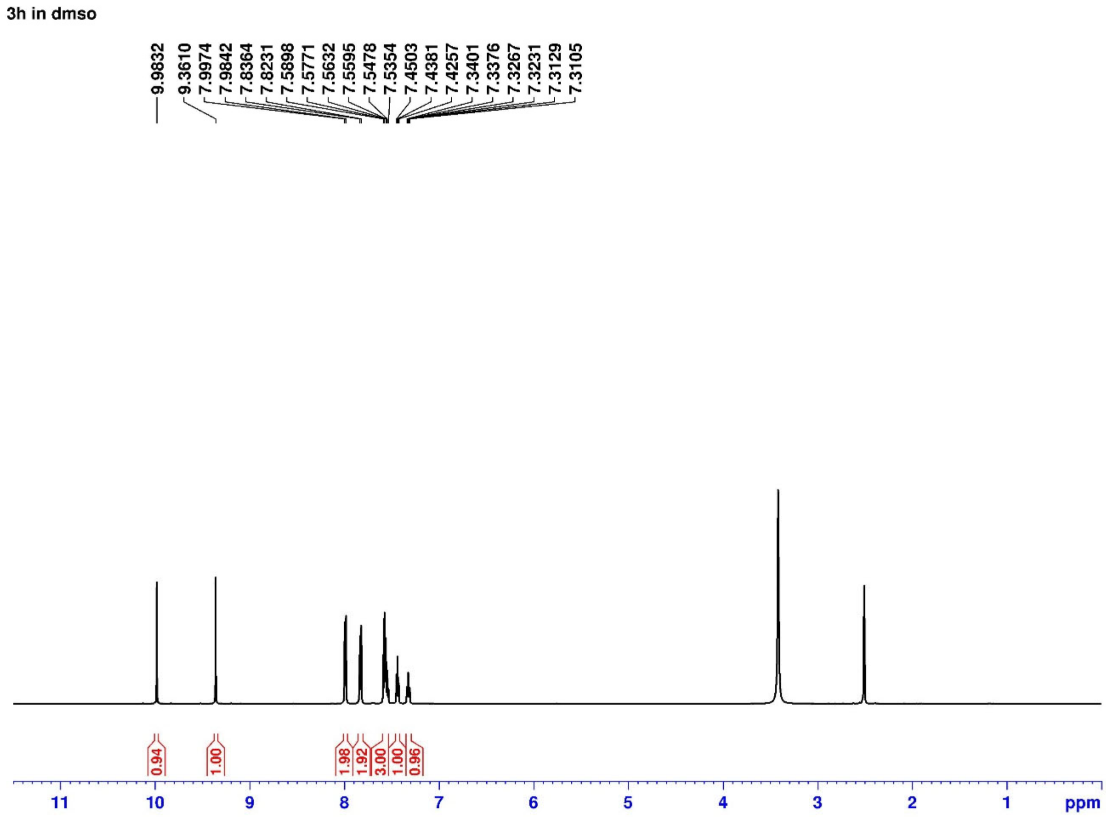

^{1}HNMR spectra of **4l**.

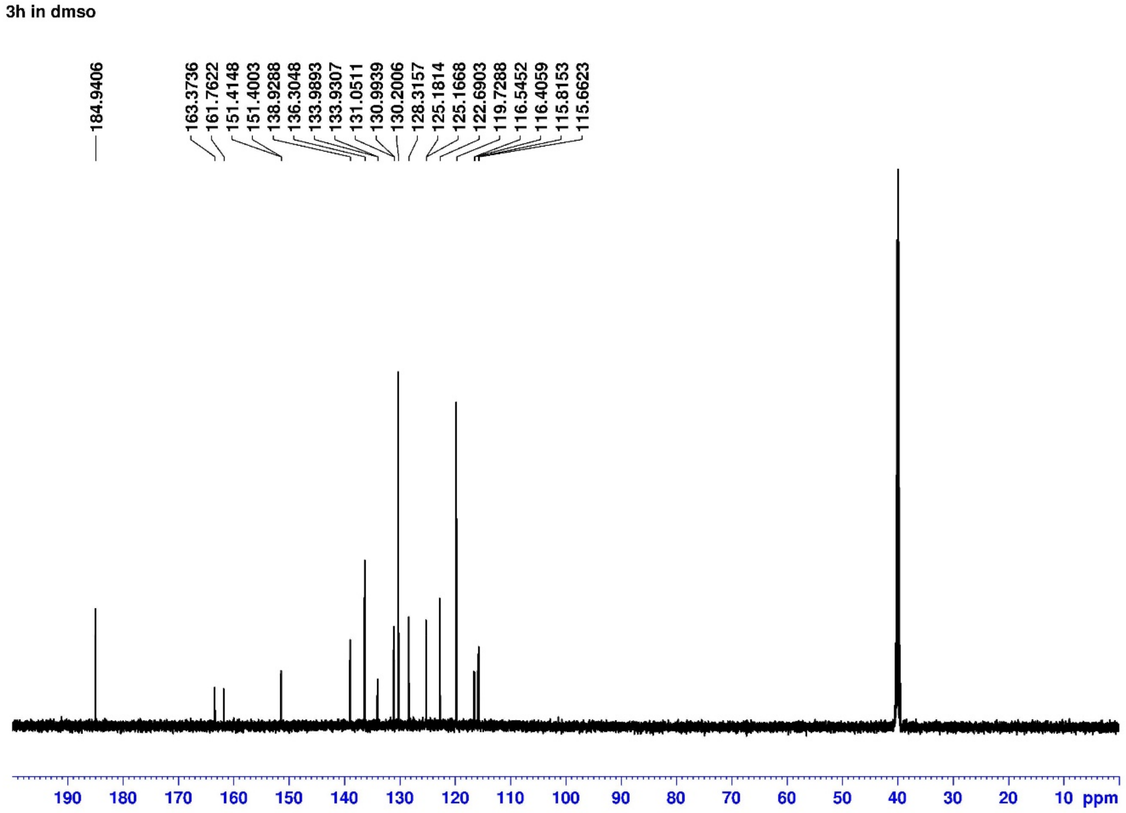

^{13}CNMR spectra of **4l**.

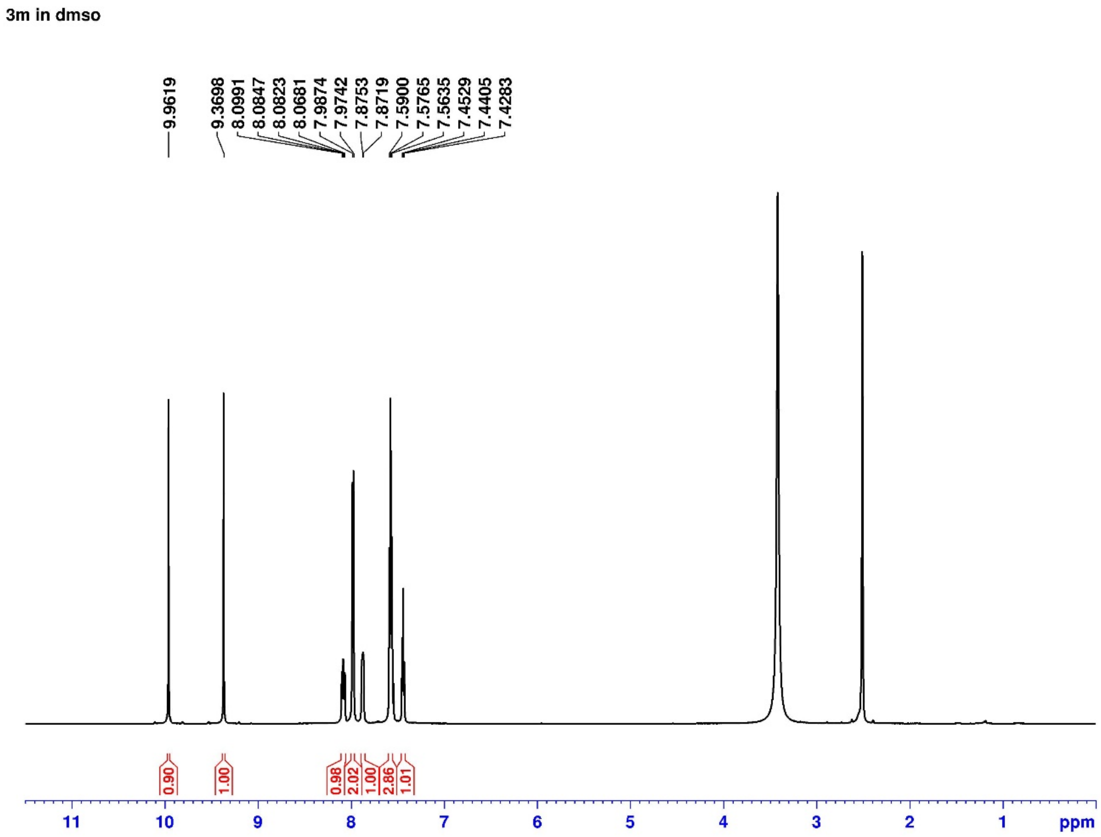

^{1}HNMR spectra of **4m**.

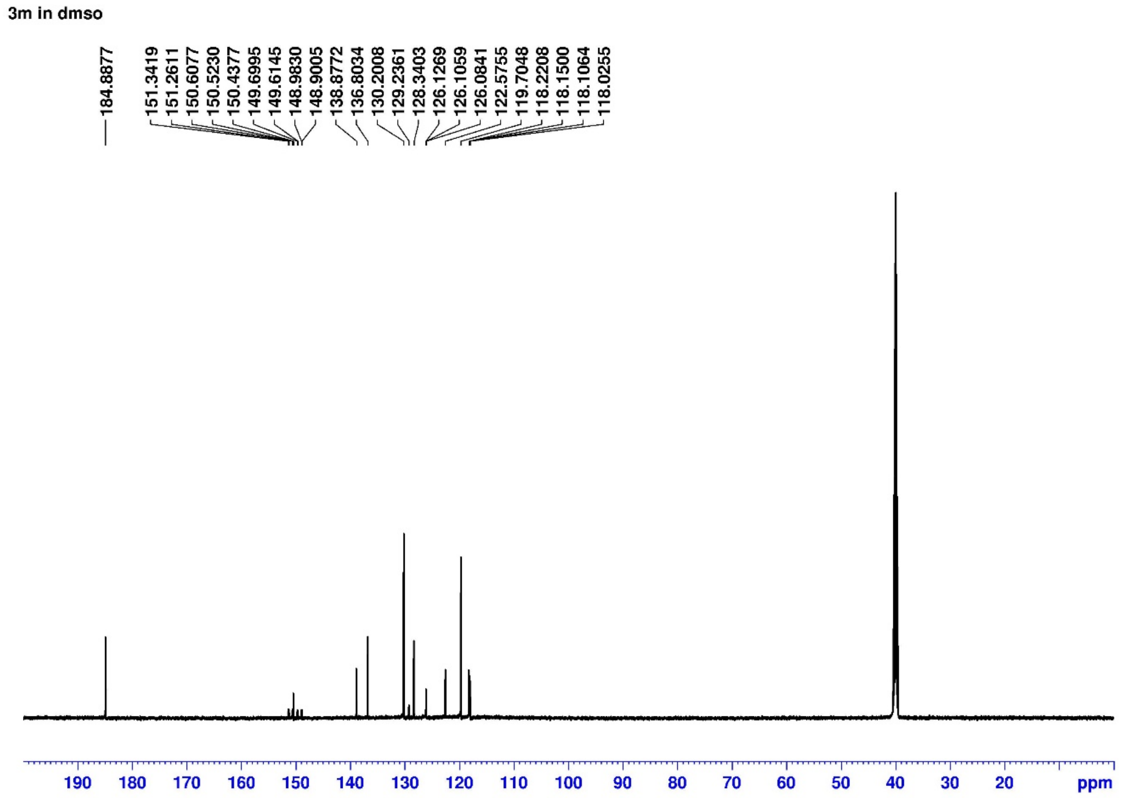

^{13}CNMR spectra of **4m**.

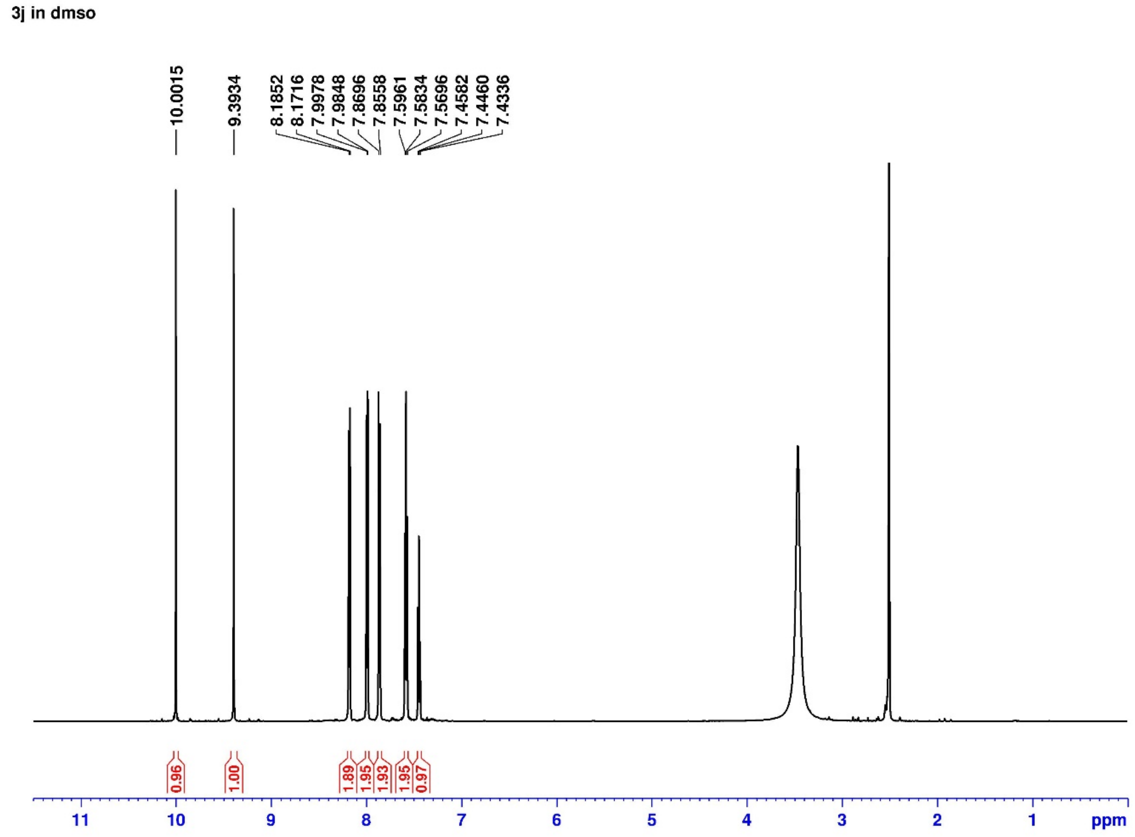

^{1}HNMR spectra of **4n**.

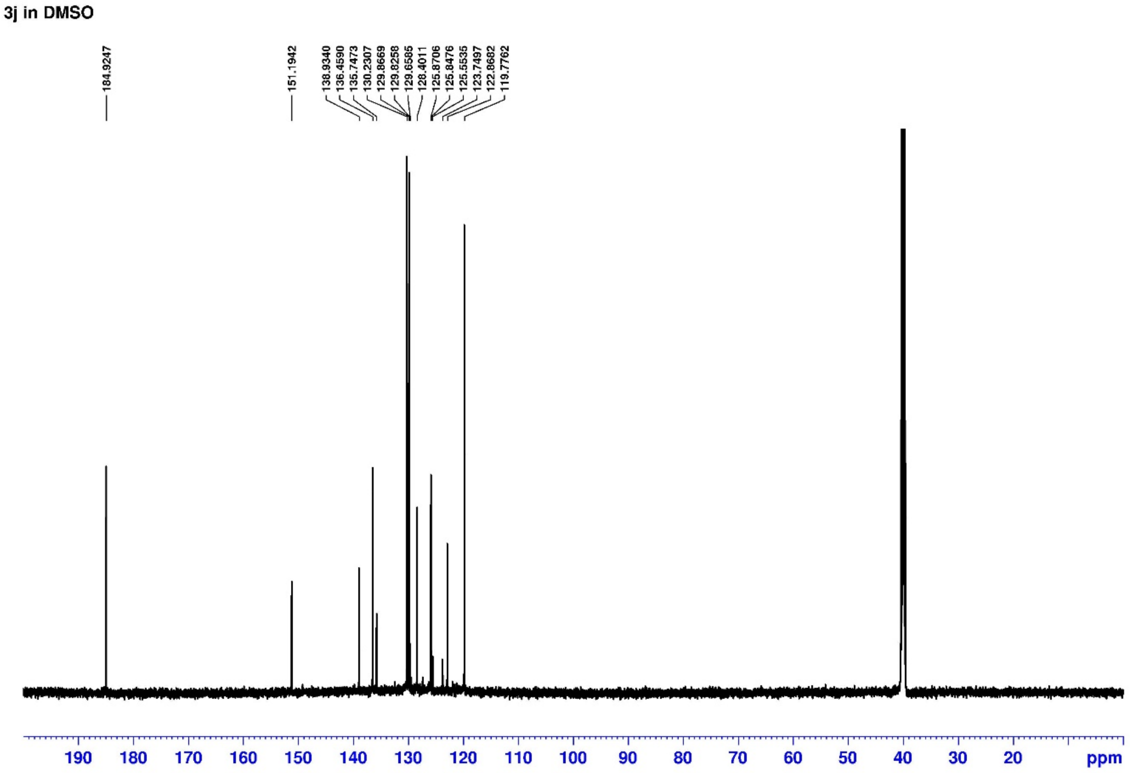

^{13}CNMR spectra of **4n**.

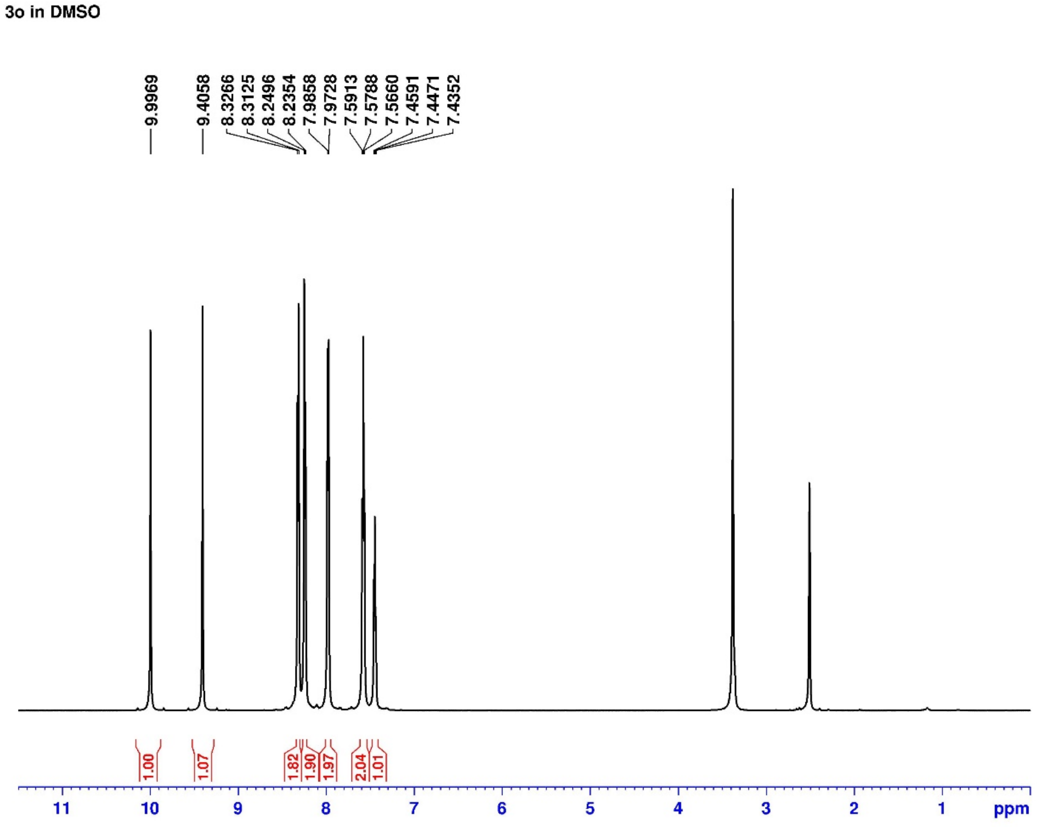

^{1}HNMR spectra of **4o**.

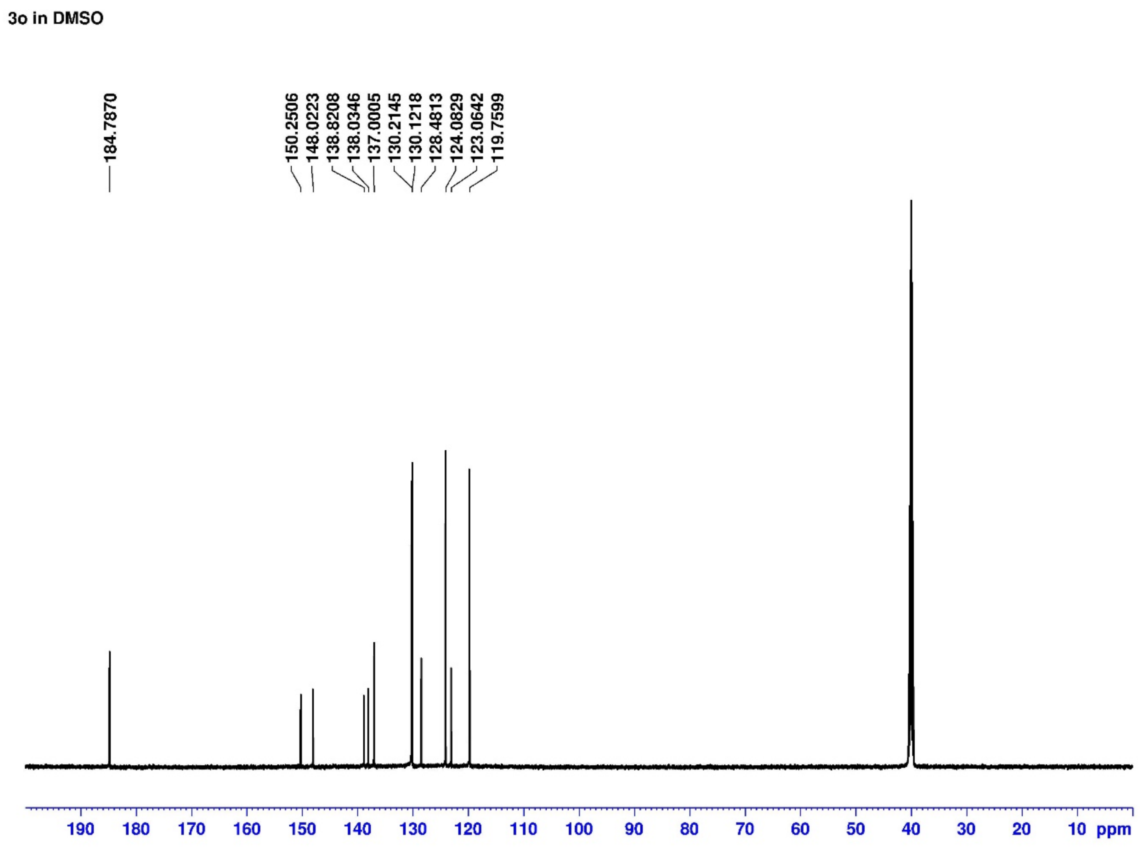

^{13}CNMR spectra of **4o**.

2. **Spectral data of series 6**

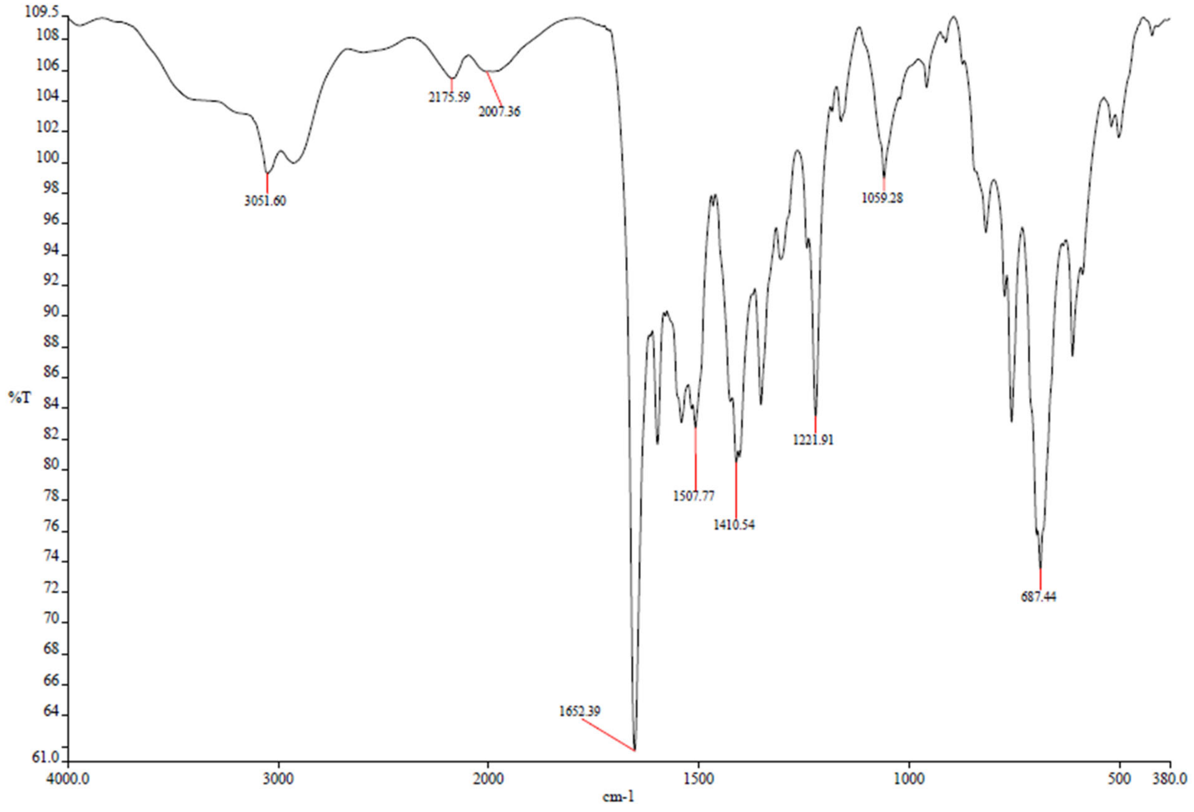

IR spectra of **6a**.

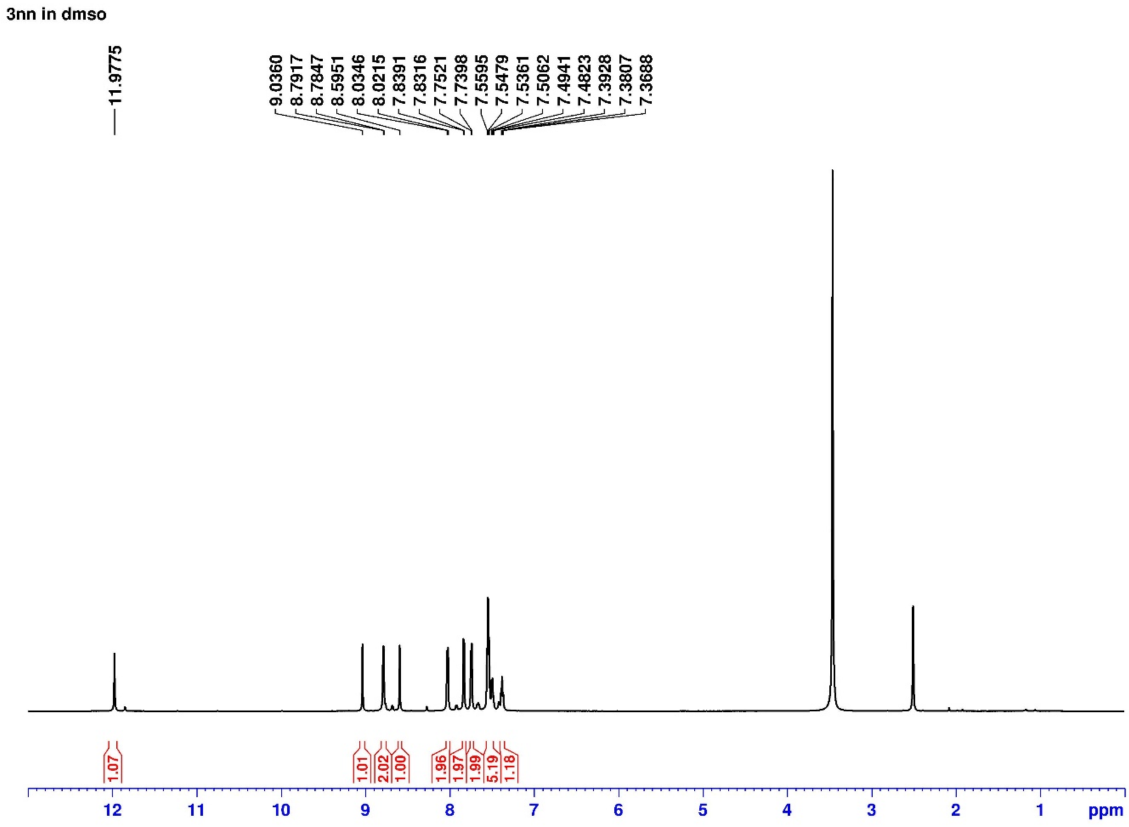

^{1}HNMR spectra of **6a**.

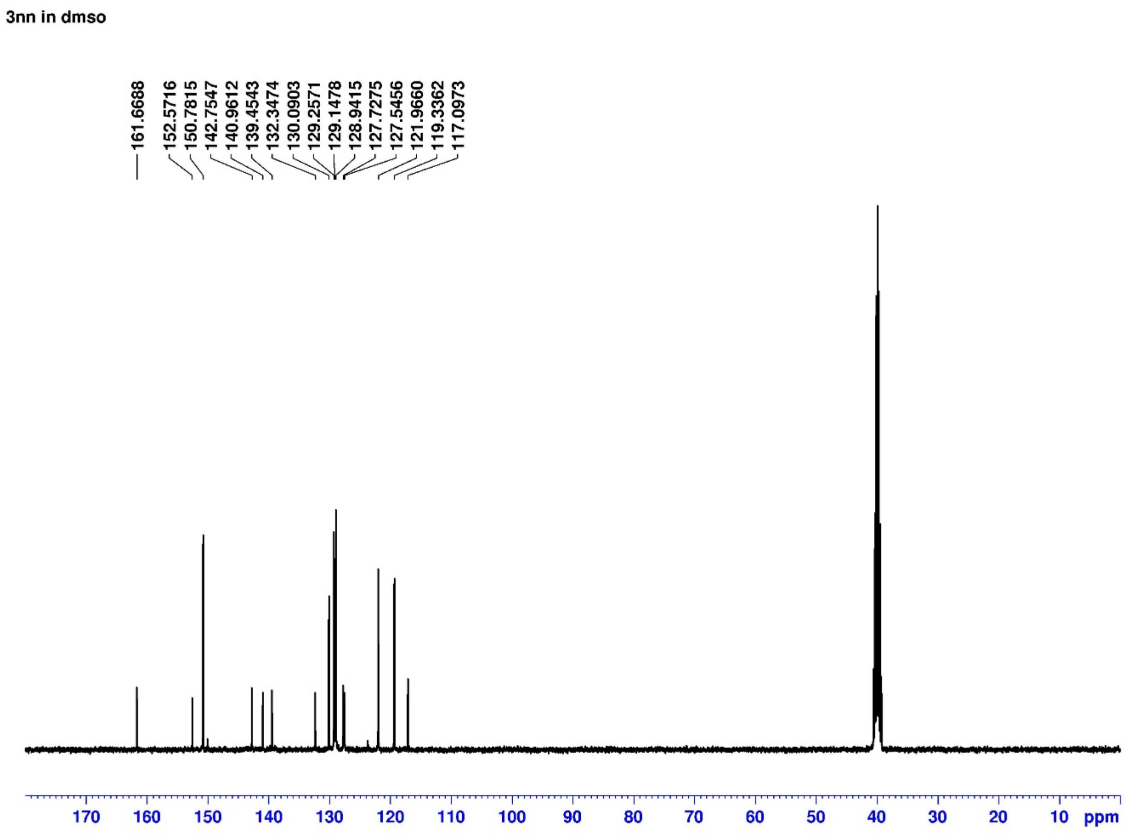

^{13}CNMR spectra of **6a**.

Elemental Composition Report

Single Mass Analysis

Tolerance = 5.0 PPM / DBE: min = -5.0, max = 50.0
Element prediction: Off
Number of isotope peaks used for i-FIT = 3

Monoisotopic Mass, Even Electron Ions
68 formula(e) evaluated with 1 results within limits (all results (up to 1000) for each mass)
Elements Used:
C: 0-80 H: 0-100 N: 0-5 O: 0-1 Na: 1-1

MG_3NN 164 (1.423) Cm (1:230)
TOF MS ES+

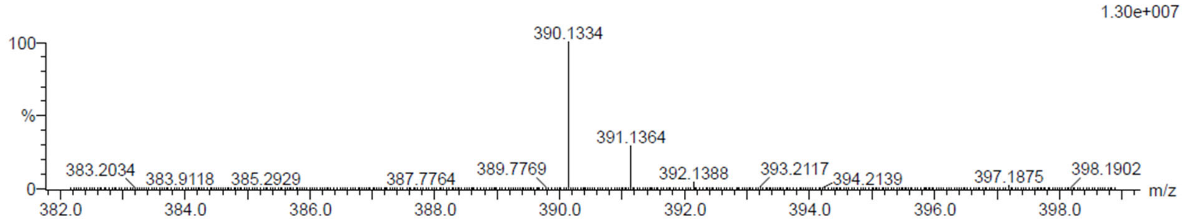

Minimum:				-5.0				
Maximum:		100.0	5.0	50.0				
Mass	Calc. Mass	mDa	PPM	DBE	i-FIT	Norm	Conf(%)	Formula
390.1334	390.1331	0.3	0.8	16.5	1141.6	n/a	n/a	C22 H17 N5 O Na

HRMS of **6a**.

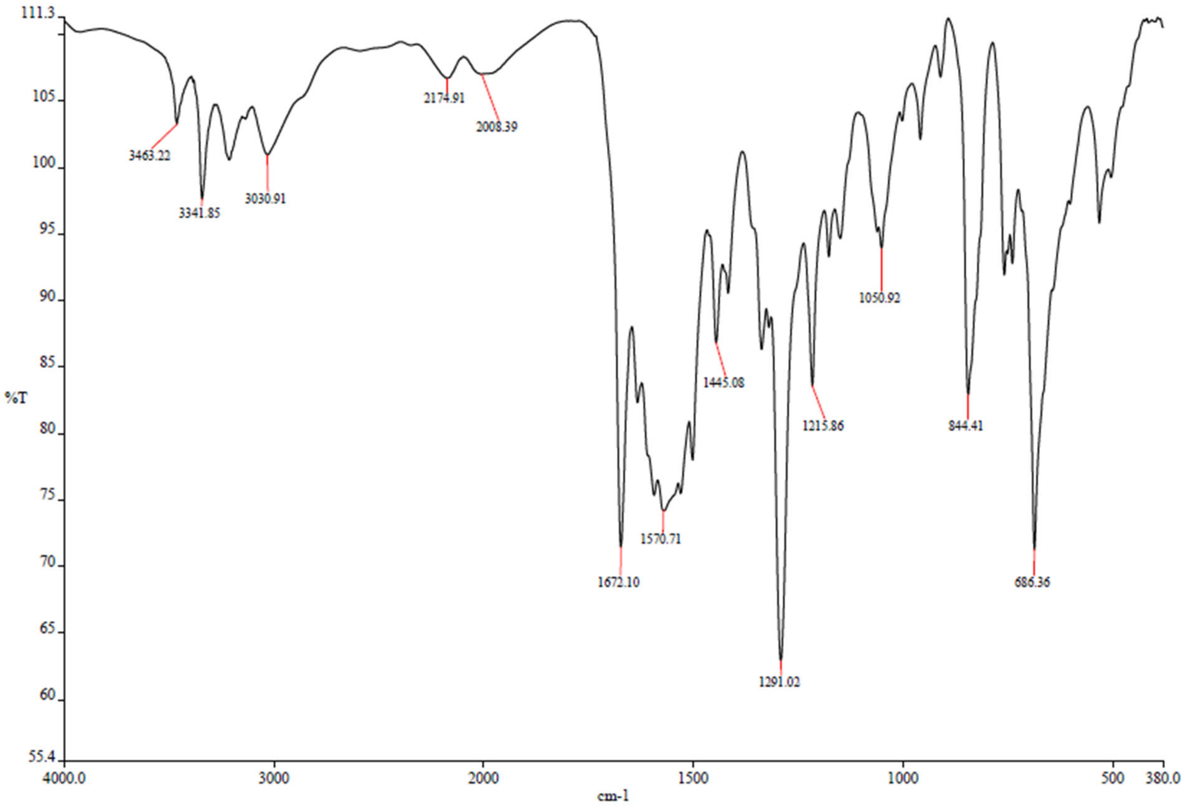

IR spectra of **6b**.

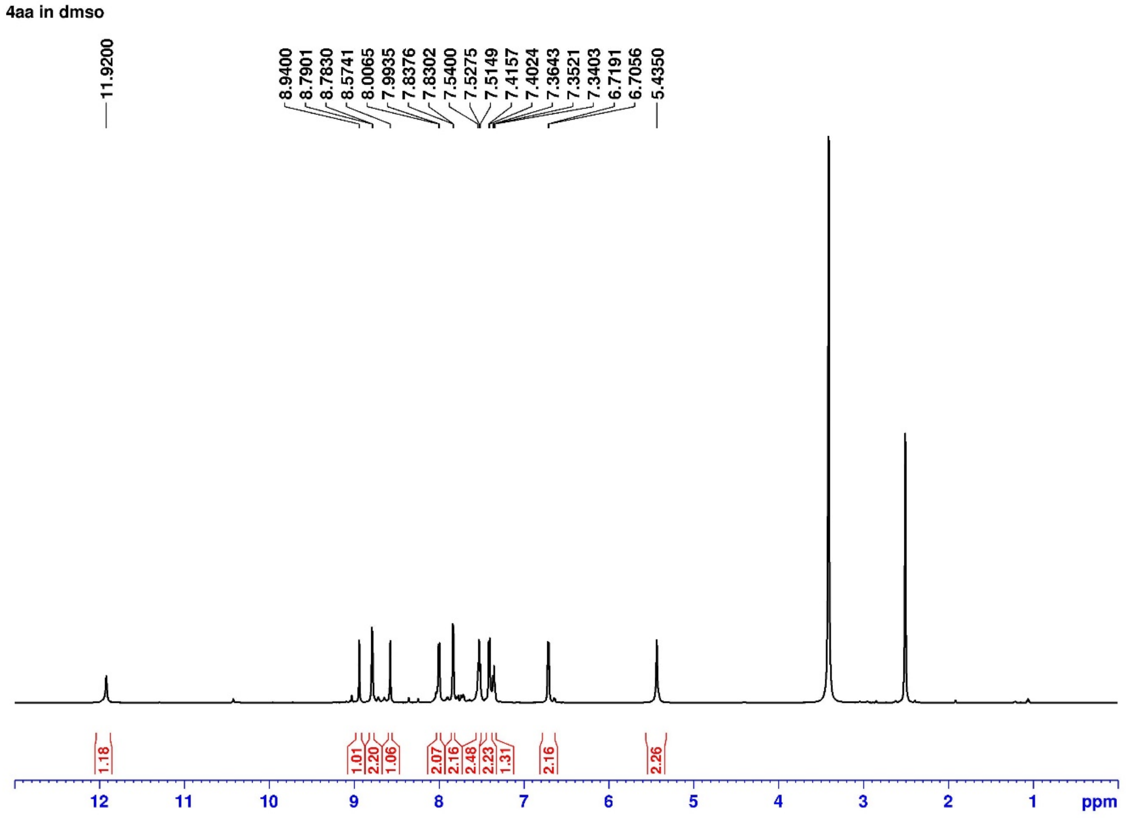

^{1}HNMR spectra of **6b**.

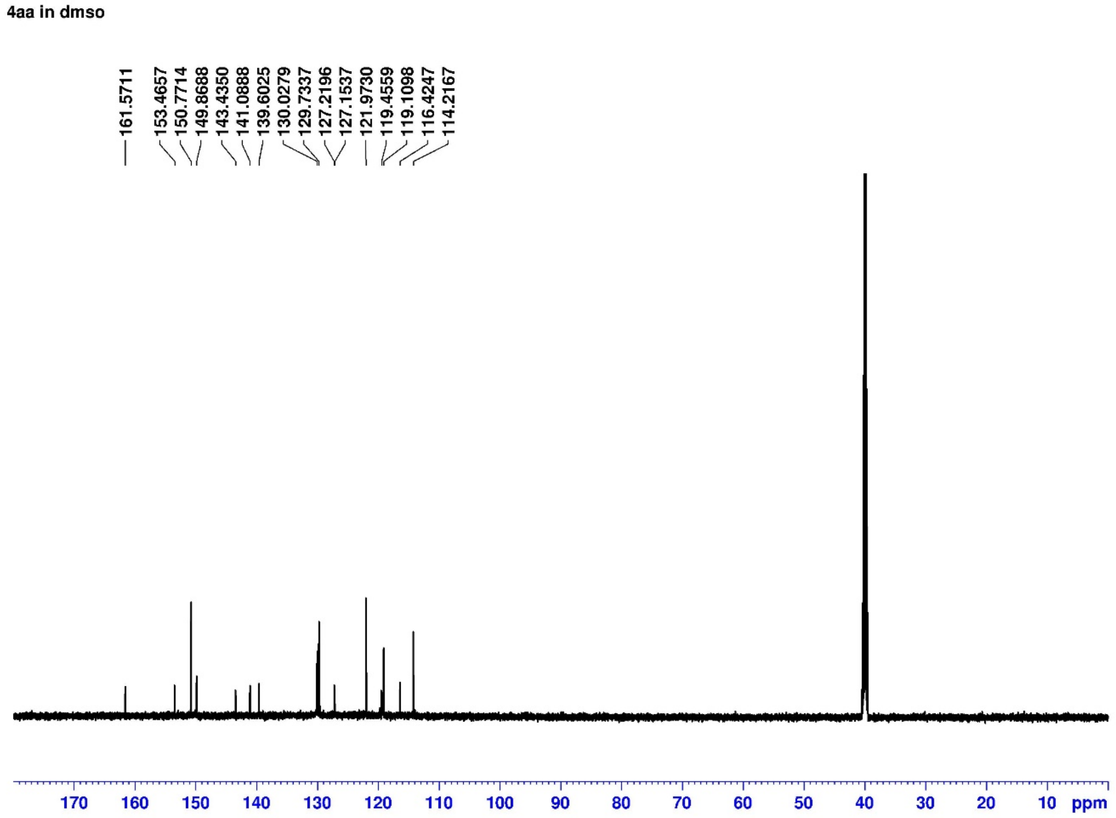

^{13}CNMR spectra of **6b**.

Elemental Composition Report

Page 1

Single Mass Analysis

Tolerance = 5.0 PPM / DBE: min = -5.0, max = 50.0
Element prediction: Off
Number of isotope peaks used for i-FIT = 3

Monoisotopic Mass, Even Electron Ions
171 formula(e) evaluated with 1 results within limits (all results (up to 1000) for each mass)
Elements Used:
C: 0-80 H: 0-100 N: 5-10 O: 0-5 Na: 1-1
MG 3BB 128 (1.113) Cm (1:230)
TOF MS ES+

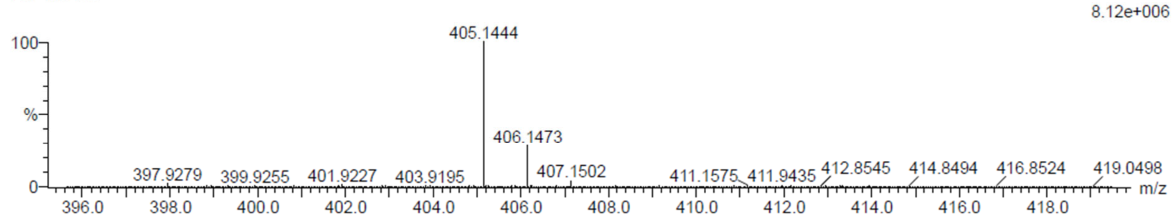

Minimum:				-5.0				
Maximum:		100.0	5.0	50.0				
Mass	Calc. Mass	mDa	PPM	DBE	i-FIT	Norm	Conf(%)	Formula
405.1444	405.1440	0.4	1.0	16.5	1092.1	n/a	n/a	C22 H18 N6 O Na

HRMS of **6b**.

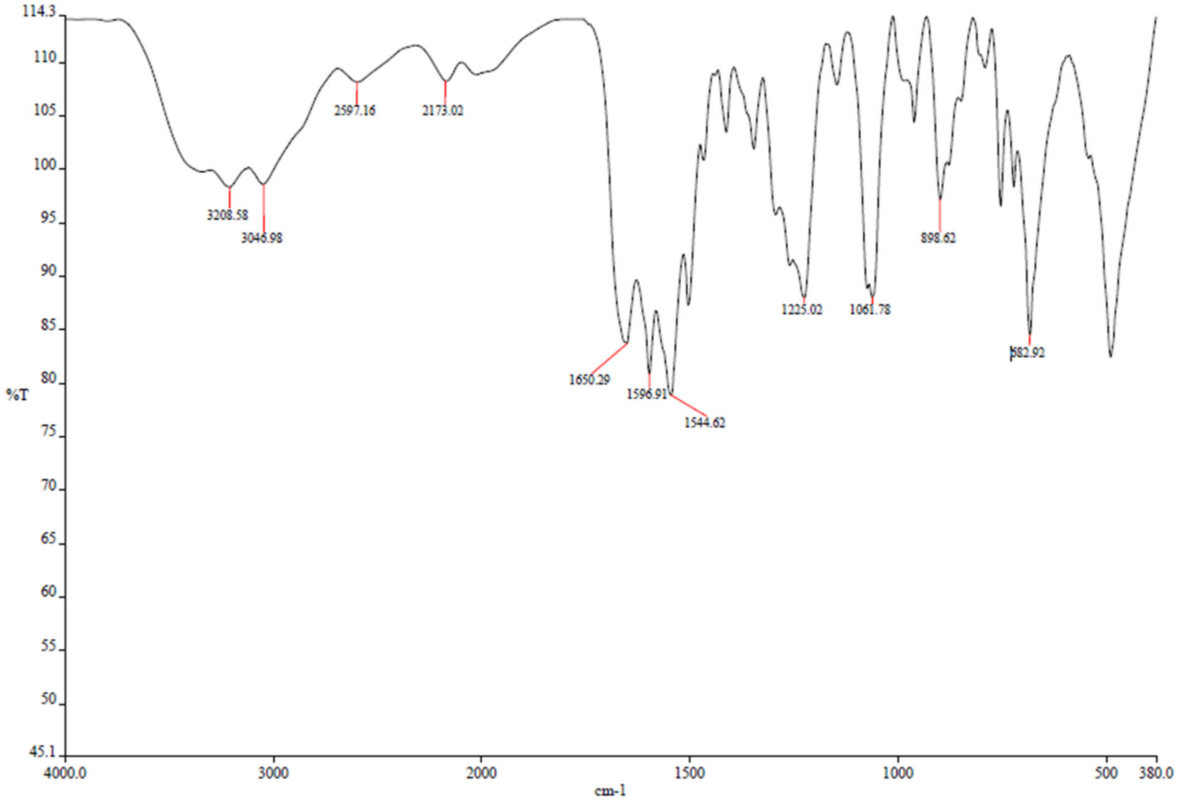

IR spectra of **6c**.

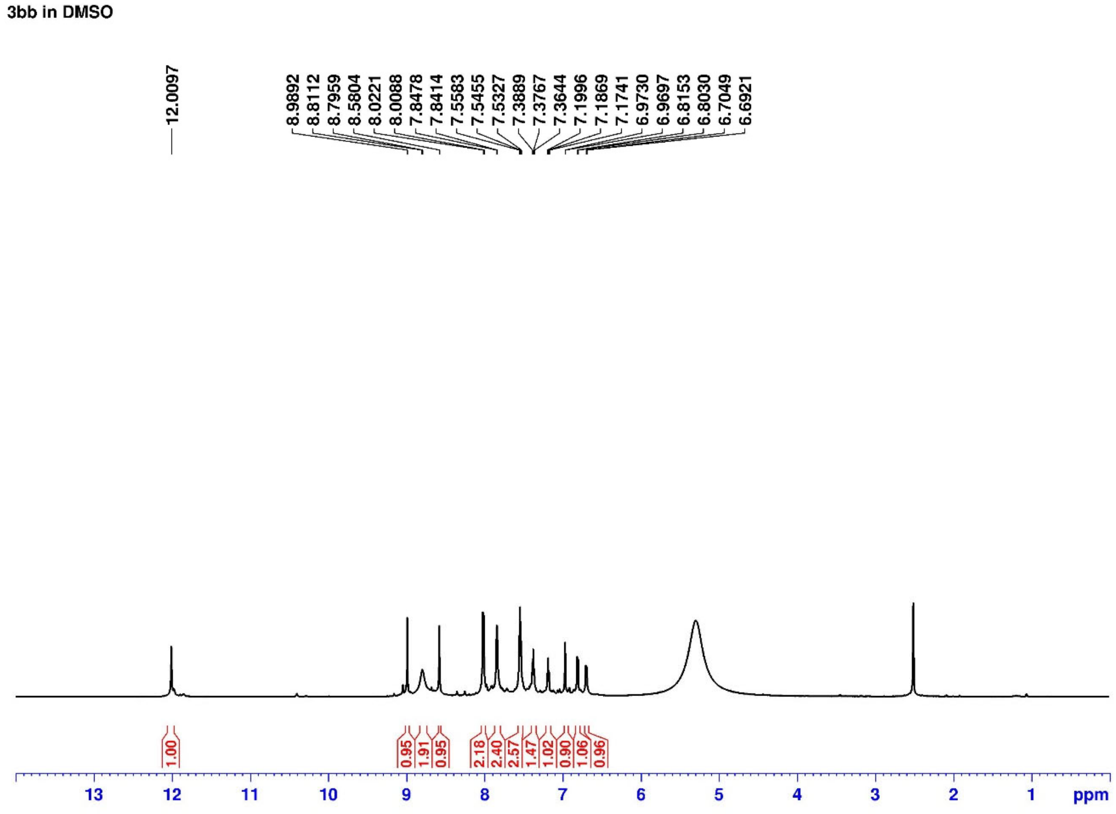

^{1}HNMR spectra of **6c**.

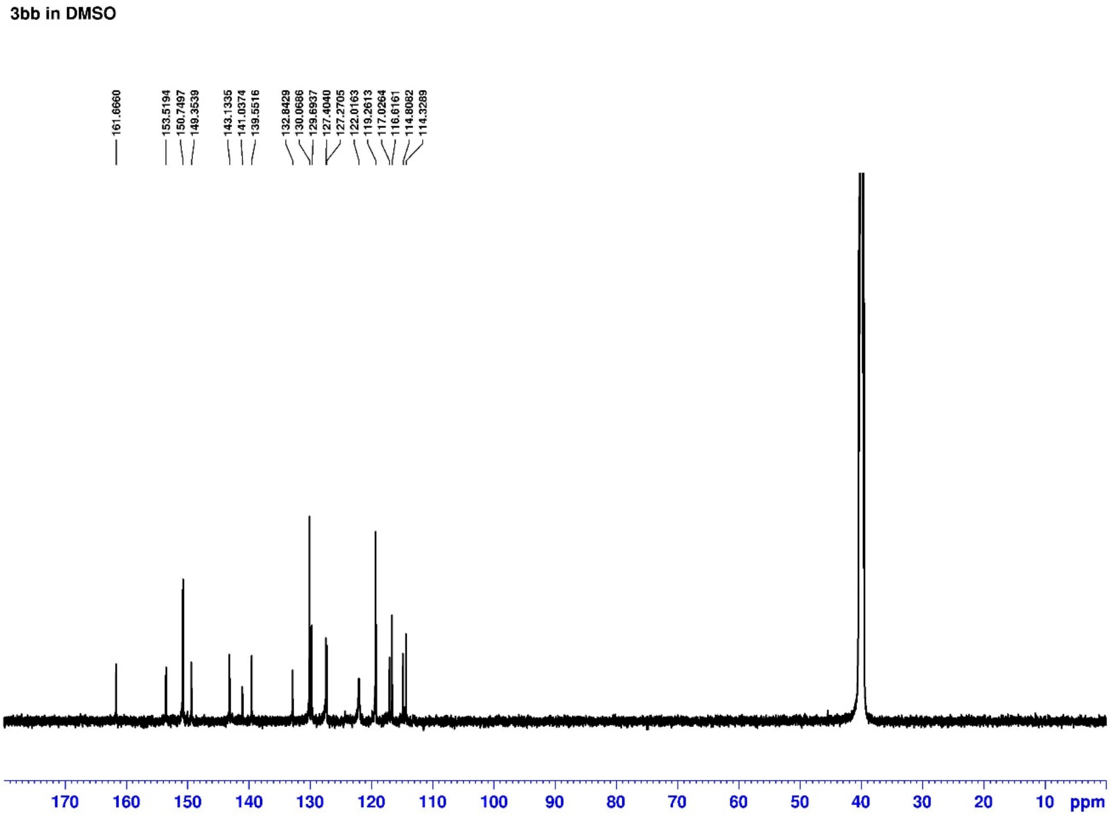

^{13}CNMR spectra of **6c**.

Elemental Composition Report

Page 1

Single Mass Analysis

Tolerance = 5.0 PPM / DBE: min = -5.0, max = 50.0
Element prediction: Off
Number of isotope peaks used for i-FIT = 3

Monoisotopic Mass, Even Electron Ions
171 formula(e) evaluated with 1 results within limits (all results (up to 1000) for each mass)
Elements Used:
C: 0-80 H: 0-100 N: 5-10 O: 0-5 Na: 1-1
MG 3AA 1 (0.024) Cm (1:230)
TOF MS ES+

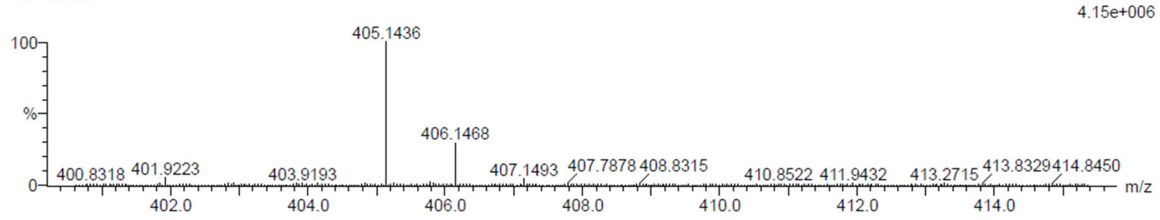

Minimum:				-5.0				
Maximum:		100.0	5.0	50.0				
Mass	Calc. Mass	mDa	PPM	DBE	i-FIT	Norm	Conf(%)	Formula
405.1436	405.1440	-0.4	-1.0	16.5	1065.4	n/a	n/a	C22 H18 N6 O Na

HRMS of **6c**.

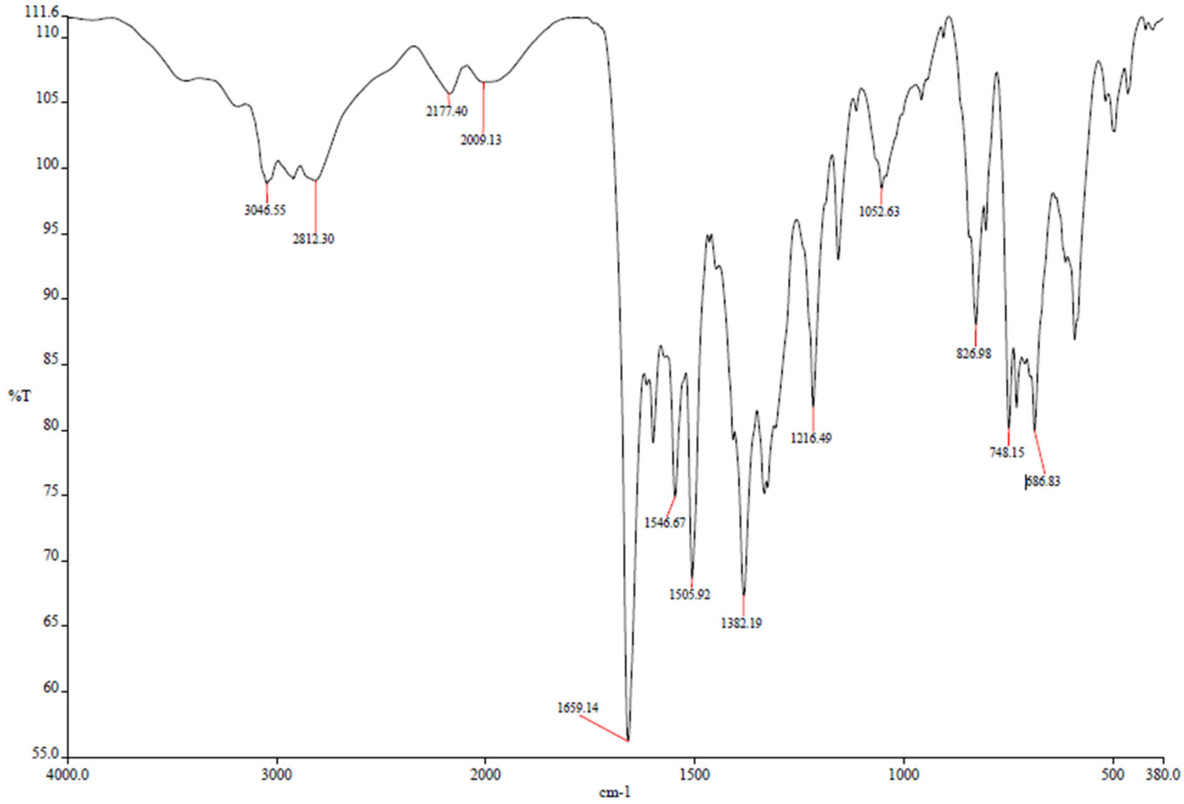

IR spectra of **6d**.

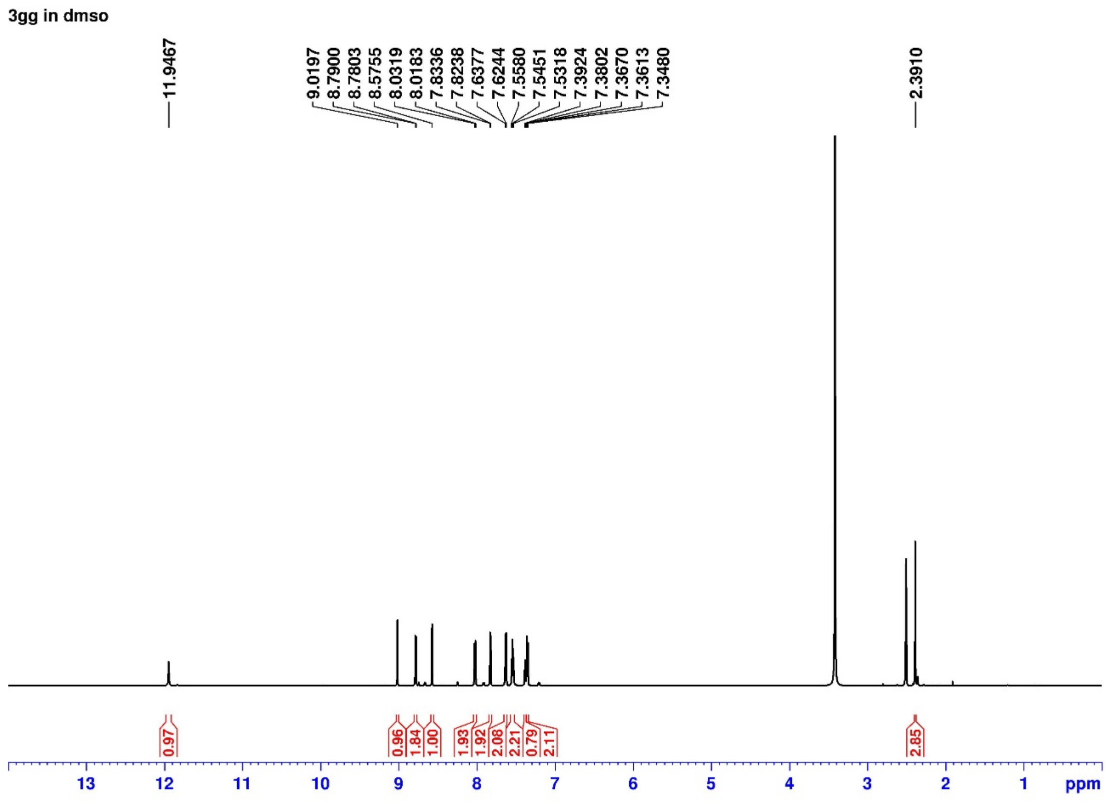

^{1}HNMR spectra of **6d**.

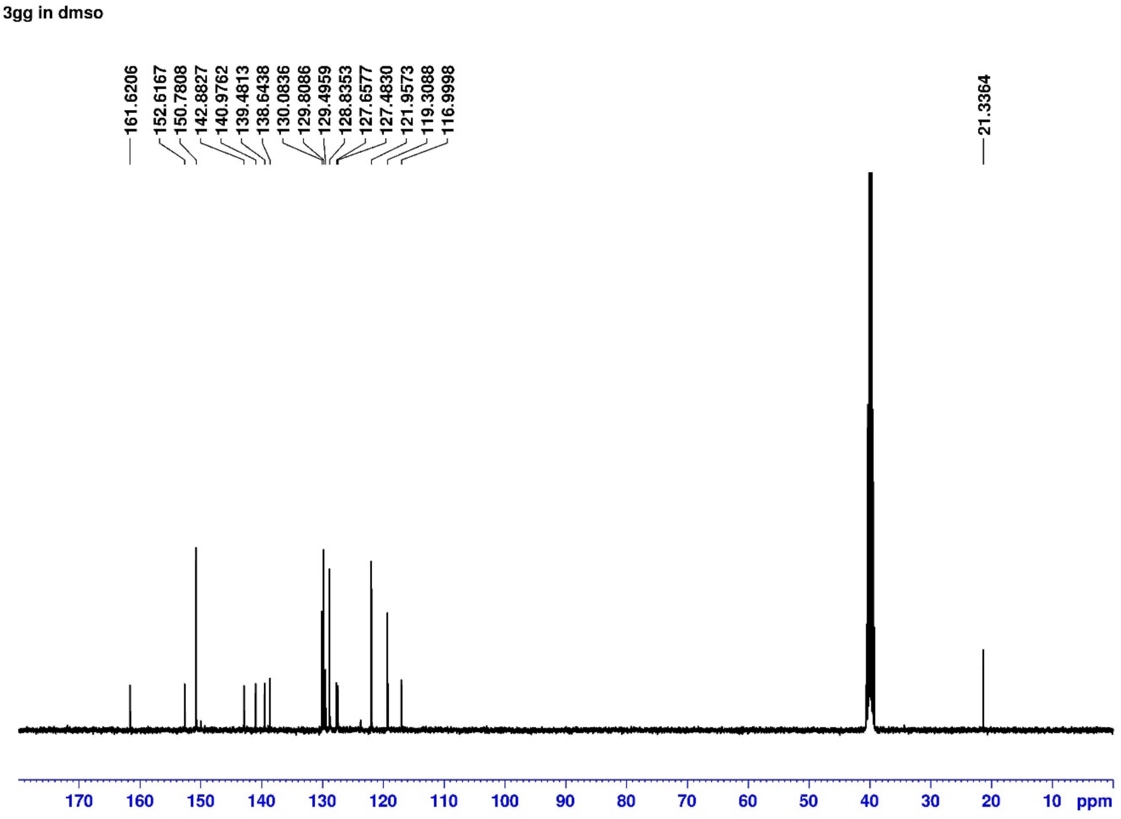

^{13}CNMR spectra of **6d**.

Elemental Composition Report **Page 1**

Single Mass Analysis
Tolerance = 5.0 PPM / DBE: min = -5.0, max = 50.0
Element prediction: Off
Number of isotope peaks used for i-FIT = 3

Monoisotopic Mass, Even Electron Ions
174 formula(e) evaluated with 1 results within limits (all results (up to 1000) for each mass)
Elements Used:
C: 0-80 H: 0-100 N: 5-10 O: 0-5 Na: 1-1
MG 3GG 222 (1.920) Cm (1:230)
TOF MS ES+

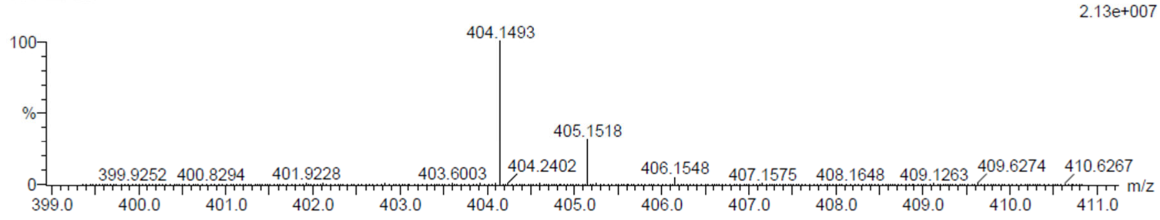

	Mass	Calc. Mass	mDa	PPM	DBE	i-FIT	Norm	Conf(%)	Formula
Minimum:					-5.0				
Maximum:			100.0	5.0	50.0				
	404.1493	404.1487	0.6	1.5	16.5	1267.9	n/a	n/a	C23 H19 N5 O Na

HRMS of **6d**.

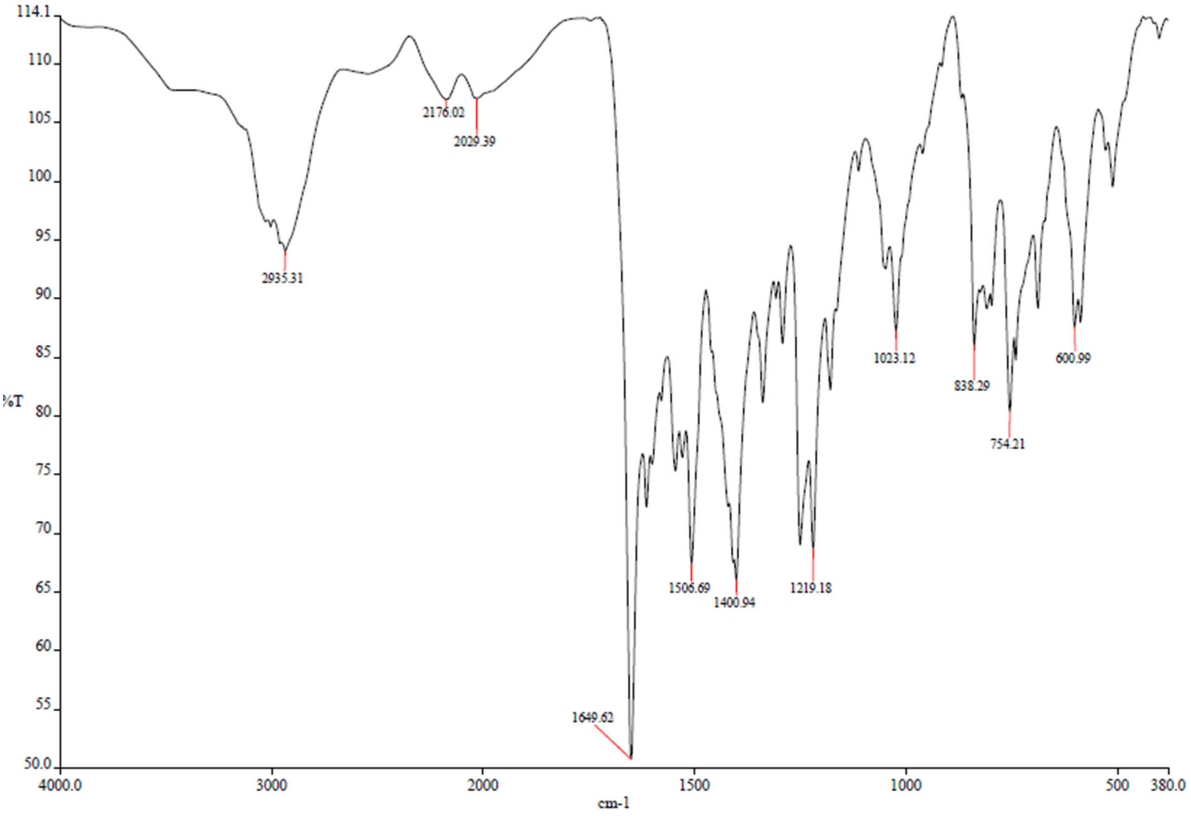

IR spectra of **6e**.

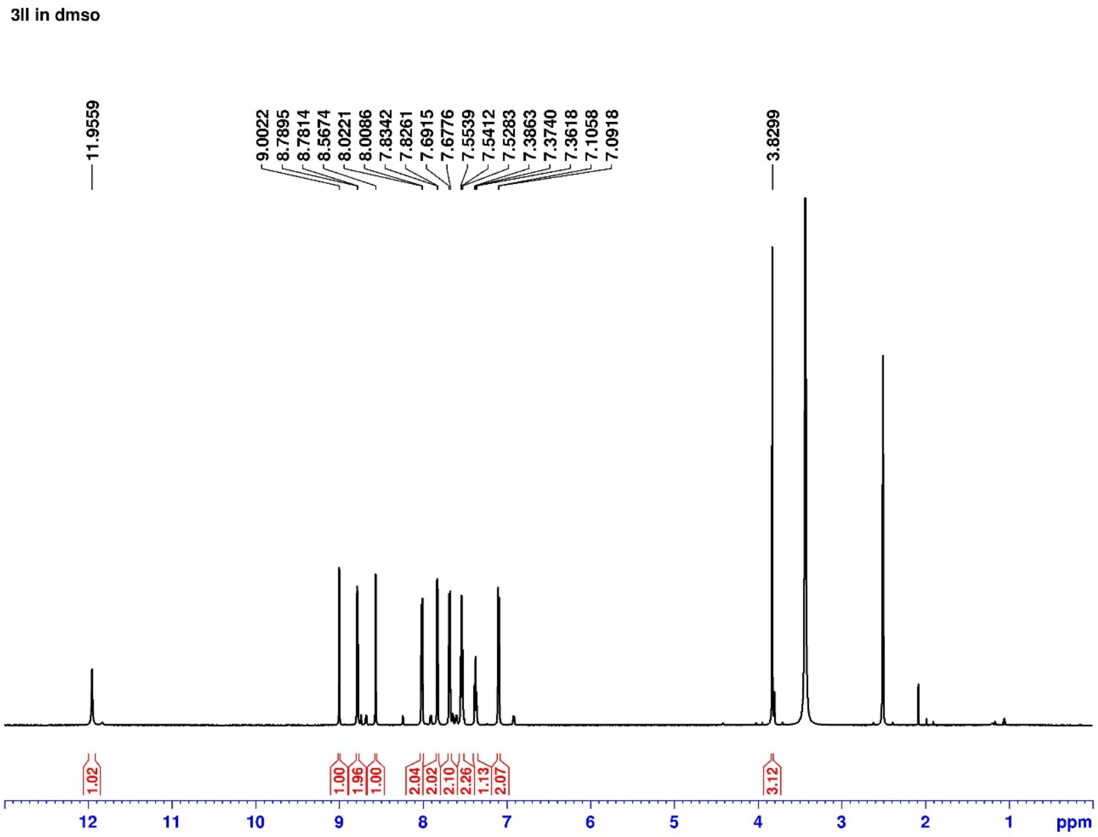

^{1}HNMR spectra of **6e**.

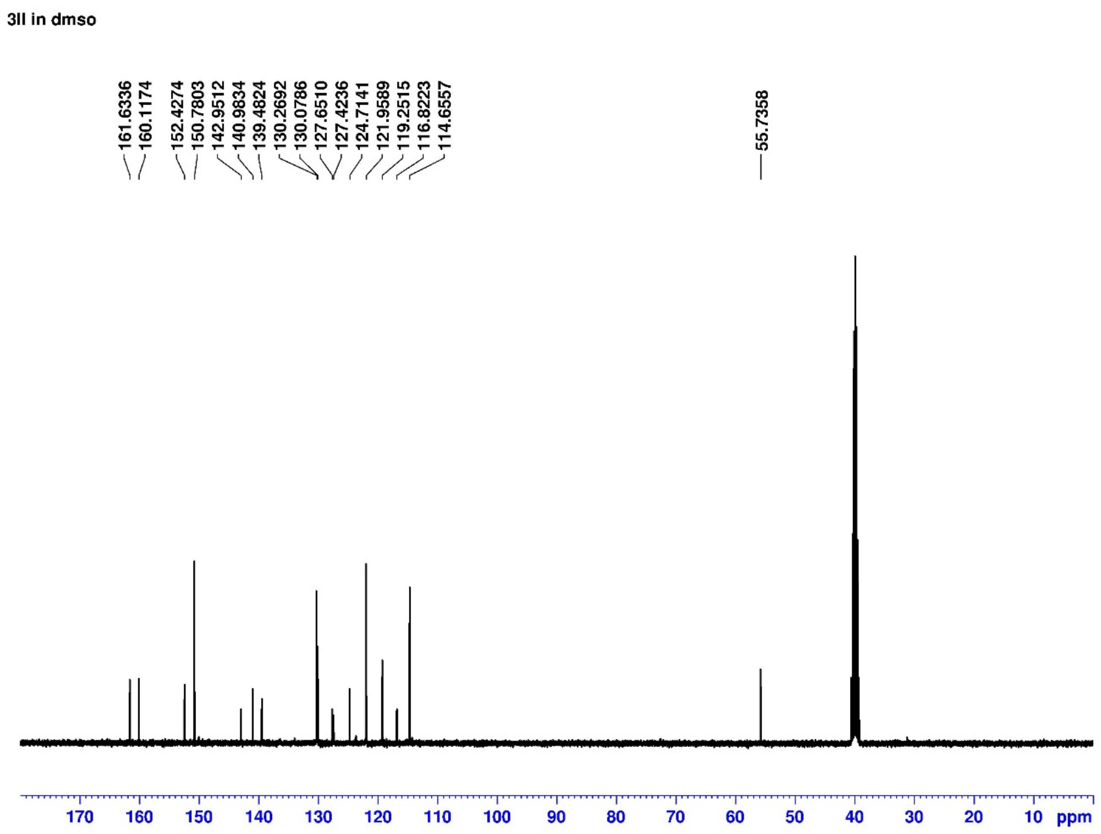

^{13}CNMR spectra of **6e**.

Elemental Composition Report **Page 1**

Single Mass Analysis
Tolerance = 5.0 PPM / DBE: min = -5.0, max = 50.0
Element prediction: Off
Number of isotope peaks used for i-FIT = 3

Monoisotopic Mass, Even Electron Ions
198 formula(e) evaluated with 1 results within limits (all results (up to 1000) for each mass)
Elements Used:
C: 0-80 H: 0-100 N: 0-5 O: 0-5 Na: 1-1
MG 3LL 24 (0.222) Cm (1:231)
TOF MS ES+

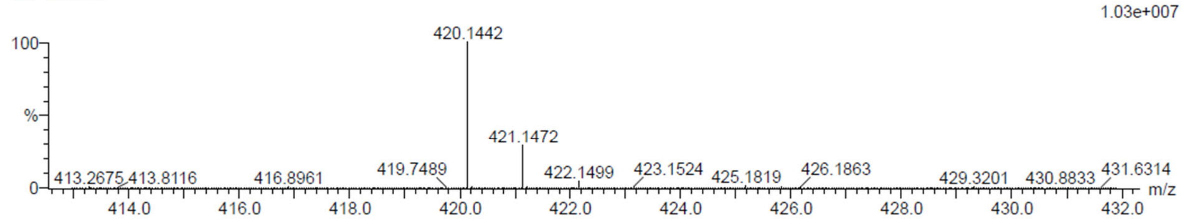

Minimum:				-5.0				
Maximum:		100.0	5.0	50.0				
Mass	Calc. Mass	mDa	PPM	DBE	i-FIT	Norm	Conf(%)	Formula
420.1442	420.1436	0.6	1.4	16.5	1141.8	n/a	n/a	C23 H19 N5 O2 Na

HRMS of **6e**

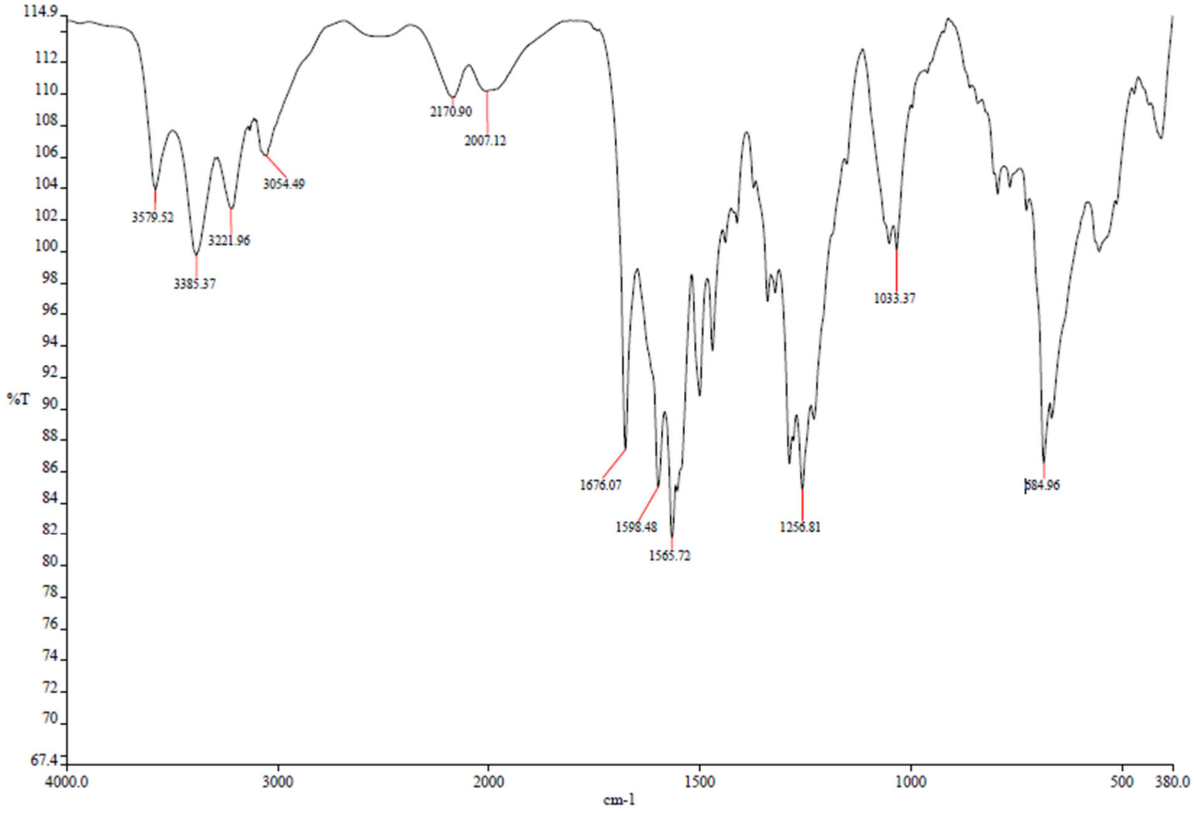

IR spectra of **6f**.

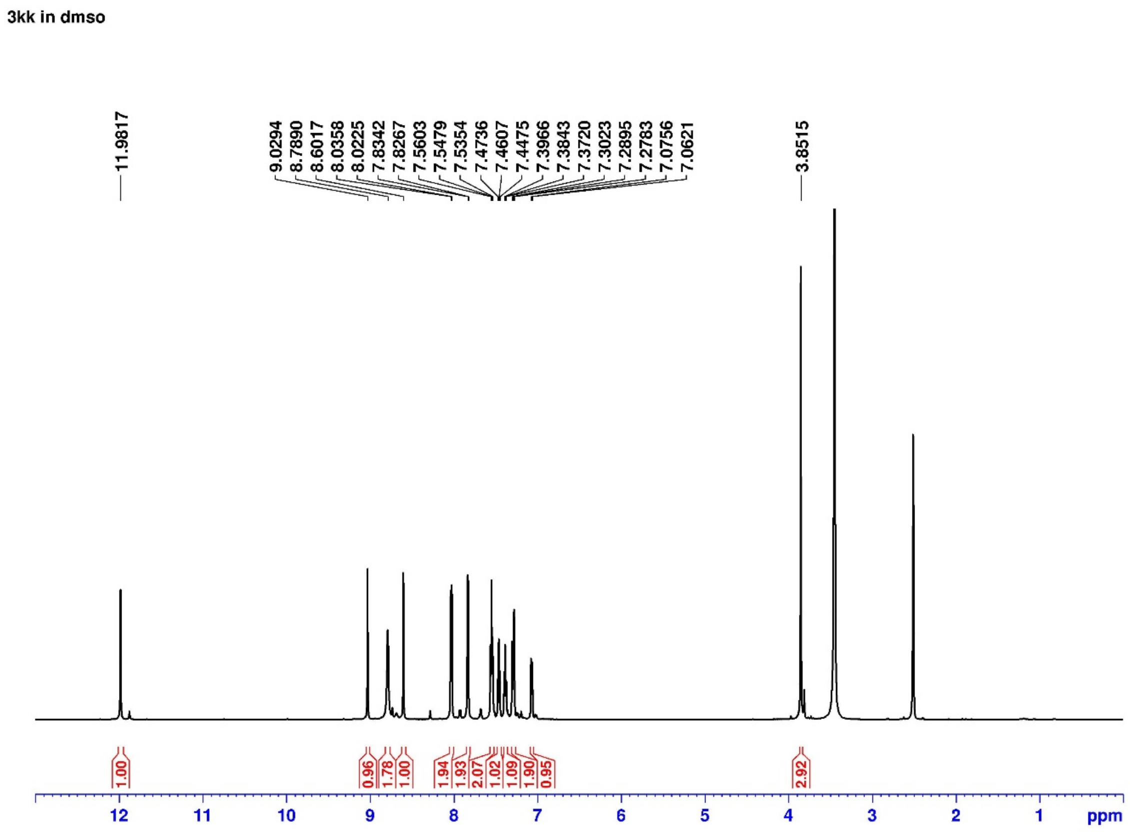

^{1}HNMR spectra of **6f**.

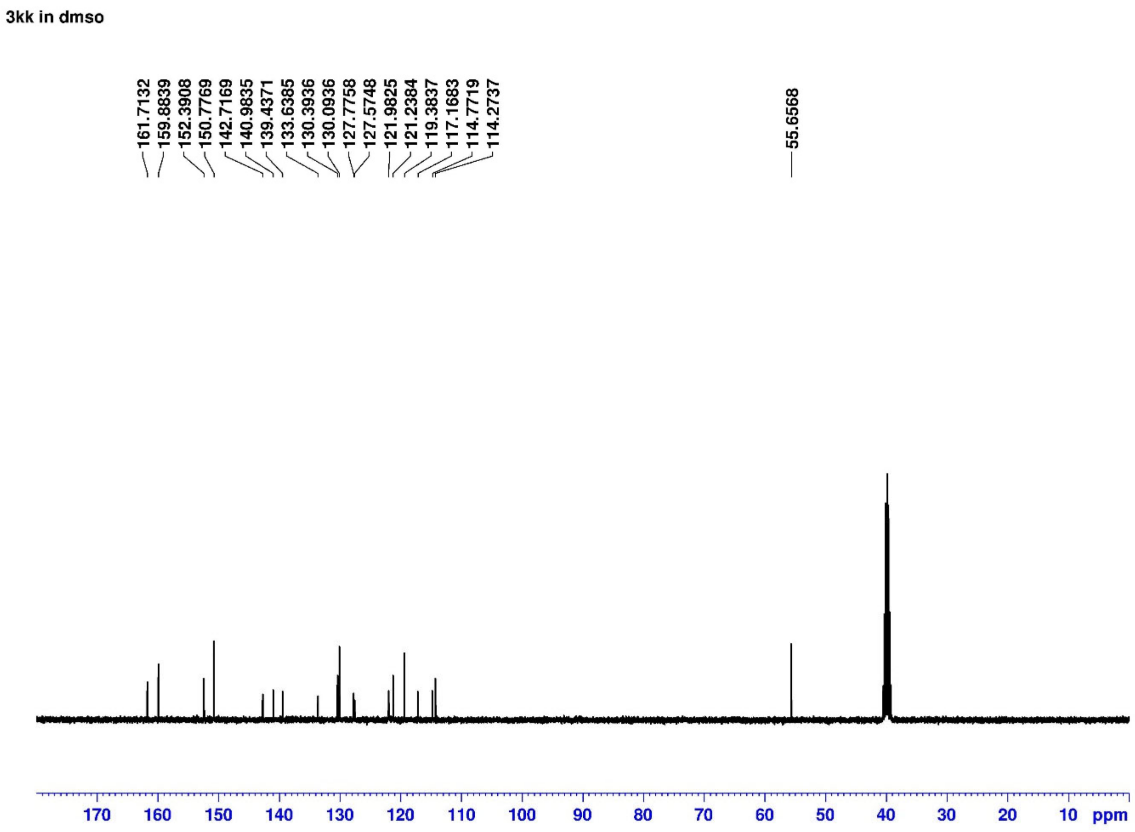

^{13}CNMR spectra of **6f**.

Elemental Composition Report

Page 1

Single Mass Analysis

Tolerance = 5.0 PPM / DBE: min = -5.0, max = 50.0
Element prediction: Off
Number of isotope peaks used for i-FIT = 3

Monoisotopic Mass, Even Electron Ions
198 formula(e) evaluated with 1 results within limits (all results (up to 1000) for each mass)
Elements Used:
C: 0-80 H: 0-100 N: 0-5 O: 0-5 Na: 1-1

MG 3KK 223 (1.928) Cm (1:231)
TOF MS ES+

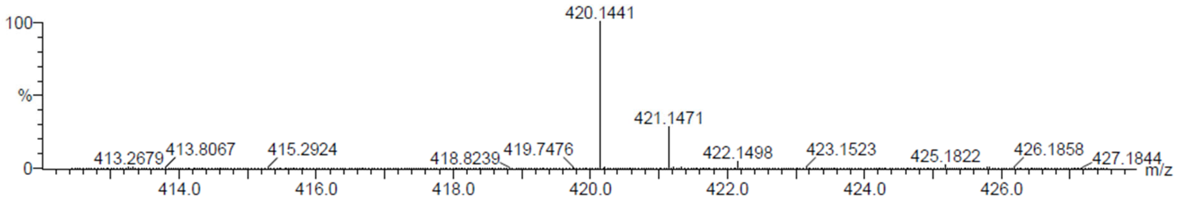

Minimum:				-5.0				
Maximum:		100.0	5.0	50.0				
Mass	Calc. Mass	mDa	PPM	DBE	i-FIT	Norm	Conf(%)	Formula
420.1441	420.1436	0.5	1.2	16.5	1100.6	n/a	n/a	C23 H19 N5 O2 Na

HRMS of **6f**.

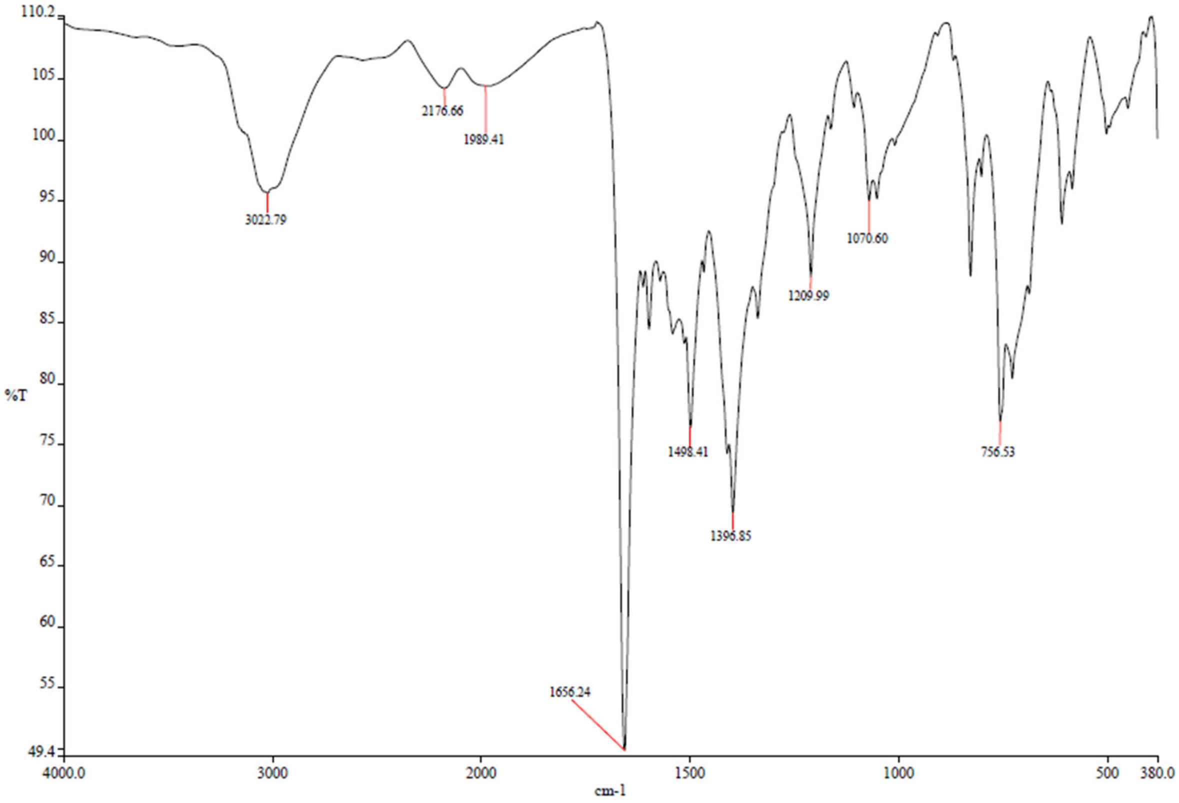

IR spectra of **6g**.

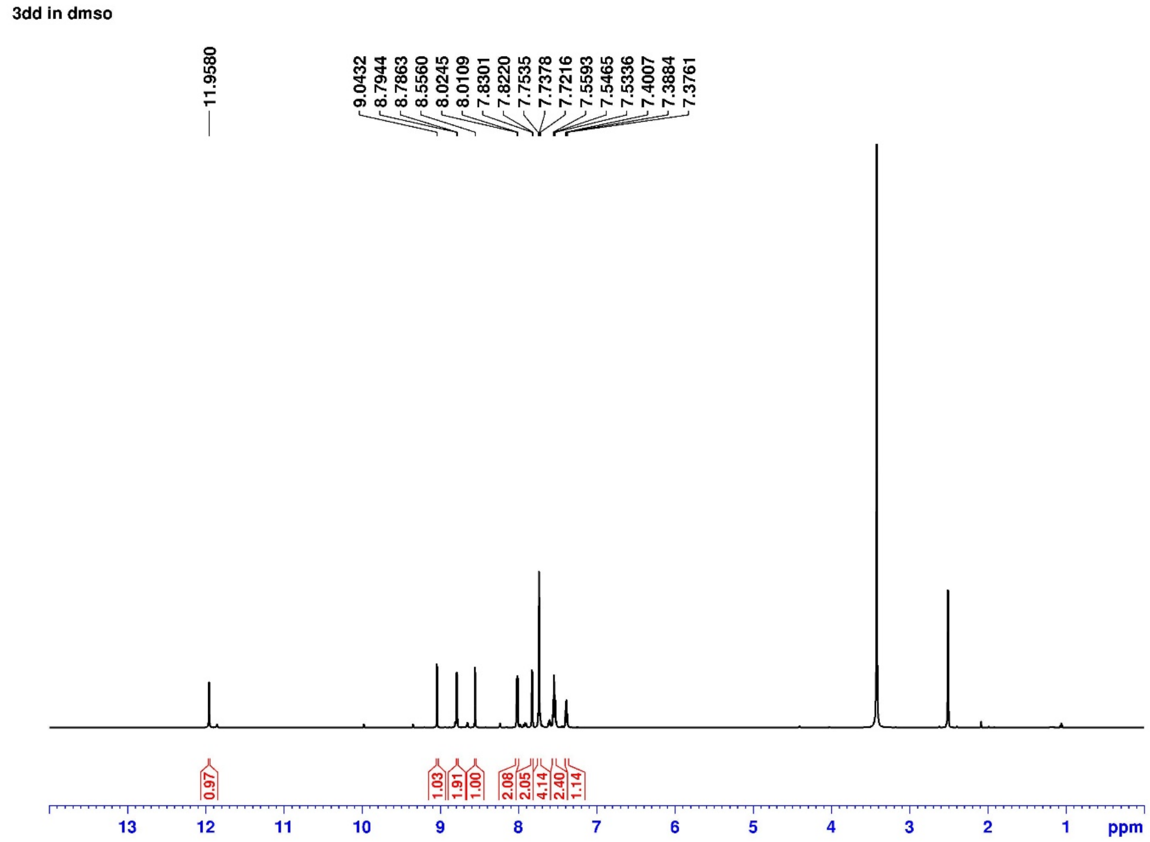

^{1}HNMR spectra of **6g**.

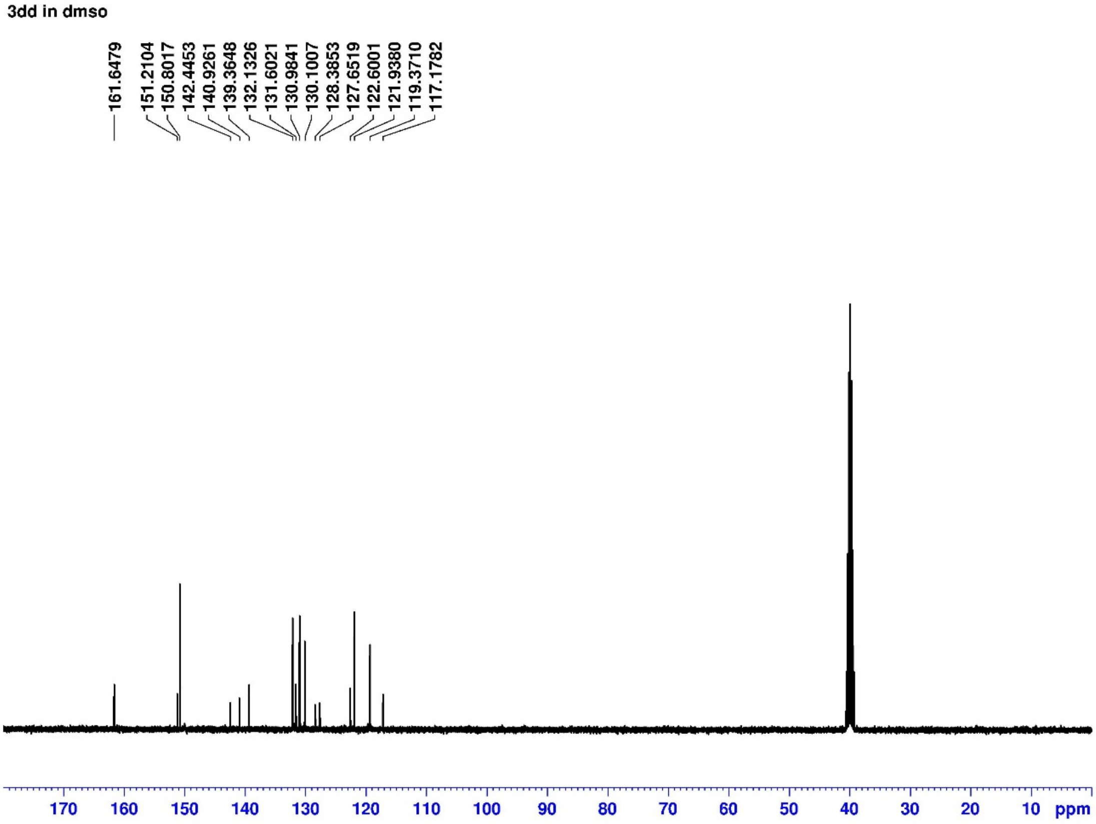

^{13}CNMR spectra of **6g**.

Elemental Composition Report

Page 1

Single Mass Analysis

Tolerance = 5.0 PPM / DBE: min = -5.0, max = 50.0
Element prediction: Off
Number of isotope peaks used for i-FIT = 3

Monoisotopic Mass, Even Electron Ions
162 formula(e) evaluated with 1 results within limits (all results (up to 1000) for each mass)
Elements Used:
C: 0-80 H: 0-100 N: 5-10 O: 0-5 Na: 1-1 Br: 1-1

MG_3DD 23 (0.213) Cm (1:230)
TOF MS ES+

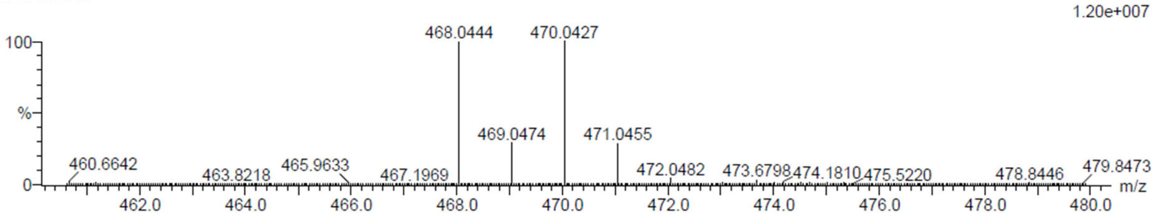

Minimum:				-5.0				
Maximum:		100.0	5.0	50.0				
Mass	Calc. Mass	mDa	PPM	DBE	i-FIT	Norm	Conf(%)	Formula
468.0444	468.0436	0.8	1.7	16.5	1226.9	n/a	n/a	C22 H16 N5 O Na Br

HRMS of **6g**.

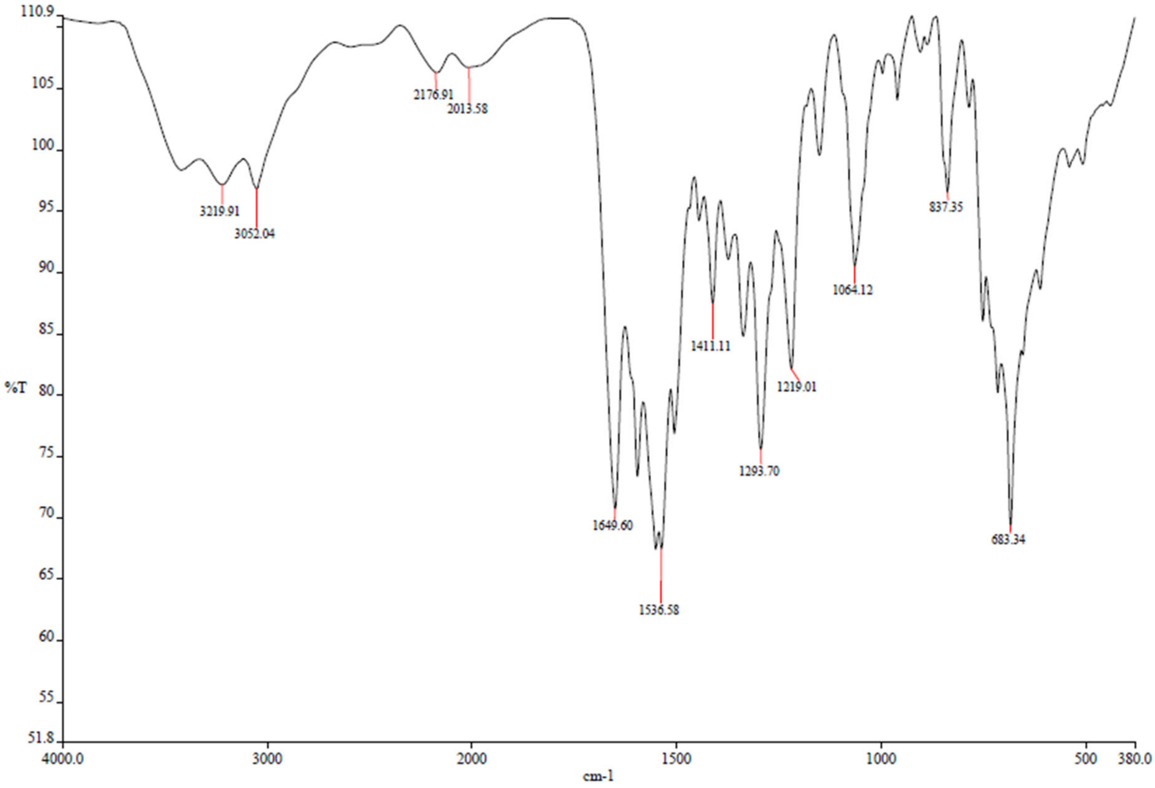

IR spectra of **6h**.

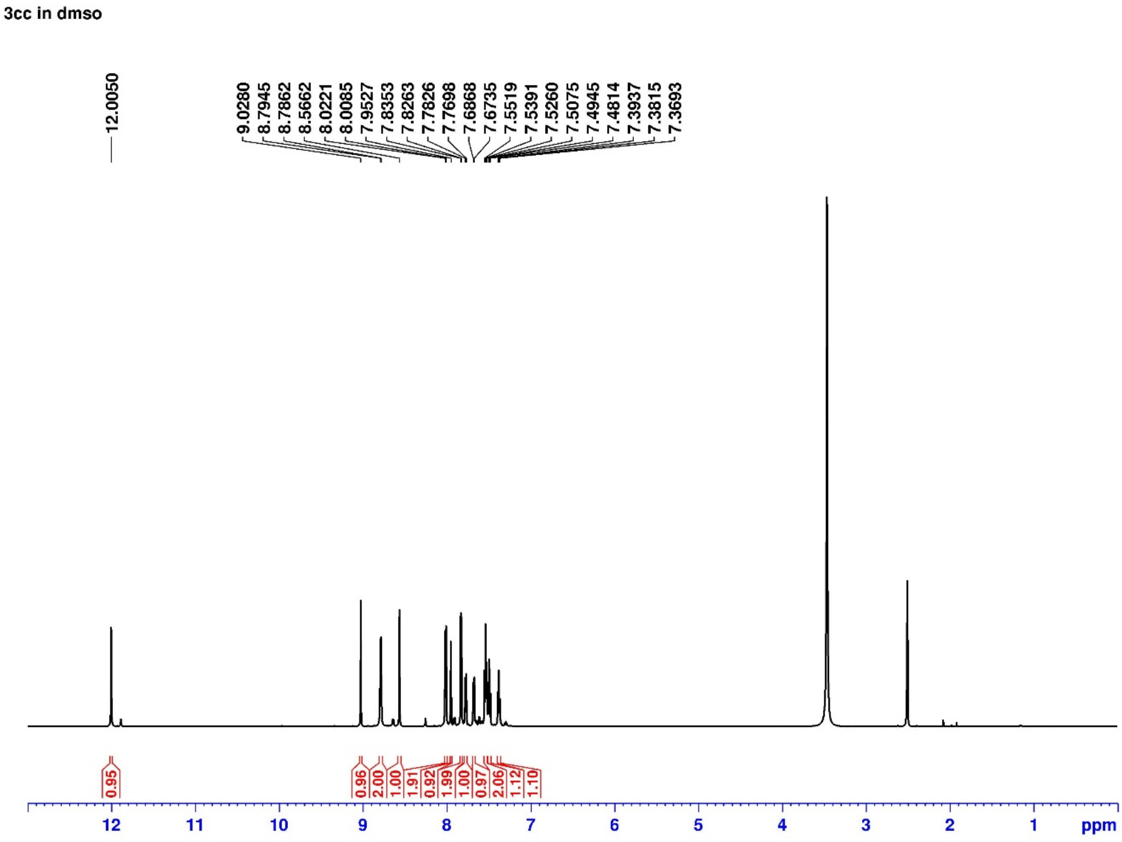

^{1}HNMR spectra of **6h**.

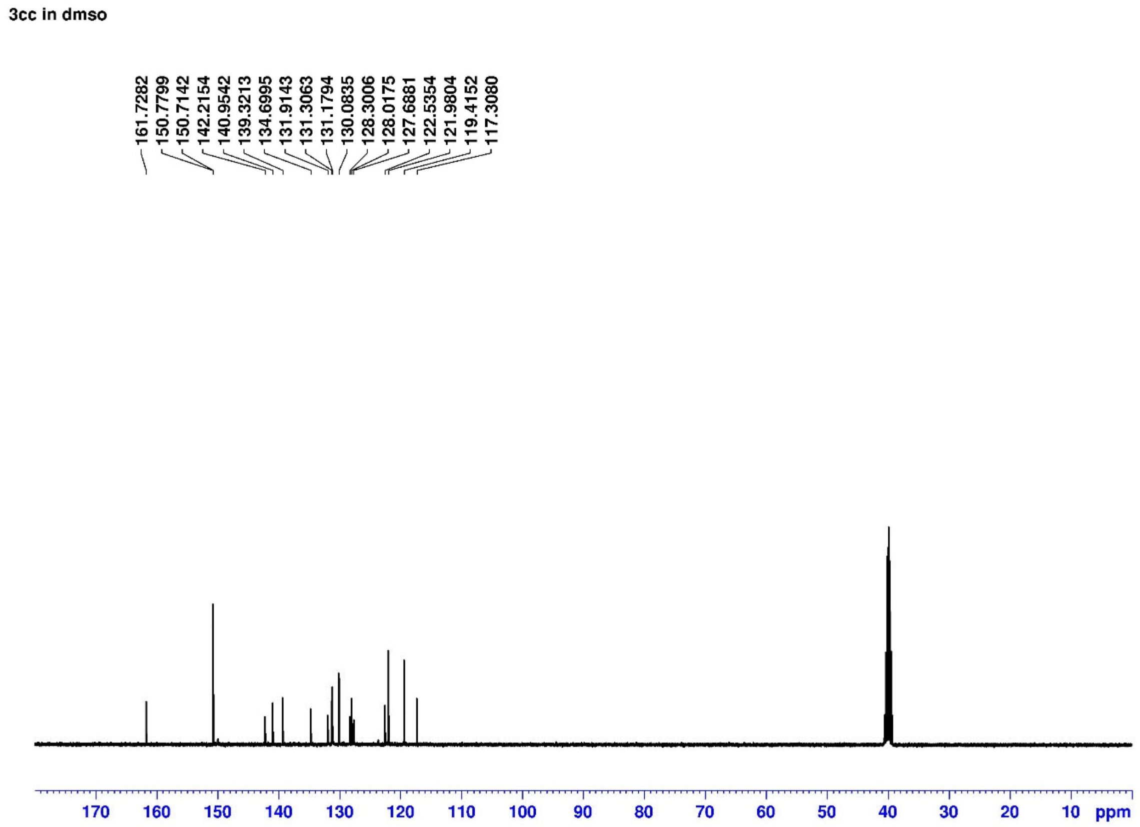

^{13}CNMR spectra of **6h**.

Elemental Composition Report

Single Mass Analysis

Tolerance = 5.0 PPM / DBE: min = -5.0, max = 50.0
Element prediction: Off
Number of isotope peaks used for i-FIT = 3

Monoisotopic Mass, Even Electron Ions
162 formula(e) evaluated with 1 results within limits (all results (up to 1000) for each mass)
Elements Used:
C: 0-80 H: 0-100 N: 5-10 O: 0-5 Na: 1-1 Br: 1-1

MG_3CC 204 (1.765) Cm (1:230)
TOF MS ES+

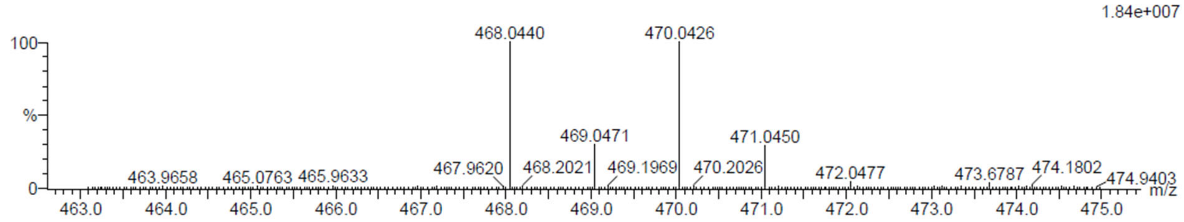

Minimum:				-5.0				
Maximum:		100.0	5.0	50.0				
Mass	Calc. Mass	mDa	PPM	DBE	i-FIT	Norm	Conf(%)	Formula
468.0440	468.0436	0.4	0.9	16.5	1253.4	n/a	n/a	C22 H16 N5 O Na Br

HRMS of **6h**.

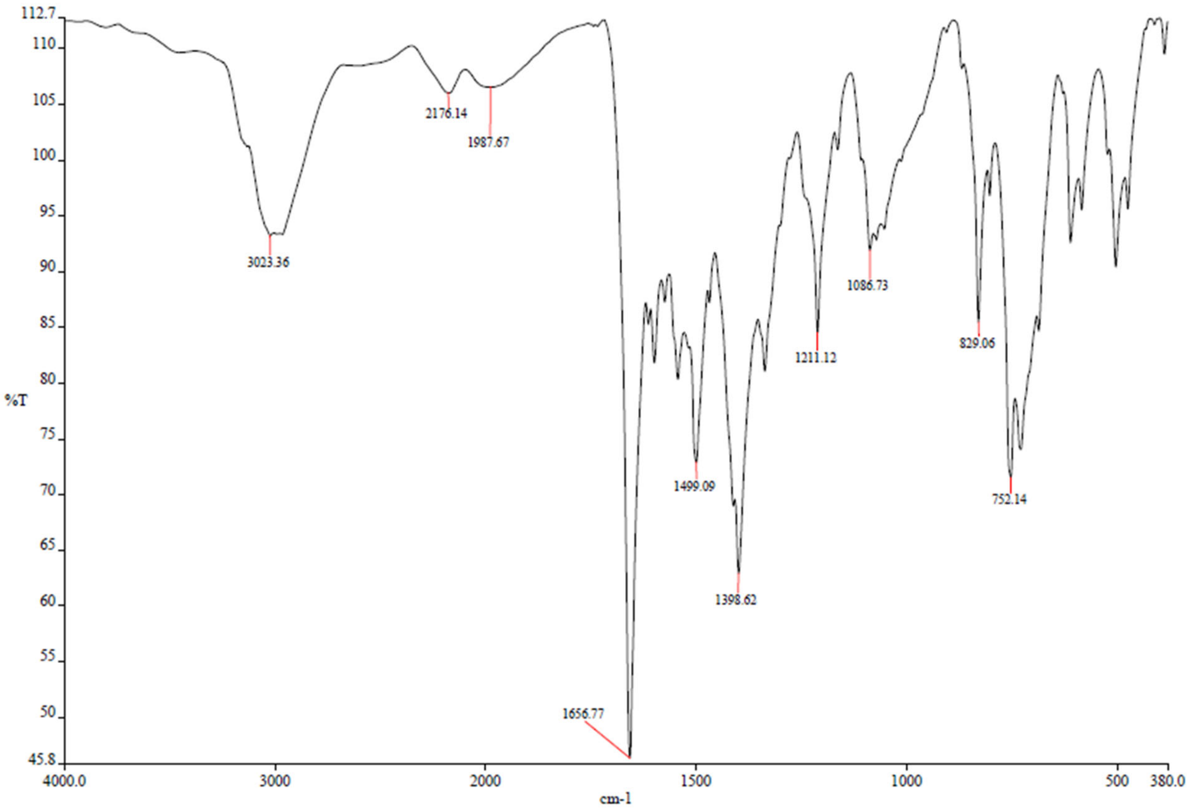

IR spectra of **6i**.

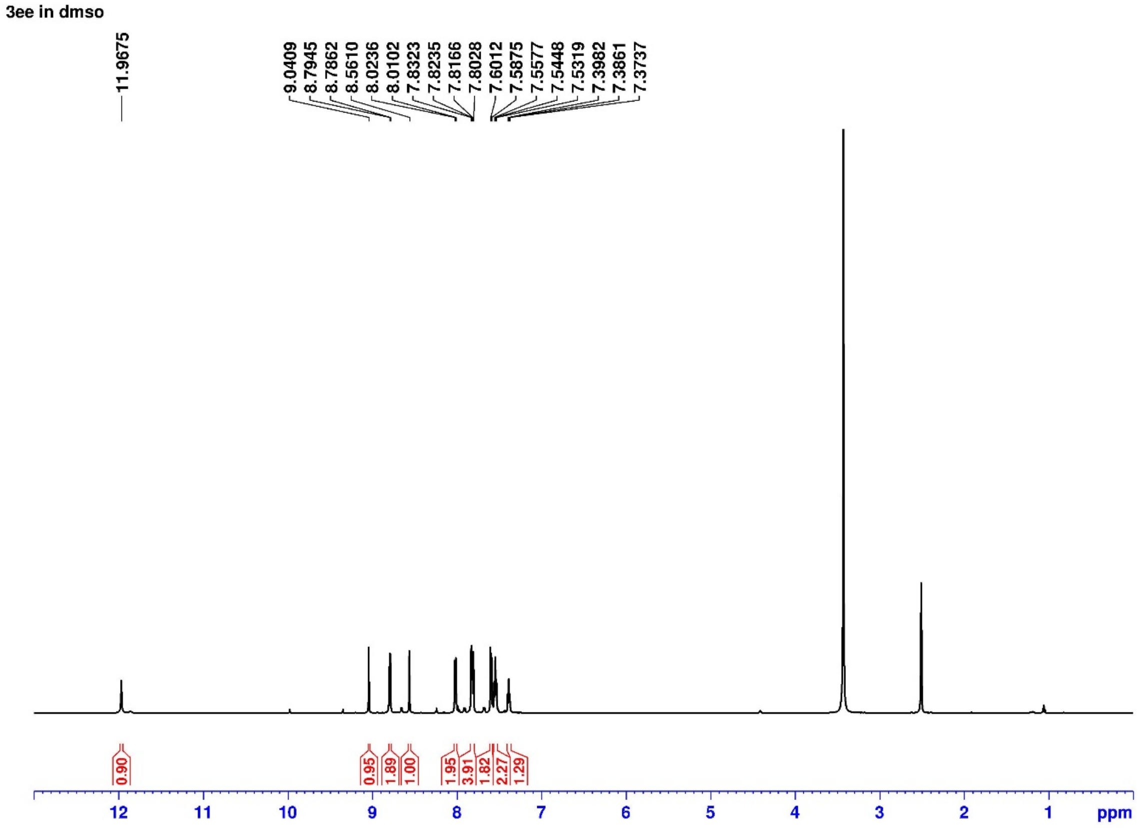

^{1}HNMR spectra of **6i**.

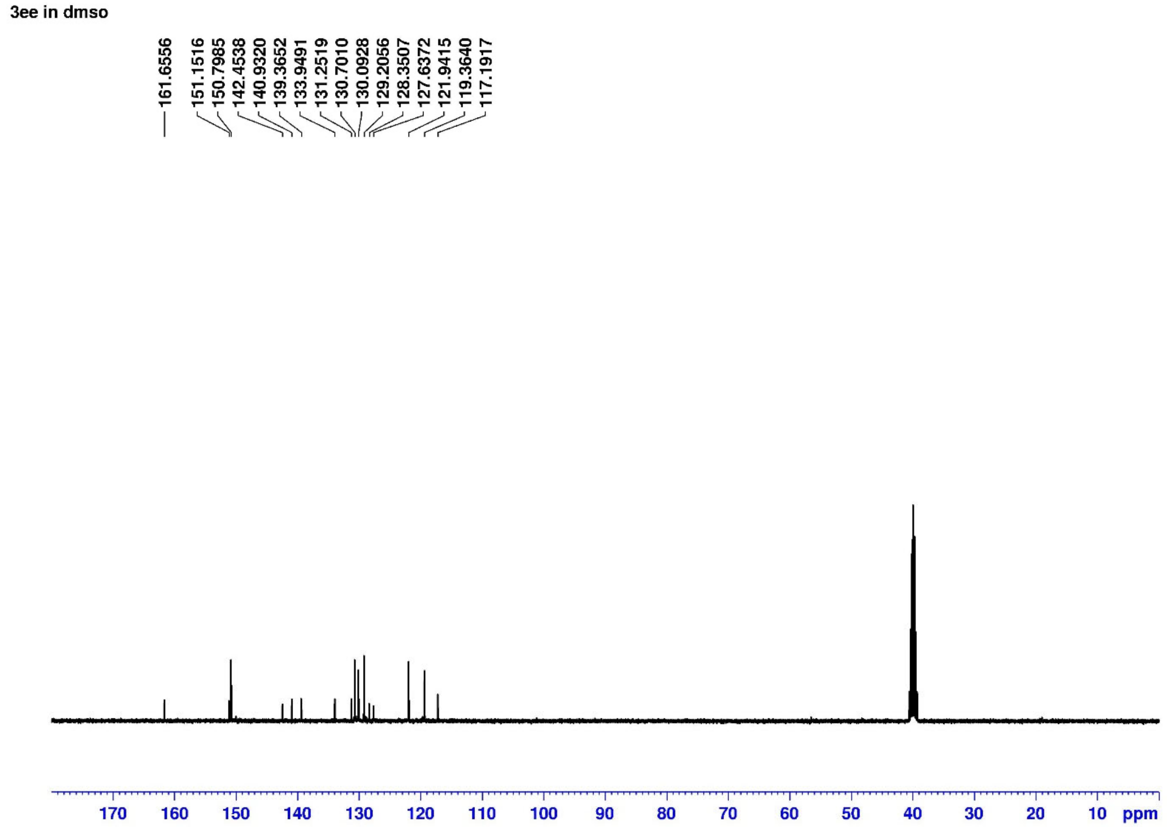

^{13}CNMR spectra of **6i**.

Elemental Composition Report **Page 1**

Single Mass Analysis
Tolerance = 5.0 PPM / DBE: min = -5.0, max = 50.0
Element prediction: Off
Number of isotope peaks used for i-FIT = 3

Monoisotopic Mass, Even Electron Ions
162 formula(e) evaluated with 1 results within limits (all results (up to 1000) for each mass)
Elements Used:
C: 0-80 H: 0-100 N: 5-10 O: 0-5 Na: 1-1 Cl: 1-1
MG 3EE 222 (1.919) Cm (1:230)
TOF MS ES+

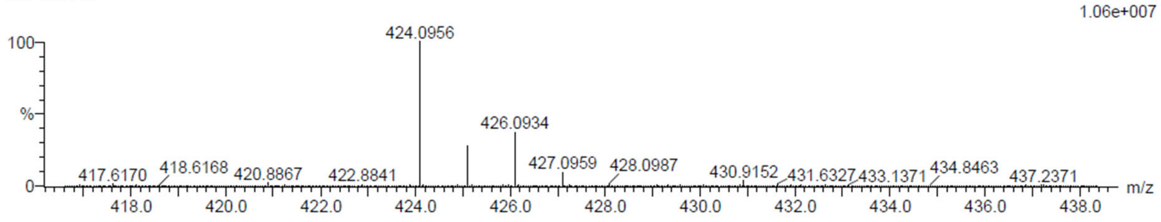

Mass	Calc. Mass	mDa	PPM	DBE	i-FIT	Norm	Conf(%)	Formula
Minimum:				-5.0				
Maximum:		100.0	5.0	50.0				
424.0956	424.0941	1.5	3.5	16.5	1106.9	n/a	n/a	C22 H16 N5 O Na Cl

HRMS of **6i**.

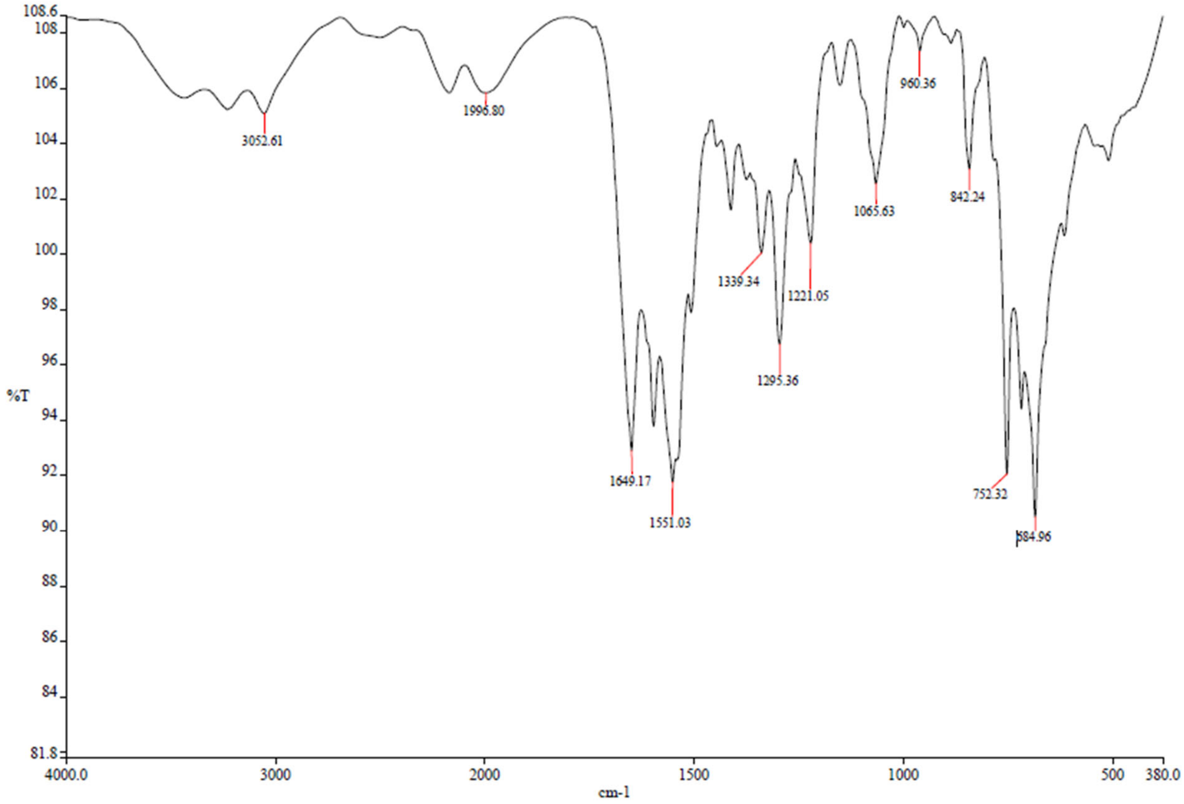

IR spectra of **6j**.

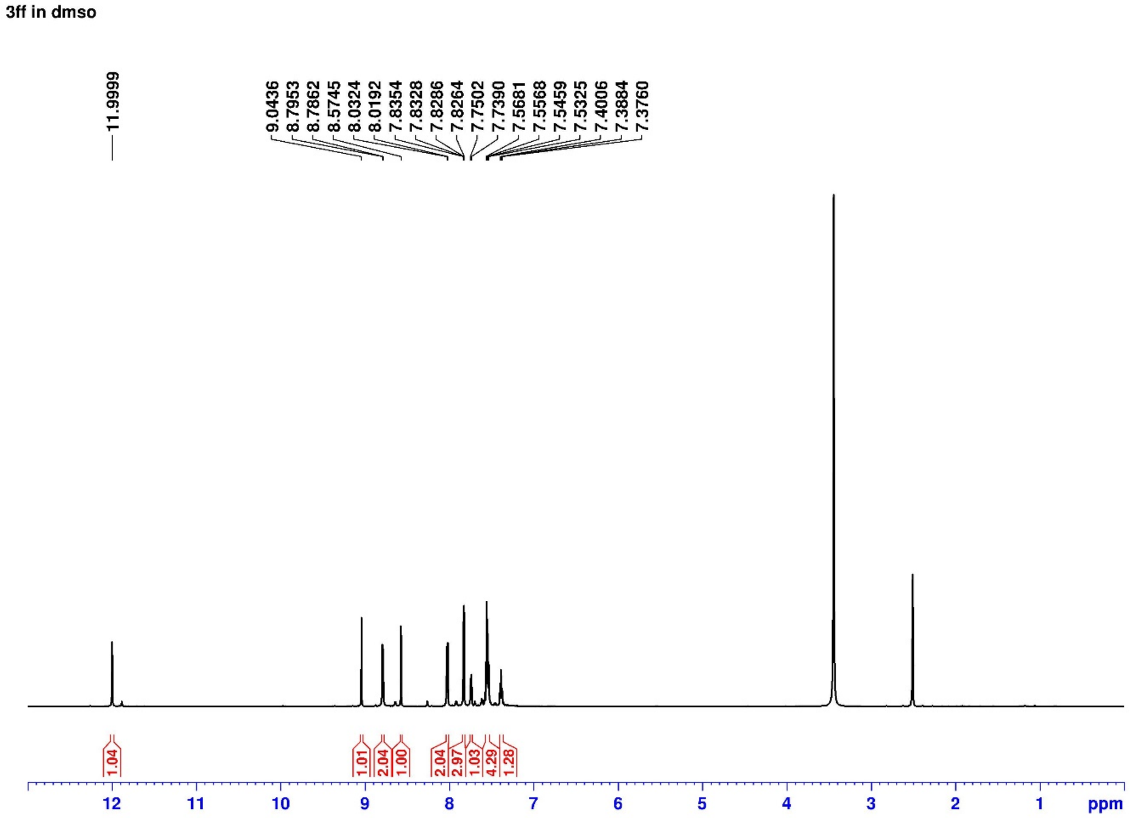

^{1}HNMR spectra of **6j**.

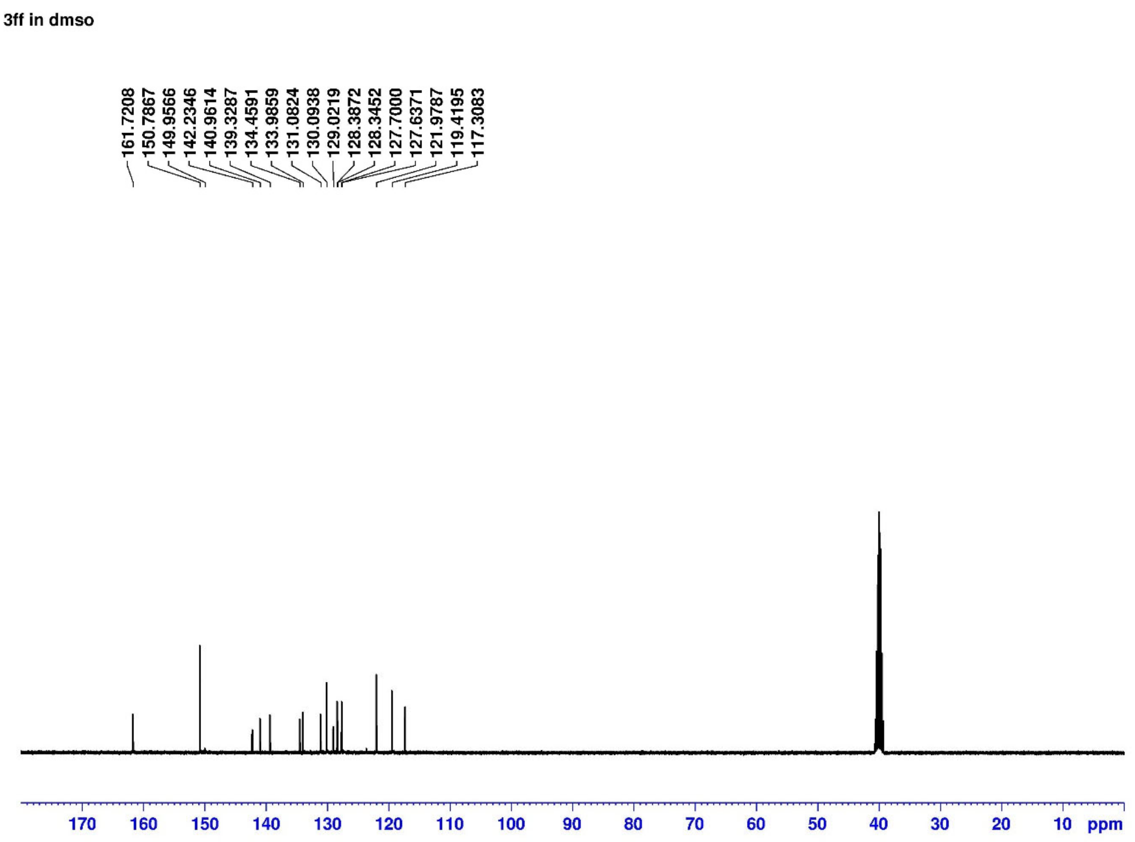

^{13}CNMR spectra of **6j**.

Elemental Composition Report **Page 1**

Single Mass Analysis

Tolerance = 5.0 PPM / DBE: min = -5.0, max = 50.0

Element prediction: Off

Number of isotope peaks used for i-FIT = 3

Monoisotopic Mass, Even Electron Ions

162 formula(e) evaluated with 1 results within limits (all results (up to 1000) for each mass)

Elements Used:

C: 0-80 H: 0-100 N: 5-10 O: 0-5 Na: 1-1 Cl: 1-1

MG 3FF 162 (1.405) Cm (1:230)

TOF MS ES+

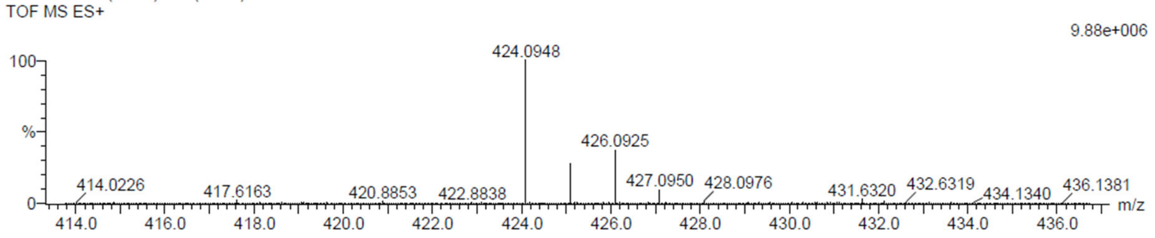

Minimum:				-5.0				
Maximum:		100.0	5.0	50.0				
Mass	Calc. Mass	mDa	PPM	DBE	i-FIT	Norm	Conf(%)	Formula
424.0948	424.0941	0.7	1.7	16.5	1160.7	n/a	n/a	C22 H16 N5 O Na Cl

HRMS of **6j.**

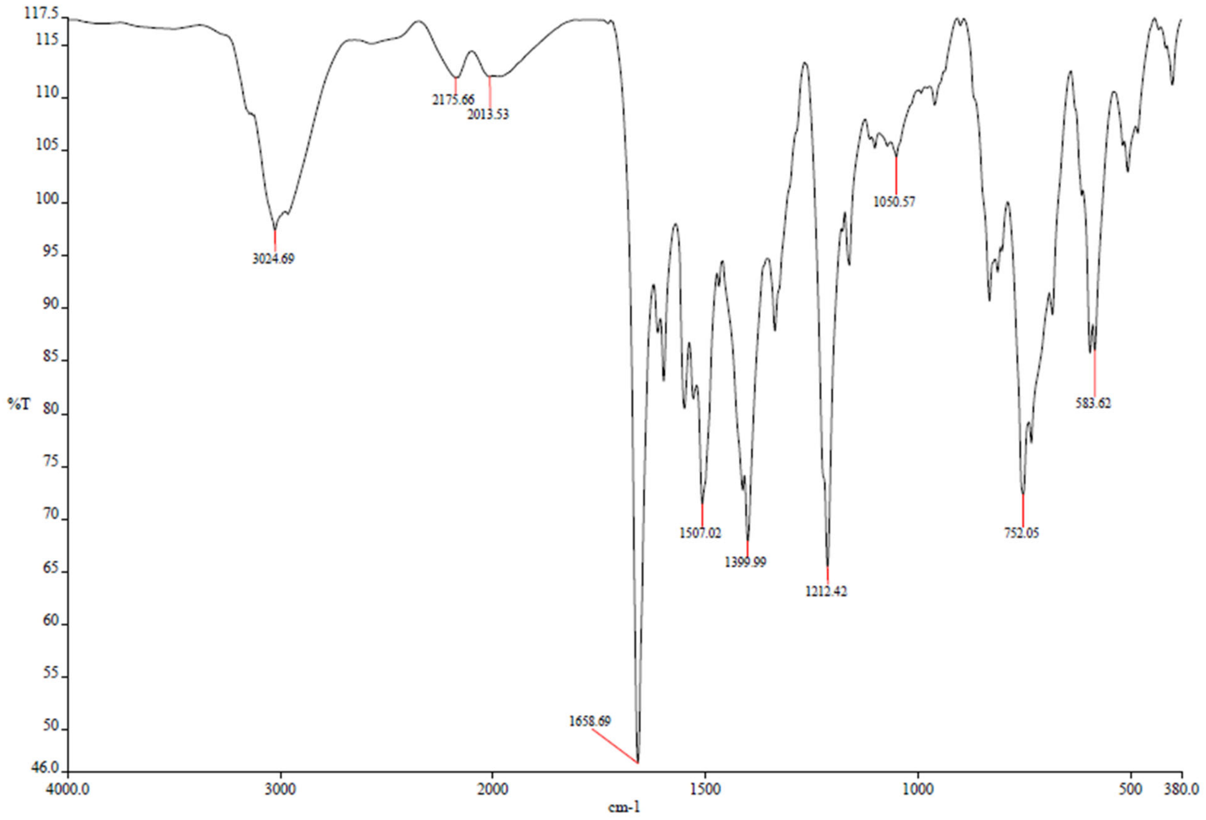

IR spectra of **6k**.

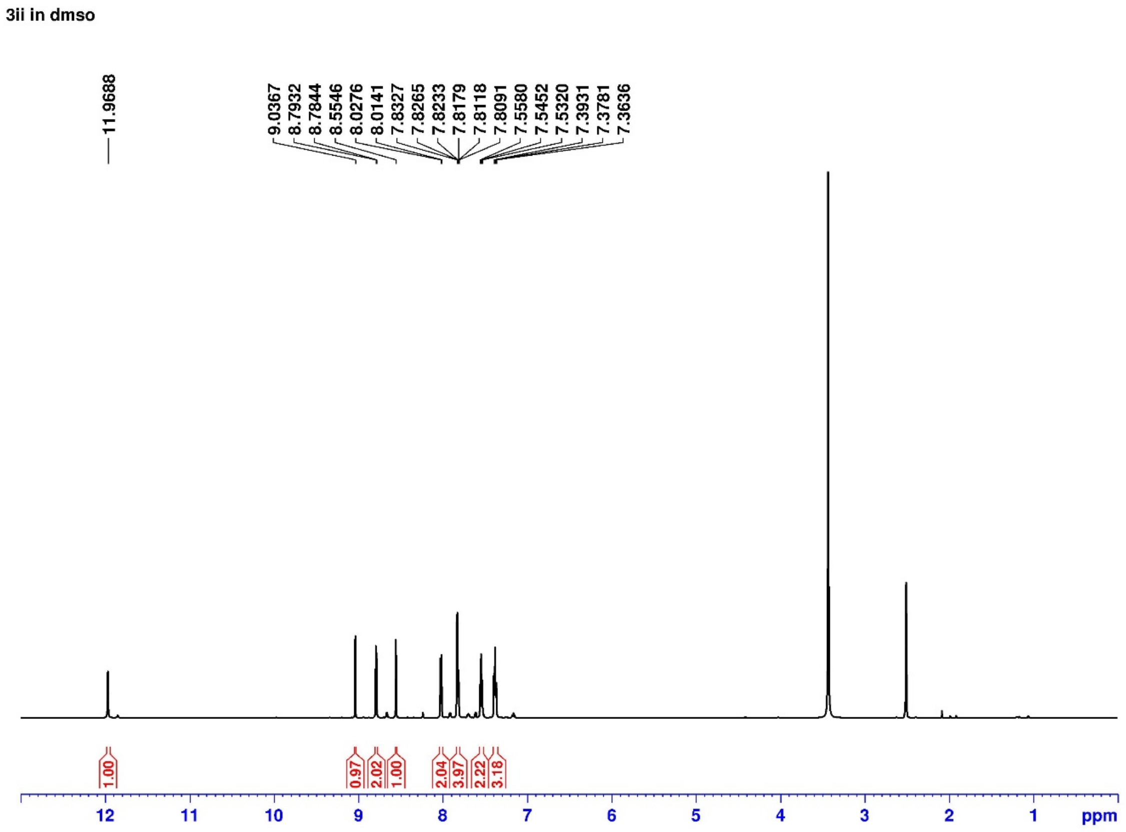

^{1}HNMR spectra of **6k**.

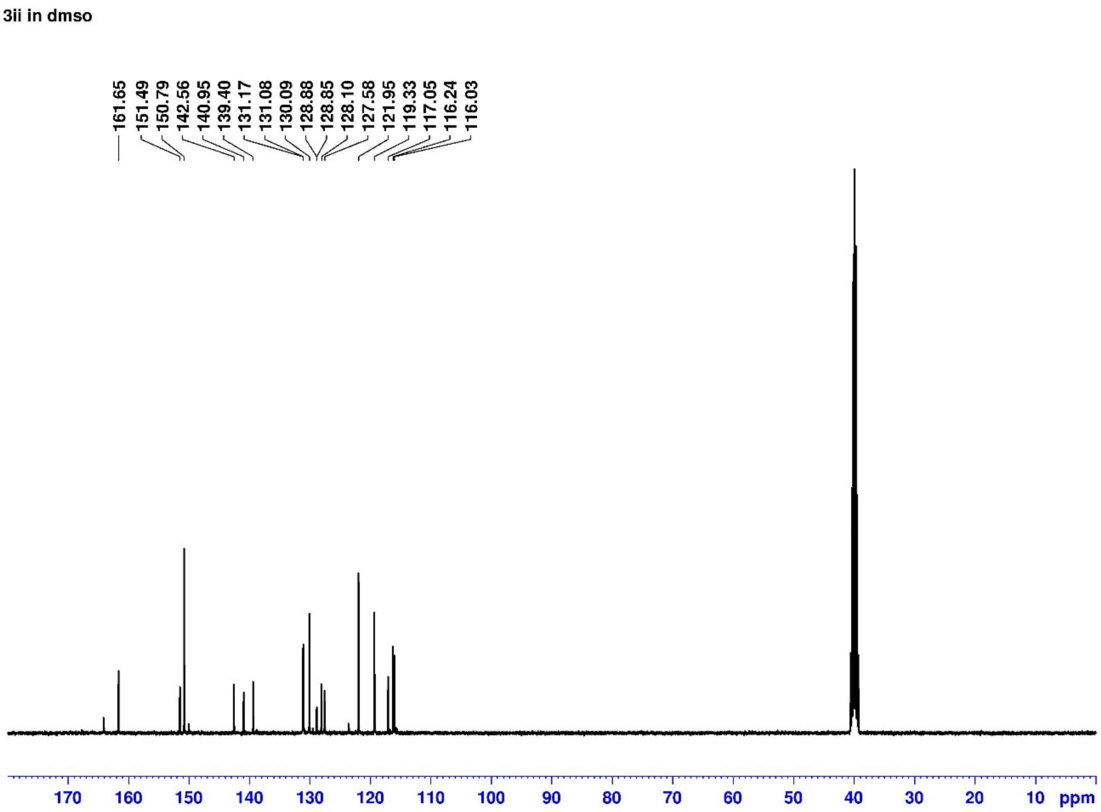

^{13}CNMR spectra of **6k**.

Elemental Composition Report

Page 1

Single Mass Analysis

Tolerance = 5.0 PPM / DBE: min = -5.0, max = 50.0
Element prediction: Off
Number of isotope peaks used for i-FIT = 3

Monoisotopic Mass, Even Electron Ions
162 formula(e) evaluated with 1 results within limits (all results (up to 1000) for each mass)
Elements Used:
C: 0-80 H: 0-100 N: 5-10 O: 0-5 F: 1-1 Na: 1-1
MG 3II 221 (1.911) Cm (1:230)
TOF MS ES+

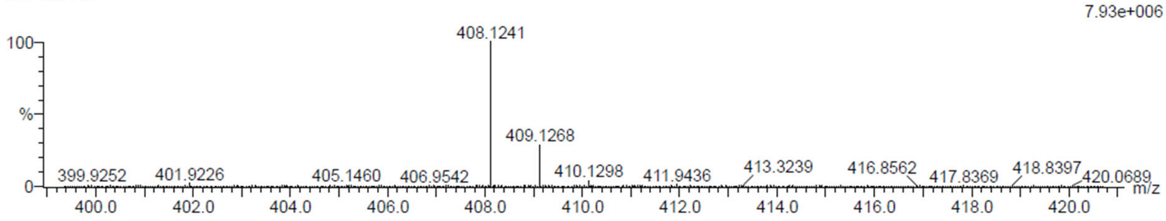

Minimum:				-5.0				
Maximum:		100.0	5.0	50.0				
Mass	Calc. Mass	mDa	PPM	DBE	i-FIT	Norm	Conf(%)	Formula
408.1241	408.1237	0.4	1.0	16.5	1116.5	n/a	n/a	C22 H16 N5 O F Na

HRMS of **6k**.

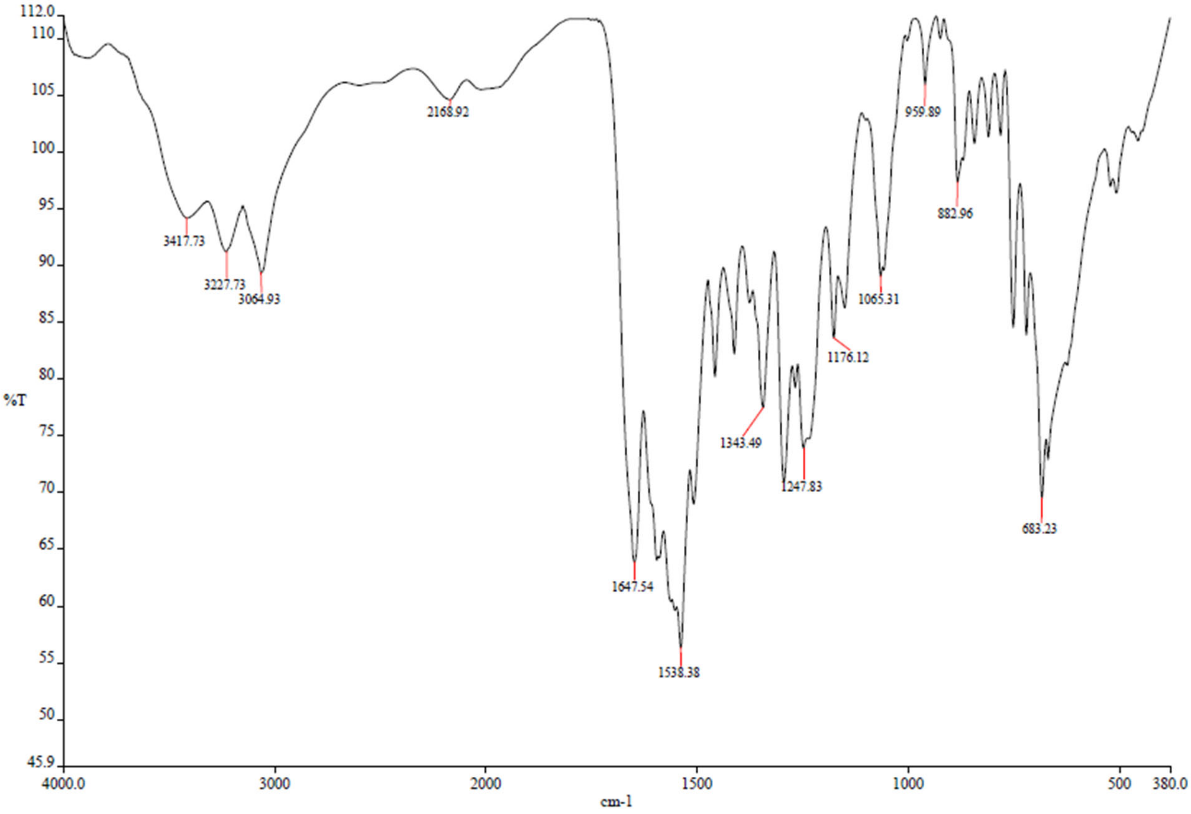

IR spectra of **6l**.

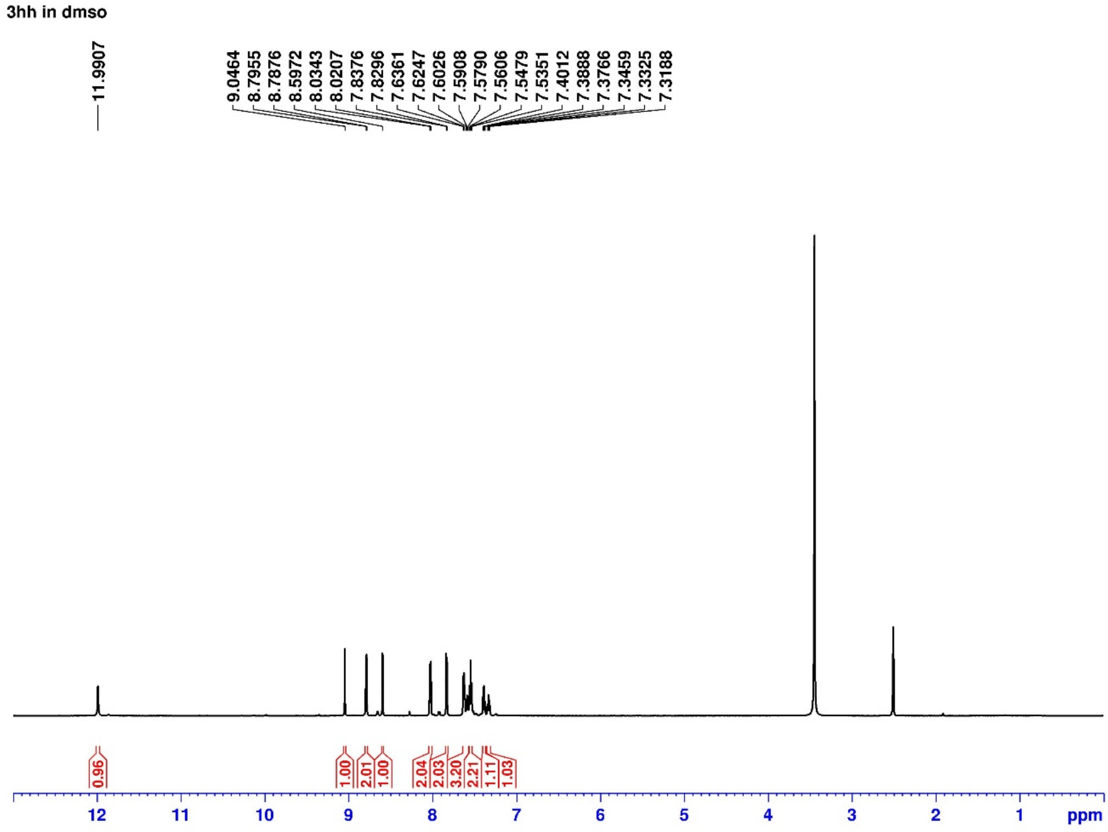

^{1}HNMR spectra of **6l**.

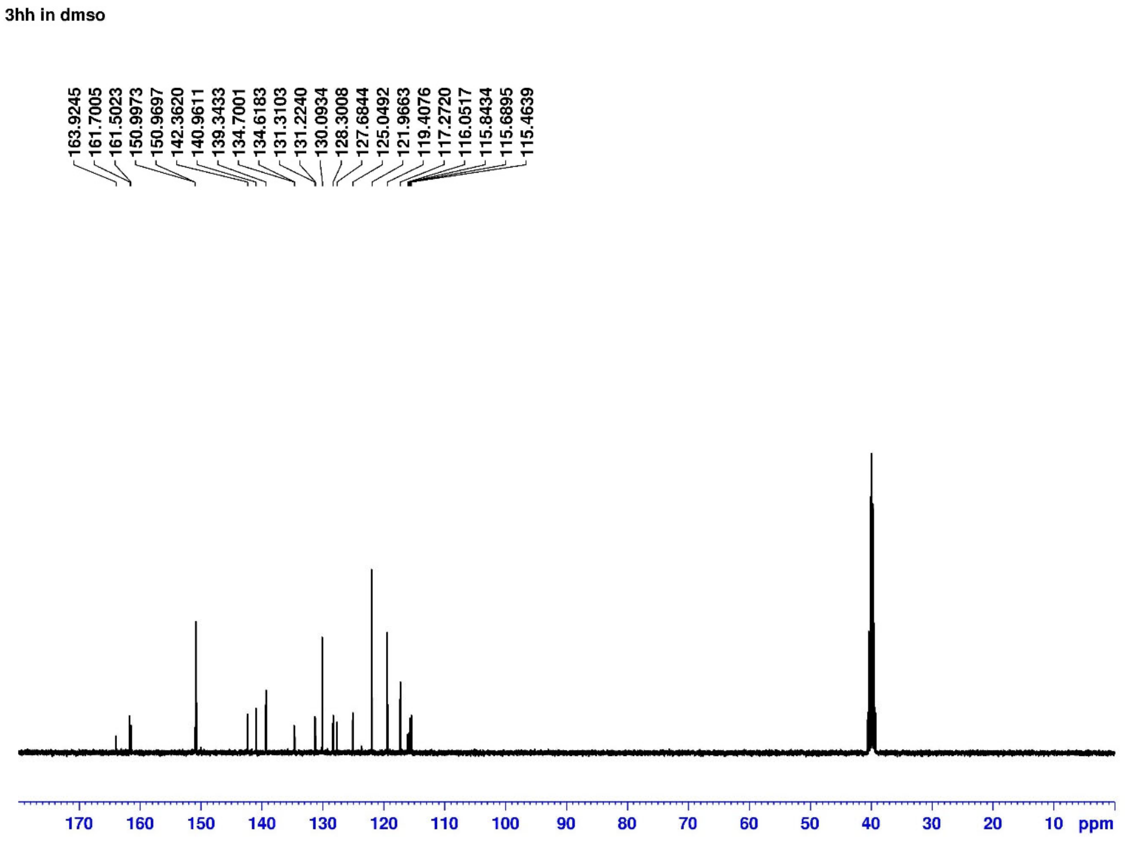

^{13}CNMR spectra of **6l**.

Elemental Composition Report

Single Mass Analysis

Tolerance = 5.0 PPM / DBE: min = -5.0, max = 50.0
Element prediction: Off
Number of isotope peaks used for i-FIT = 3

Monoisotopic Mass, Even Electron Ions
162 formula(e) evaluated with 1 results within limits (all results (up to 1000) for each mass)
Elements Used:
C: 0-80 H: 0-100 N: 5-10 O: 0-5 Na: 1-1 F: 1-1

MG_3HH 43 (0.384) Cm (1:230)
TOF MS ES+

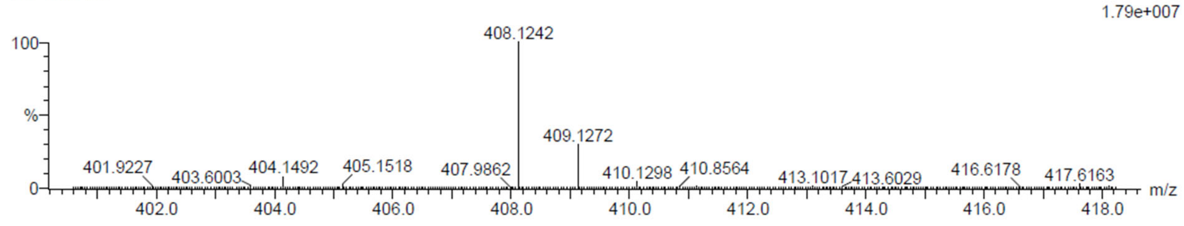

Minimum:				-5.0				
Maximum:		100.0	5.0	50.0				
Mass	Calc. Mass	mDa	PPM	DBE	i-FIT	Norm	Conf(%)	Formula
408.1242	408.1237	0.5	1.2	16.5	1130.7	n/a	n/a	C22 H16 N5 O Na F

HRMS of **6l**.

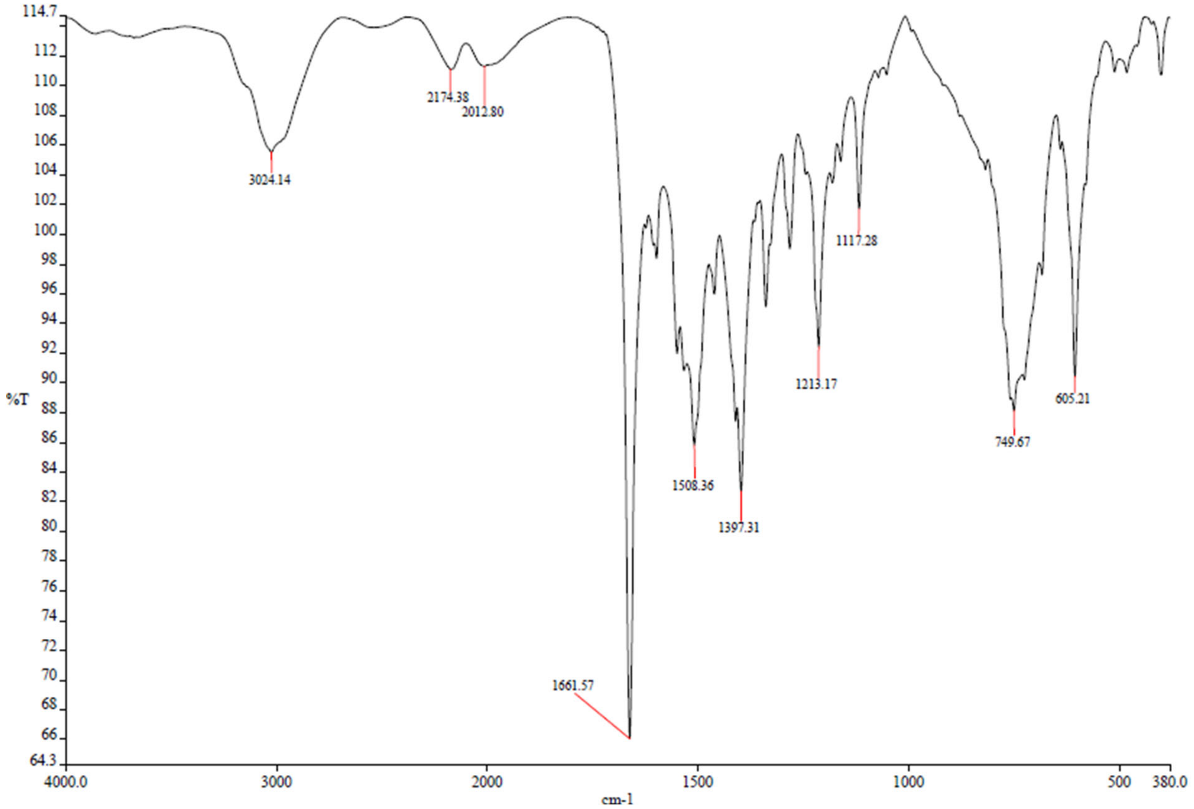

IR spectra of **6m**.

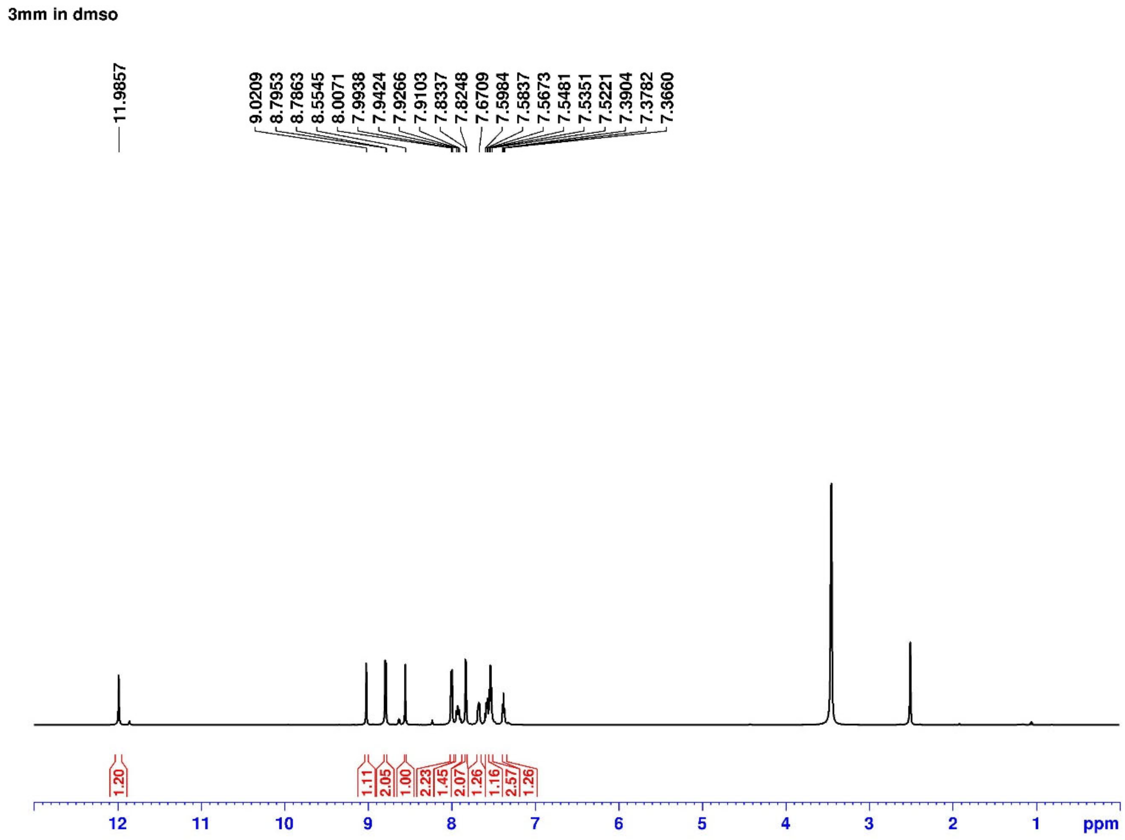

^{1}HNMR spectra of **6m**.

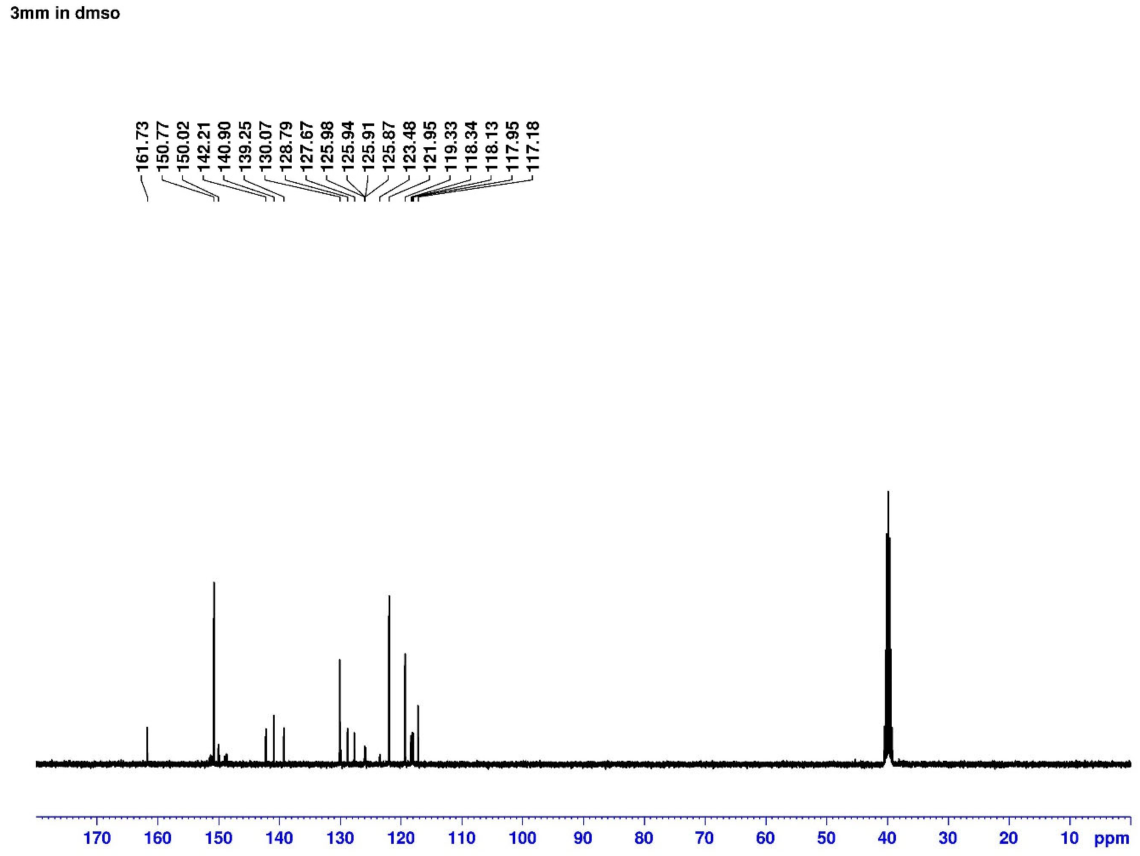

^{13}CNMR spectra of **6m**.

Elemental Composition Report **Page 1**

Single Mass Analysis
Tolerance = 5.0 PPM / DBE: min = -5.0, max = 50.0
Element prediction: Off
Number of isotope peaks used for i-FIT = 3

Monoisotopic Mass, Even Electron Ions
66 formula(e) evaluated with 1 results within limits (all results (up to 1000) for each mass)
Elements Used:
C: 0-80 H: 0-100 N: 0-5 O: 0-1 Na: 1-1 F: 2-2

MG_3MM 231 (1.997) Cm (1:231)
TOF MS ES+

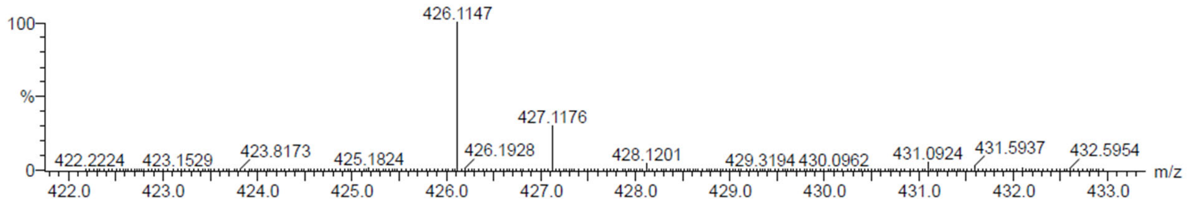

Minimum:				-5.0				
Maximum:		100.0	5.0	50.0				
Mass	Calc. Mass	mDa	PPM	DBE	i-FIT	Norm	Conf(%)	Formula
426.1147	426.1142	0.5	1.2	16.5	1201.1	n/a	n/a	C22 H15 N5 O Na F2

HRMS of **6m**.

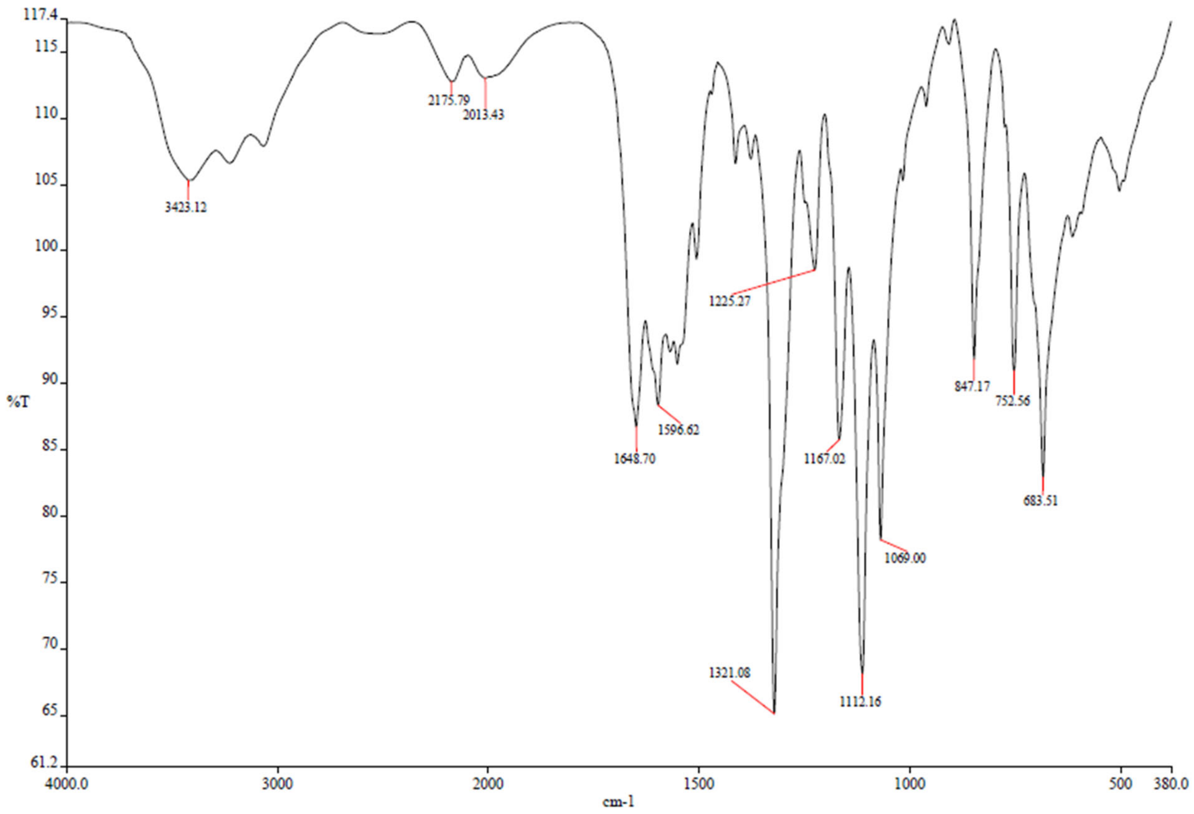

IR spectra of **6n**.

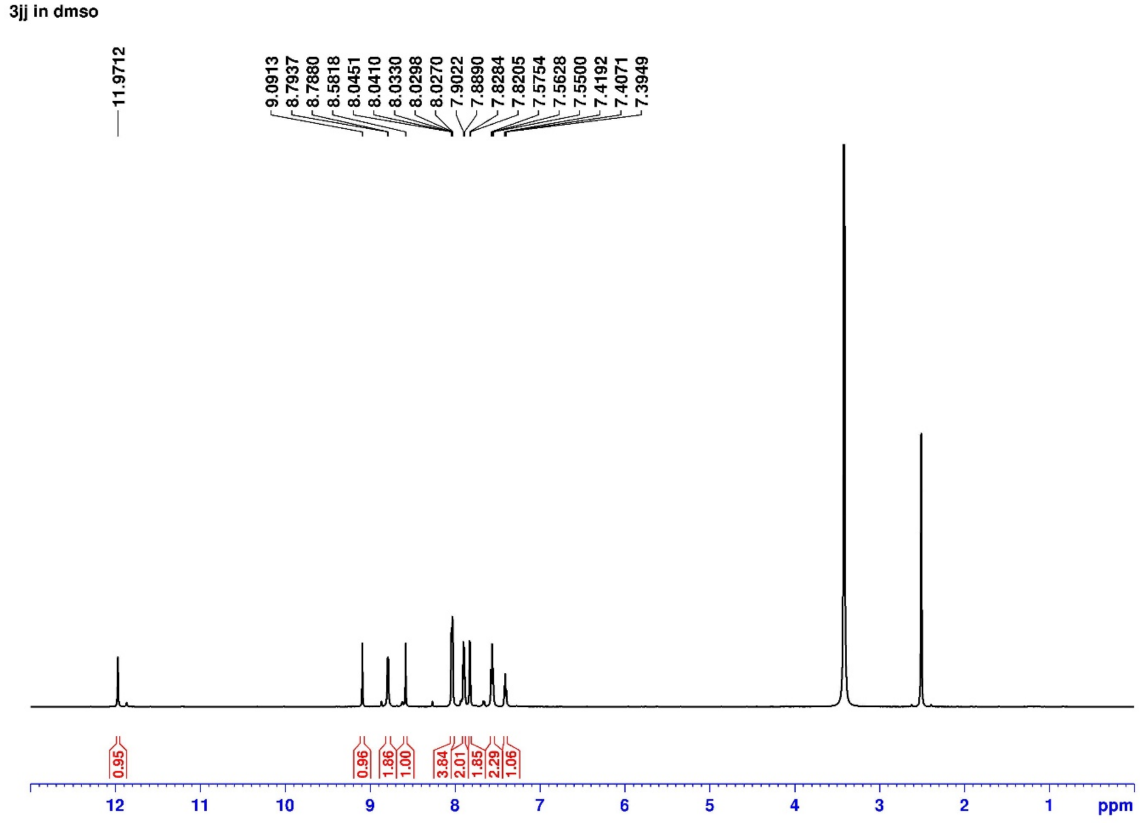

^{1}HNMR spectra of **6n**.

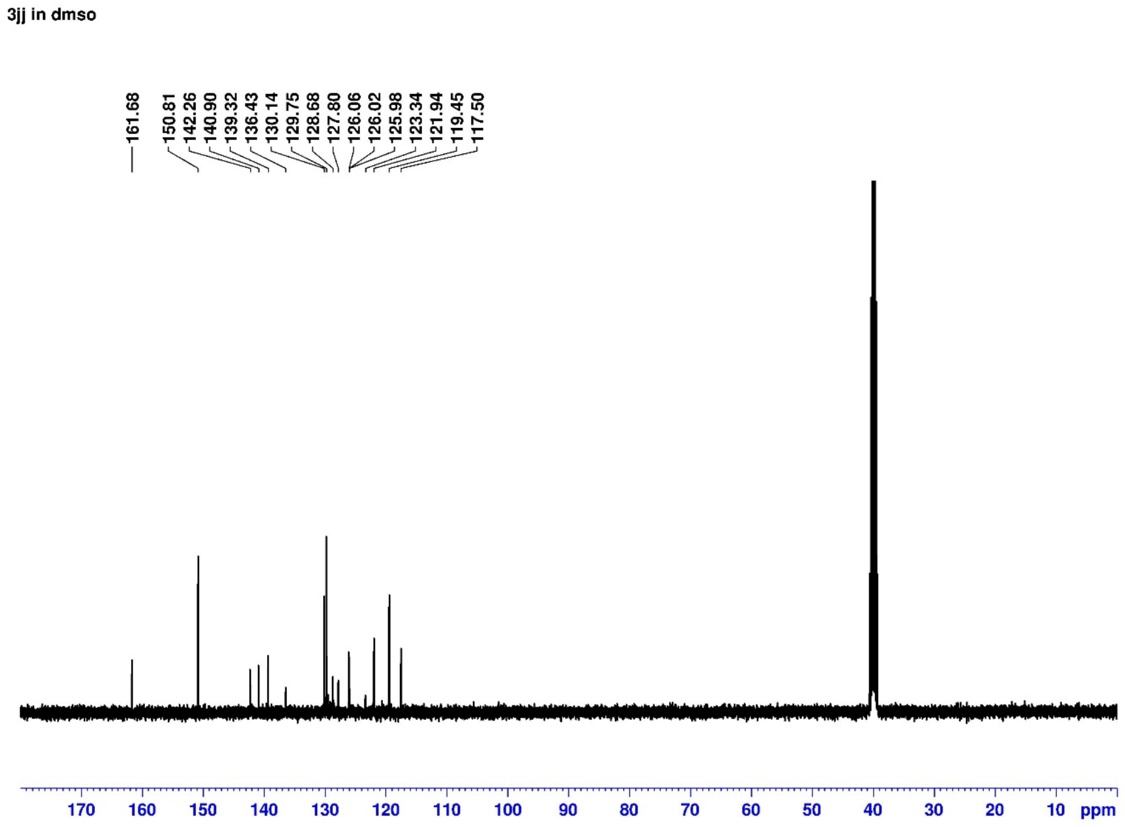

^{13}CNMR spectra of **6n**.

Elemental Composition Report

Page 1

Single Mass Analysis

Tolerance = 5.0 PPM / DBE: min = -5.0, max = 50.0
Element prediction: Off
Number of isotope peaks used for i-FIT = 3

Monoisotopic Mass, Even Electron Ions
162 formula(e) evaluated with 1 results within limits (all results (up to 1000) for each mass)
Elements Used:
C: 0-80 H: 0-100 N: 5-10 O: 0-5 F: 3-3 Na: 1-1
MG 3JJ 104 (0.907) Cm (1:230)
TOF MS ES+

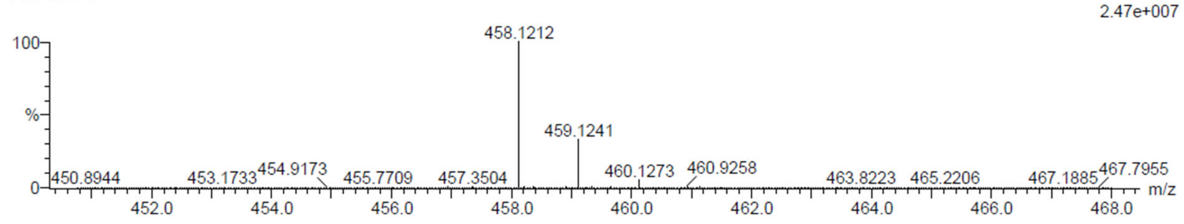

Minimum:				-5.0				
Maximum:		100.0	5.0	50.0				
Mass	Calc. Mass	mDa	PPM	DBE	i-FIT	Norm	Conf(%)	Formula
458.1212	458.1205	0.7	1.5	16.5	1254.4	n/a	n/a	C23 H16 N5 O F3 Na

HRMS of **6n**.

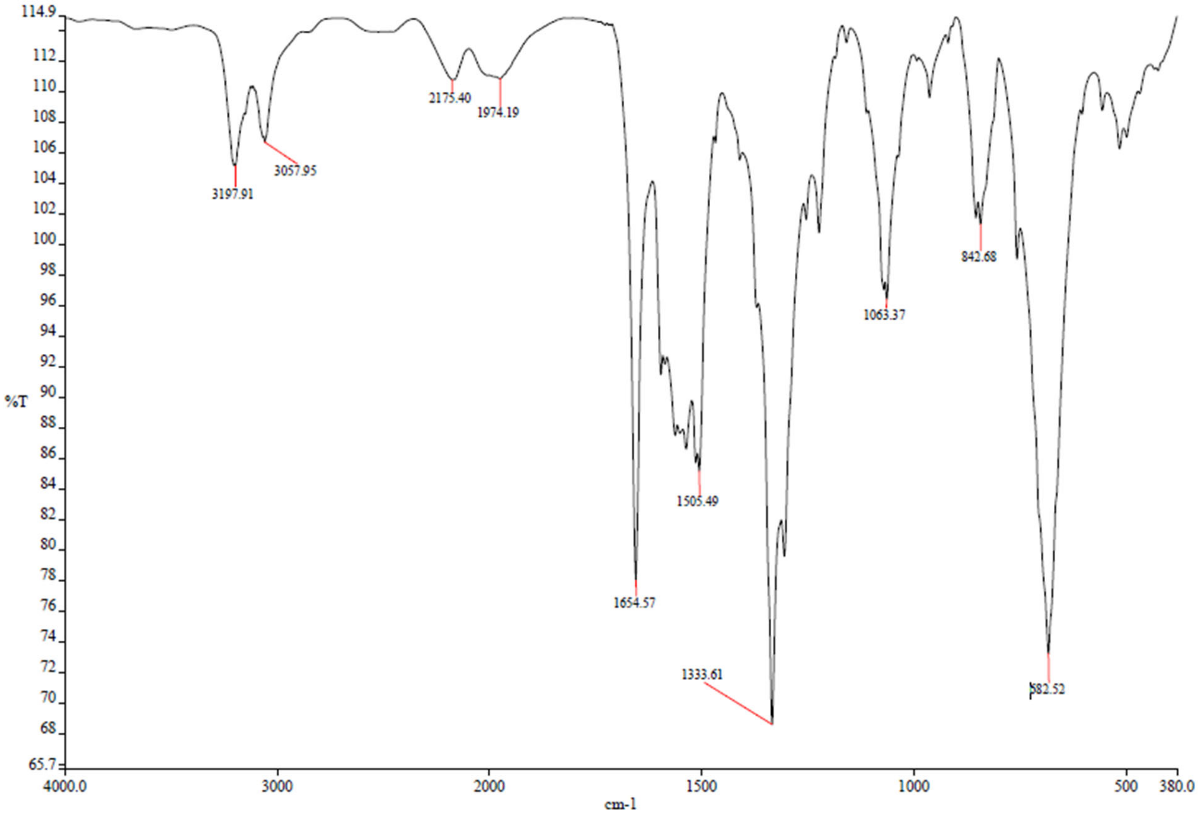

IR spectra of **6o**.

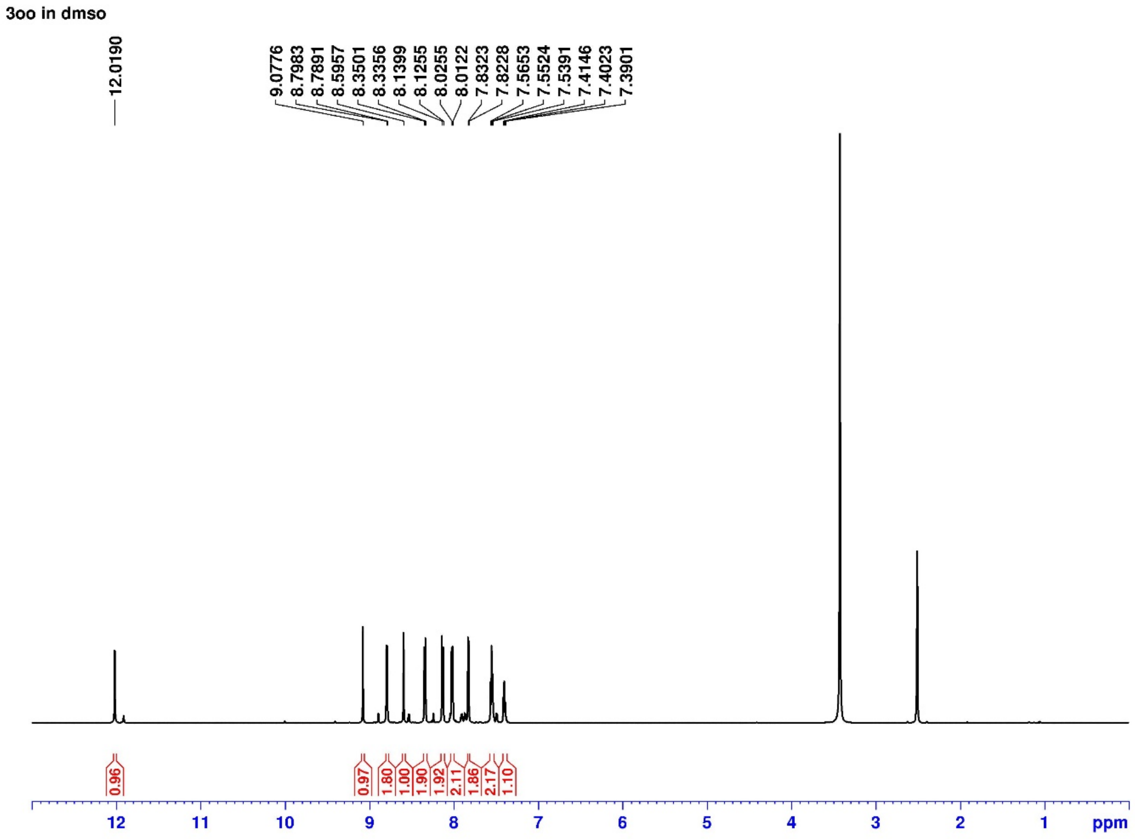

^{1}HNMR spectra of **6o**.

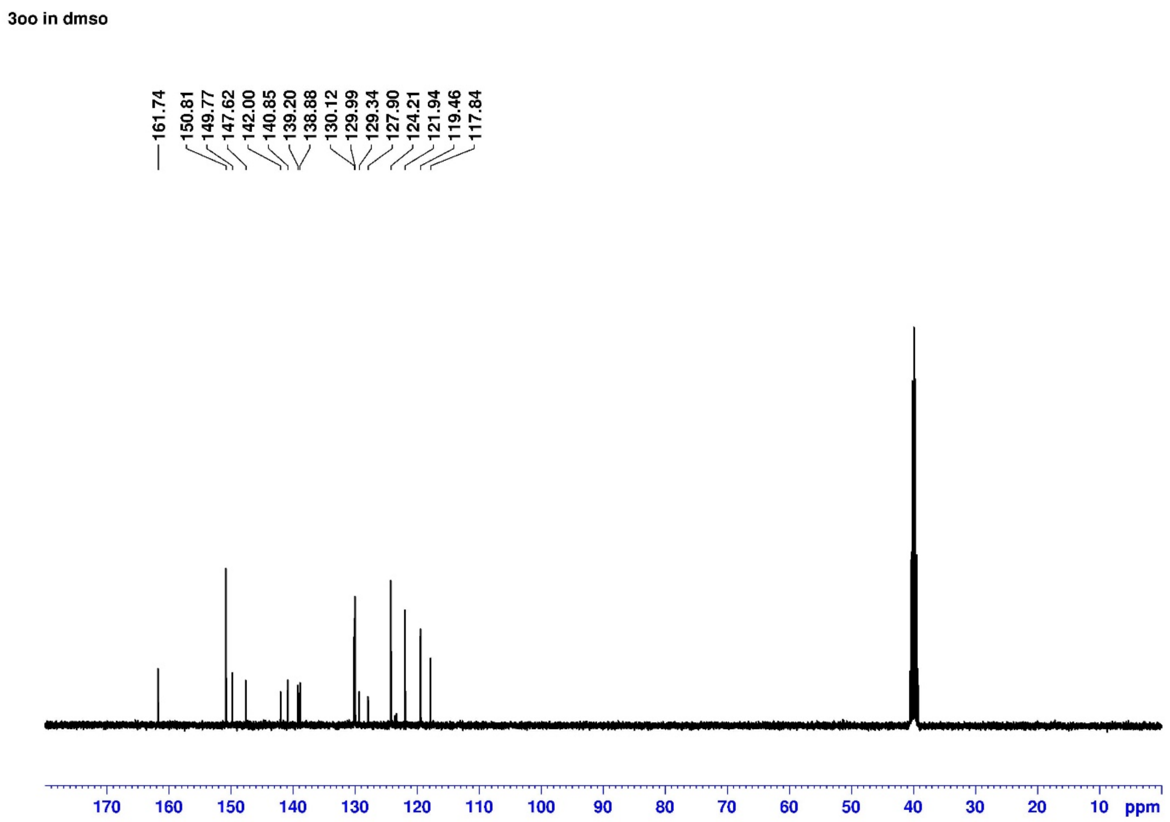

^{13}CNMR spectra of **6o**.

Elemental Composition Report **Page 1**

Single Mass Analysis
Tolerance = 5.0 PPM / DBE: min = -5.0, max = 50.0
Element prediction: Off
Number of isotope peaks used for i-FIT = 3

Monoisotopic Mass, Even Electron Ions
318 formula(e) evaluated with 1 results within limits (all results (up to 1000) for each mass)
Elements Used:
C: 0-80 H: 0-100 N: 1-10 O: 0-5 Na: 1-1
MG 3OO 30 (0.273) Cm (1:230)
TOF MS ES+

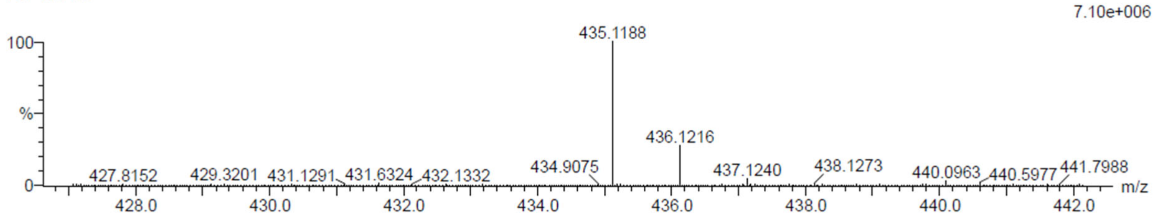

Minimum:				-5.0				
Maximum:		100.0	5.0	50.0				
Mass	Calc. Mass	mDa	PPM	DBE	i-FIT	Norm	Conf(%)	Formula
435.1188	435.1182	0.6	1.4	17.5	1079.5	n/a	n/a	C22 H16 N6 O3 Na

HRMS of **6o**.
